# Localized immune surveillance of primary melanoma in the skin deciphered through executable modeling

**DOI:** 10.1126/sciadv.add1992

**Published:** 2023-04-12

**Authors:** Rowan Howell, James Davies, Matthew A. Clarke, Anna Appios, Inês Mesquita, Yashoda Jayal, Ben Ringham-Terry, Isabel Boned Del Rio, Jasmin Fisher, Clare L. Bennett

**Affiliations:** UCL Cancer Institute, University College London, 72 Huntley Street, London WC1E 6DD, UK.

## Abstract

While skin is a site of active immune surveillance, primary melanomas often escape detection. Here, we have developed an in silico model to determine the local cross-talk between melanomas and Langerhans cells (LCs), the primary antigen-presenting cells at the site of melanoma development. The model predicts that melanomas fail to activate LC migration to lymph nodes until tumors reach a critical size, which is determined by a positive TNF-α feedback loop within melanomas, in line with our observations of murine tumors. In silico drug screening, supported by subsequent experimental testing, shows that treatment of primary tumors with MAPK pathway inhibitors may further prevent LC migration. In addition, our in silico model predicts treatment combinations that bypass LC dysfunction. In conclusion, our combined approach of in silico and in vivo studies suggests a molecular mechanism that explains how early melanomas develop under the radar of immune surveillance by LC.

## Teaser

Computational modelling defines melanoma-Langerhans cell crosstalk in the skin.

## INTRODUCTION

Treatment of metastatic melanoma has been revolutionized over the past decade with the emergence of immune checkpoint therapy, but it remains a deadly disease, accounting for 75% of skin cancer deaths despite only making up 5% of skin cancer cases (*1*). Surgical removal of primary melanomas is an effective treatment, with a 99% 5-year survival rate in patients presenting with localized disease. However, prognosis deteriorates rapidly to 68% once melanoma cells escape the skin and spread to regional lymph nodes (LNs) and 30% in patients with systemic metastatic disease (*2*). Moreover, survival rates are decreased in patients with multiple primary tumors (*3*). Improving our understanding of the immune response to primary melanoma could help improve care for patients with early-stage melanoma and shed light on the evolutionary pressures shaping metastatic melanoma through immune editing.

Primary melanomas develop in the skin, a site of active immune monitoring of environmental insults. The effectiveness of immune checkpoint therapy in melanoma patients compared to other cancers has been attributed to the immunogenicity of metastatic melanomas; this immune reactivity can result from production of melanoma-specific differentiation antigens, such as MART1, and generation of neoantigens due to the high ultraviolet (UV)–induced mutational burden in melanomas (*4*). This raises the question, therefore, of why primary melanomas do not activate robust immune surveillance in the skin. Localized adjuvant immunotherapy before surgical resection of the primary tumor has shown promise in limiting regional metastasis and improving recurrence-free survival (*5*), and T cell infiltration into primary melanomas correlates with a reduced risk of recurrent metastatic disease (*6*), suggesting that activation of skin immunity is beneficial to patients. However, we understand little about how the cutaneous immune system interacts with growing melanomas in situ. Improving our understanding of the mechanisms of potential immune avoidance by melanomas in the epidermis may allow for the development of improved immunotherapies to augment surgical resection.

The skin is host to a unique system of active immune surveillance, wherein a network of immune cells maintains a balance to ensure detection of invading harmful pathogens while avoiding the initiation of responses to harmless environmental insults. Located in the same basal epidermal region as melanocytes, Langerhans cells (LCs) are the only resident antigen-presenting cells in the epidermis and therefore predicted to be the first immune cells capable of detecting the earliest stages of melanomagenesis. Despite developing from embryonic macrophages, LCs share morphological and functional similarities to dendritic cells (DCs); upon detection of an invading pathogen, LCs detach from surrounding keratinocytes (KCs) and migrate via the dermis through the lymphatic system to draining LNs, where they may interact with naïve T cells (*7*). Migration of LCs is tightly linked to an up-regulation of peptide-loaded surface major histocompatibility complex (MHC) and costimulatory molecules such that LCs are poised to prime naïve T cells in LNs (*8*, *9*). LC residency in the skin and migration to draining LNs are principally controlled by a balance between transforming growth factor–β (TGF-β) and proinflammatory cytokines. Autocrine TGF-β is required for attachment of LCs to KCs, and increased expression of TGF-β is sufficient to trap LCs in the epidermis (*10*). LC activation and release from residency in the epidermis is driven by the production of inflammatory factors, namely, tumor necrosis factor–α (TNF-α) and interleukin-1β (IL-1β), in response to infection or inflammation. In nonmalignant skin, migration is primarily a response to activation of surrounding KCs and infiltration of dermal cytokines (*7*). Binding of TNF-α initiates an immunogenic gene program within LC, leading to activation of an effector T cell response (*11*).

Despite the colocalization of LCs with melanocytes, to date, there is little cellular evidence documenting the interaction between LCs and melanomas in the skin, and clinical studies have focused on LC function after migration to sentinel LNs. LCs isolated from patient LNs containing melanoma cells have an inactivated phenotype, similar to noninvolved LNs at distal sites or may even be immunosuppressive (*12*–*14*), and are inefficient at priming T cells in vitro (*15*). Moreover, a decrease in the expression of human leukocyte antigen (HLA) molecules on LCs in sentinel LNs correlates with increased primary tumor depth (*16*). These studies suggest that migrating LCs are not activated by cutaneous melanomas. However, deciphering the complexity of melanoma-LC interactions in the skin has frequently been hampered by a lack of available primary melanoma material matched with adjacent skin containing LCs. Therefore, we understand little about how epidermal LCs may sense and respond to growing melanomas in situ.

With the development of highly multiplexed imaging techniques, there has been a growing appreciation for the numerous cell types inhabiting tumors and their contributions to patient outcomes. Recent studies have applied these techniques to primary melanomas and have identified, for example, the importance of T cell activation status to classification of tumors (*17*) and key interactions between immunosuppressive macrophages and CD8^+^ T cells (*18*). These findings highlight the need for computational approaches that can represent the behavior of different cell types within the tumor. Existing modeling approaches [reviewed in (*19*)] examine either molecular details of oncogenic pathways [for example, (*20*, *21*)] or use coarse-grained representations of cell states and types [for example, (*22*–*24*)]. To predict the impact of mutations or targeted therapies on cellular interactions, computational models operating across these levels of abstraction are required.

**Fig. 1.**
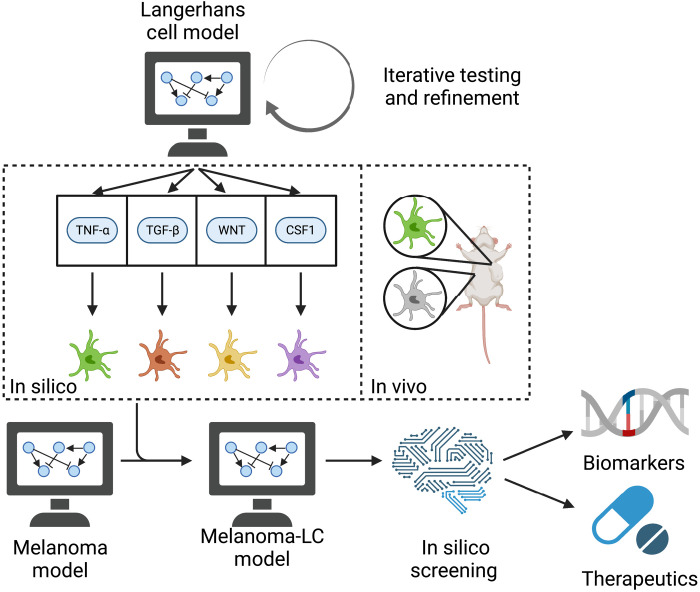
Schematic workflow of melanoma-LC interaction modeling. Publicly available data were used to build and test the LC model. Different hypotheses for the interaction between melanoma and LCs were compared to in vivo measurement of LC behavior to identify the underlying signaling mechanisms. These insights were leveraged to build an integrated melanoma-LC model, which was used to screen for biomarkers of immune evasion and potential therapeutics to treat primary melanoma.

We have adopted an approach integrating experimental investigation of an in vivo preclinical model of melanoma, with computational modeling of LCs and melanoma (Fig. 1). The apparent lack of immune surveillance of growing melanomas in the skin suggests that malignant melanocytes are either ignored or escape recognition by LCs. To probe the molecular mechanisms that could facilitate LC-melanoma cross-talk, we developed an executable model of LC regulation. Executable models can be used to represent biological mechanisms as discrete state transition systems (*25*, *26*) to predict the effects of mutations and drugs on cell phenotype. Executable models have previously been used to provide mechanistic insights and suggest alternative treatment combinations for diseases such as leukemia (*27*, *28*) and breast cancer (*29*), as well as the immune response to coronavirus disease 2019 (COVID-19) (*30*). Here, we developed an executable LC model to generate and test hypotheses about the molecular mechanism of action underpinning evasion of LC control. Our computational model revealed a potential role for tumor-derived TNF-α in the delayed migration of LCs, which was experimentally validated by measurements of TNF-α expression of in vivo tumors. Simultaneously, we developed an executable model of melanoma, linking driver mutations to tumor cell behavior and predicting response to known targeted therapies. By combining the LC and melanoma models, we developed a melanoma-LC executable model based on the impact of melanoma-derived TNF-α on epidermal LCs. In silico drug screening applied to the melanoma-LC executable model revealed that mitogen-activated protein kinase (MAPK) pathway inhibitors such as dabrafenib and trametinib can prevent LC migration, despite effectively inhibiting melanoma cell growth, suggesting that care must be taken with the use of targeted therapies to treat primary tumors. This screening analysis also highlights effective combination therapies that are not predicted to affect LC behavior. Our computational model therefore represents a comprehensive executable map of the signaling underlying immune evasion in the epidermis.

## RESULTS

### Melanoma growth results in delayed migration of LCs to draining LNs

**Fig. 2.**
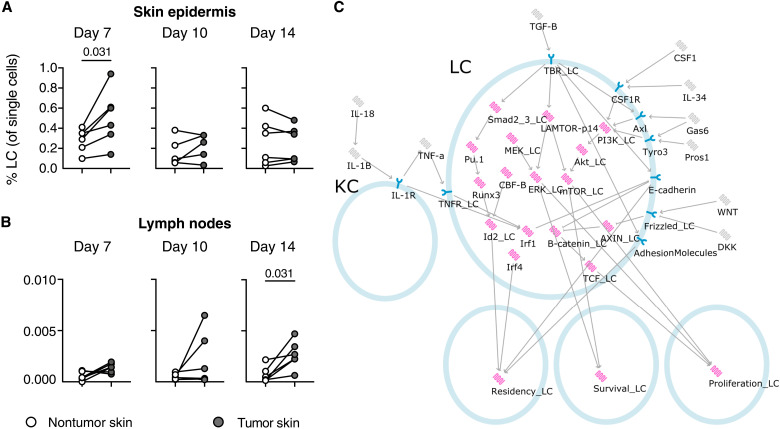
Behavior of LCs in the melanoma-adjacent epidermis. (**A**) Frequency of epidermal LCs (of total single epidermal cells) from skin surrounding tumors or unrelated contralateral skin at indicated time points after injection of YUMM1.7 cells. (**B**) Frequency of LCs (of total single LN cells) in tumor-draining LNs or uninvolved LNs at different time points after tumor injection. For (A) and (B), each point represents individual mice pooled from two independent experiments; *N* = 5 to 6 biological replicates per tissue per time point. Paired data were analyzed using a Wilcoxon matched pairs signed-rank test. White circles show nontumor skin, and gray circles show tumor skin. (**C**) LC network model as seen in the BMA tool. Pink nodes represent proteins or complexes, gray nodes represent secreted proteins, and blue nodes represent receptors. Activating interactions are represented by arrows (→), and inactivating interactions are represented by bars (⟞). TBR_LC refers to the TGF-β receptor in LCs. The _LC suffix is used to distinguish proteins that are also present in the melanoma model (see also Fig. 4).

To determine whether LCs respond to melanoma growth in the epidermis, we established a clinically relevant syngeneic injectable murine melanoma model using the YUMM1.7 (*Braf^V600E/WT^Cdkn2a^−/−^ Pten^−/−^*) cell line (*31*) and measured the frequency of epidermal LCs [CD11b^+^MHCII^+^CD24^+^EpCam^+^ cells; fig. S1 (*32*)] at the tumor site, in contralateral flank skin, and in LNs draining these sites (Fig. 2, A and B). These data demonstrated a relative enrichment of LC at the tumor site 7 days after injection, at which point the tumors were small but detectable. However, by days 10 and 14 after injection, there was no difference in LC frequency across the skin sites. Tracking the accumulation of migrating LCs in the draining LNs demonstrated a delay in migration (Fig. 2B); an increase in the frequency of LN LCs was observed in some mice 10 days after injection but this was not consistent until day 14 when the tumors were large, and most mice had to be euthanized (fig. S2A). Enhanced expression of Ki67 in LCs in melanoma-adjacent skin at day 7 (fig. S2B) suggests that accumulation of LCs at this point was due to enhanced proliferation of LCs rather than recruitment to the site of the tumor. Therefore, to understand the molecular mechanism underlying the delayed migration of LCs proximal to tumors, we developed an executable model of LC regulation.

### An executable network models LC behavior

To model LCs, we built an executable qualitative network model [(*33*); Fig. 2C] using the BioModelAnalyzer (BMA) tool (https://biomodelanalyzer.org). The model is an executable computer program describing the core pathways linking driver mutations and transcriptional states to observable cell behaviors. We reasoned that the ability of LCs to activate immune surveillance against cutaneous melanomas would depend on three key outputs: residency of LCs within the epidermis and by correlation activation of migration to draining LNs, survival of LCs, and proliferation of LCs. It is established that LCs migrate in response to IL-1β (*34*), but this is thought to depend on IL-1R signaling in KCs (*35*). Therefore, the model makes clear the dependence of the IL-1β pathway on KC signaling. The LC model consists of 38 nodes and 46 edges (either activatory or inhibitory; table S1). Each node can occupy discrete levels of activity, with each node in the model having three possible states (0 to 2). The level of activity at any discrete time point is determined by the activity of its regulators, as described mathematically by a target function (table S2). A detailed description of the model and its components is given in Supplementary Text. We validated the model against a set of 17 experiments from the literature (table S3); the model correctly predicted 100% of 21 experimental measurements from these 17 experiments.

### Delayed LC migration to LNs is explained by melanoma-derived TNF-α

**Fig. 3.**
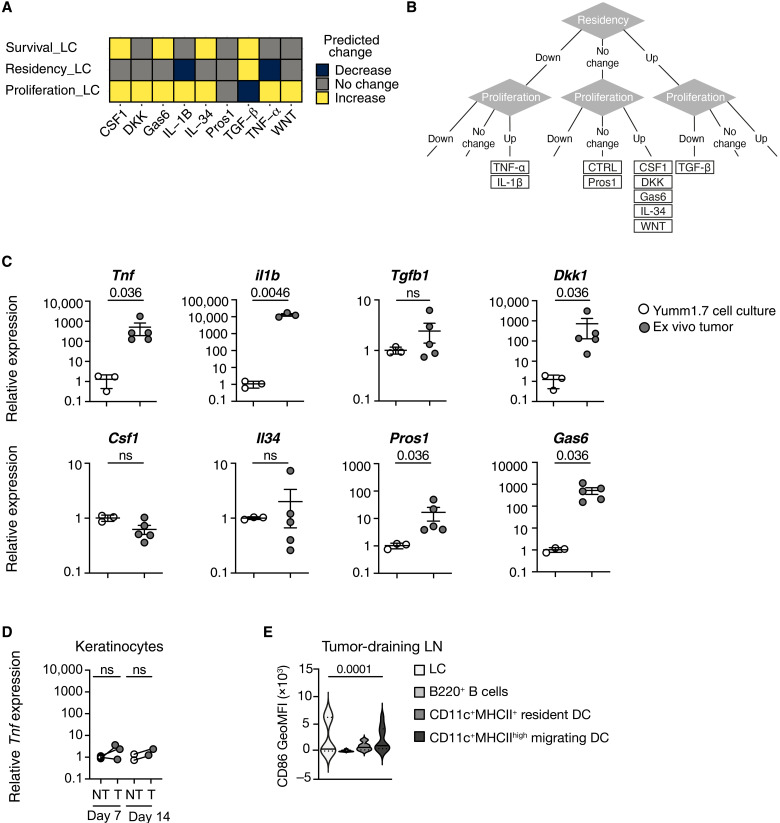
TNF-α expression is associated with LC migration. (**A**) The heatmap shows the predicted behavior of LCs based on different hypotheses for the molecular mechanism of melanoma control of LCs as predicted by the LC network model. Proteins listed in the columns are up-regulated, except for DKK, which is down-regulated. (**B**) Flow chart demonstrating how experimental observations of LC residency and proliferation determine a consistent molecular hypothesis. (**C**) Line graphs show the relative expression (compared to β-actin) of selected genes by YUMM1.7 melanoma cells in culture, or from engrafted YUMM1.7 tumors harvested at day 14. Circles show individual experimental samples (white, in vitro cultures, *N* = 3; gray, ex vivo tumors, *N* = 3 to 5 biological replicates), with bars showing the mean ± SD over all samples. Ex vivo tumor results are pooled from three independent experiments. Data were analyzed using a Mann-Whitney test, and *P* values are indicated on the graphs; ns indicates *P* > 0.05. (**D**) The line graph shows the relative expression (compared to β-actin) of TNF-α by sorted KCs harvested from nontumor (NT) or tumor-adjacent (T) skin epidermis 7 or 14 days after injection. Points show individual mice; data are from pooled mice from two independent experiments (day 7, *N* = 3; day 14, *N* = 2); data were analyzed using a Wilcoxon matched pairs signed-rank test, ns = not significant. (**E**) Violin plots show the expression of CD86 (geometric mean fluorescent intensity) on gated LN LC, B220^+^ B cells, CD11c^+^MHCII^+^ resident DC, and CD11c^+^MHCII^high^ migrating DC draining day 14 established melanomas. Plots show median (solid line) with quartiles (dotted lines). Data are pooled from three independent experiments; *N* = 9 per group. Matched samples were analyzed using a Friedman (one-way ANOVA) nonparametric test; multiple comparisons show no significant difference between LC, resident DC, and migrating DC, but LC versus B cells. *P* = 0.011.

The LC model contains nine soluble signaling molecules, many of which have been linked to melanoma biology: TNF-α (*36*), TGF-β (*37*), IL-1β (*38*), canonical WNT ligands and the soluble frizzled inhibitor DKK (*39*), ligands of the CSF1 receptor CSF1 (*40*) and IL-34 (*41*), and Tyro3, Axl, and MerTK (TAM) receptor ligands Pros1 and Gas6 (*42*). Therefore, we used changes in expression of these factors as the basis for different hypotheses to predict changes in LC behavior in the presence of melanomas in our syngeneic mouse model (Fig. 3A). Given that no loss of LCs, indicating changes in survival, was observed in vivo (Fig. 2A) and there was no predicted decrease in LC survival in our computational model, we focused our attention on proliferation and residency. This analysis demonstrated that hypotheses involving changes in TGF-β, IL-1β, and TNF-α altered the residency behavior of LCs, while changes in the other factors specifically affected proliferation. Each of the hypotheses can be categorized on the basis of the proliferative and residency behavior predicted by the modeling (Fig. 3B). Comparing the experimental measurements from the mouse model to the computational model predictions demonstrated that CSF1, Gas6, IL-34, or WNT up-regulation or DKK down-regulation by melanoma cells could explain increased LC proliferation without affecting residency behavior at day 7, although by day 14 LC numbers had reduced to homeostatic levels (Fig. 2A). By comparison, decreased residency of LCs at day 14 could only be explained by up-regulation of TNF-α or IL-1β (Fig. 3B), and we focused on this response.

To link our computational hypotheses to in vivo biology, we compared gene expression from day 14 murine intradermal tumors to in vitro cultured YUMM1.7 cells (Fig. 3C). Expression of *Tnf*, *Il1b*, *Pros1*, and *Gas6* was enhanced upon tumor growth in vivo, as was *Dkk1*, in contrast to studies showing down-regulation of *DKK* genes in melanoma cell lines in vitro, relative to melanocytes (*39*). Levels of *Wnt3a* mRNA were below detection threshold, and no changes in the other genes were found (Fig. 3C). In healthy skin, KCs are the major source of TNF-α, which is produced upon IL-1β–dependent signaling (*35*) and which stimulates migration of immunogenic LC (*11*). Therefore, we questioned whether melanoma cells could become the dominant source of TNF-α in the skin or whether indirect activation of KCs in tumor-bearing skin, for example, via melanoma IL-1β, would also contribute to TNF-α–dependent migration of LCs. To address this question, we analyzed TNF-α production from KCs isolated from skin adjacent to small (day 7) or large (day 14) tumors compared to cells isolated from the contralateral flank (Fig. 3D and fig. S3). These data demonstrated a minor response to melanoma growth by KCs, and levels of *Tnfa* were notably lower than in melanoma cells (Fig. 3C). Thus, our functional and observational analysis converged on the up-regulation of TNF-α as a mechanism of LC migration in large tumors. Since TNF-α induces migration of LC to LN, we investigated whether melanoma-activated LC had the potential to stimulate naïve T cells upon migration to tumor-draining LN in our murine model and analyzed expression of the costimulatory molecule CD86 on LC compared to other resident (B cells, CD11c^+^MHCII^+^ DC) and migrating (CD11c^+^MHCII^high^ DC) antigen-presenting cells. Figure 3E shows that expression of CD86 varied between mice, but that some LC and migrating DC expressed higher levels of CD86 than LN resident cells. Together, our findings are consistent with the hypothesis that melanoma growth in the skin does not trigger KC-dependent mechanisms of LC activation, but rather that melanoma-derived TNF-α may induce LC migration once large primary tumors have developed.

### An integrated melanoma-LC model captures the impact of melanoma-derived TNF-α on LCs

Our data suggested that LCs were ignorant to melanoma growth until tumors reached a critical mass. Therefore, to determine the cellular mechanisms by which this may occur and further characterize the relationship between melanoma and epidermal LCs, we developed an integrated executable model based on melanoma-derived TNF-α. First, we built a melanoma model linking driver mutations and transcriptional states to observable cell behaviors. We chose to model *BRAF*^V600E^ and *NRAS*-mutant tumors because these mutations are dominant in a substantial fraction of cutaneous primary melanomas (*43*) and are well represented in preclinical models. We aimed for the model to account for the main drivers of disease and how these drivers predict the response (in terms of proliferation and apoptosis of tumor cells) to a variety of external stimuli and targeted therapies. This model consists of 89 nodes linked by 162 regulatory edges (tables S4 and S5). As with the LC model, each node can occupy discrete levels of activity, but in this case, each node in the model has a minimum of 2 and maximum of 7 possible states. A detailed description of the model and its components is given in Supplementary Text. We tested the model against a set of 135 experiments from the literature (table S6). While we model the impact of the differentiation status, as controlled by the level of *MITF* expression [from 0 to 3; (*44*)], regulation of this state is considered outside of the scope of the model. The model successfully predicted the outcome of 176 of 198 (89%) experimental measurements from the 135 literature experiments. A full description of how and why the model fails to represent the remaining 11% of experimental measurements is provided in Supplementary Text.

**Fig. 4.**
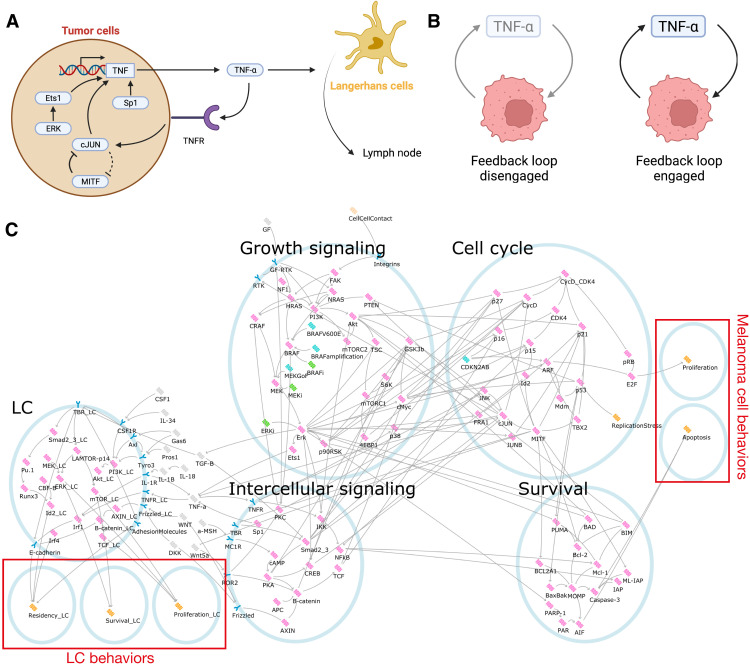
An autocrine TNF-α signaling loop underlies detection of melanoma by LCs. (**A**) Schematic showing the model for a molecular mechanism of autocrine TNF-α signaling in melanoma. (**B**) The positive feedback loop formed by autocrine TNF-α leads to bistability. (**C**) Executable model of melanoma-LC signaling as seen in the BMA tool. Pink nodes represent proteins or complexes, orange nodes represent processes or phenotypes, cyan nodes represent genes or genetic modifications, gray nodes represent secreted proteins, and blue nodes represent receptors. Activating interactions are represented by arrows (→), and inactivating interactions are represented by bars (⟞). The _LC suffix is used to distinguish proteins that are also present in the melanoma model.

There is limited research into the mechanisms of TNF-α production in melanoma cells by stromal cells (*45*), and measurements in cell culture have found variability in TNF-α production between cell lines (*36*). Regulation of the *TNF* gene is complex [reviewed in (*46*)] but is known to depend on a set of transcription factors, including the NFAT family, Ets-1, Elk1, ATF2, cJUN, and Sp1. Ets-1 is a known downstream target of the MAPK pathway (*47*), and cJUN is known to be dysregulated in melanoma (*48*, *49*). Inhibition of Sp1 with decoy oligodeoxynucleotides was shown to decrease in vivo expression of *Tnf* in B16-F10 melanoma cells (*50*). Therefore, we pursued a mechanism of *TNF* expression in melanoma based on Sp1, Ets-1, and cJUN activity (Fig. 4A). TNF-α is known to induce its own production (*51*), leading to an autocrine signaling loop. These positive feedback loops are associated with bistability in biological systems (*52*), meaning that the system can occupy one of two possible states. In this case, we would expect there to exist conditions under which melanoma cells can maintain TNF-α production through autocrine signaling, or not, depending on whether the feedback loop is engaged (Fig. 4B). With this in mind, we added the mechanisms of TNF-α production to the melanoma model and integrated it with the LC model to create a melanoma-LC model (Fig. 4C and tables S7 and S8).

We first confirmed that the melanoma-LC model replicated the same experiments that each of the individual models did (table S9); the melanoma-LC model replicated the same set of experiments as the individual models, except for five cases, where the model deviated numerically from the specification; further details are described in Supplementary Text. For a typical *BRAF*^V600E^-driven tumor, the model exhibits two steady states, one with the TNF-α feedback loop engaged and the other with it turned off, as expected. This does not depend on loss of *PTEN*, but transition into the *MITF*^low^ transcriptional state locks the model into the state producing TNF-α.

### In silico screening identifies key mutations within different melanoma mutational backgrounds that inhibit LC migration

**Fig. 5.**
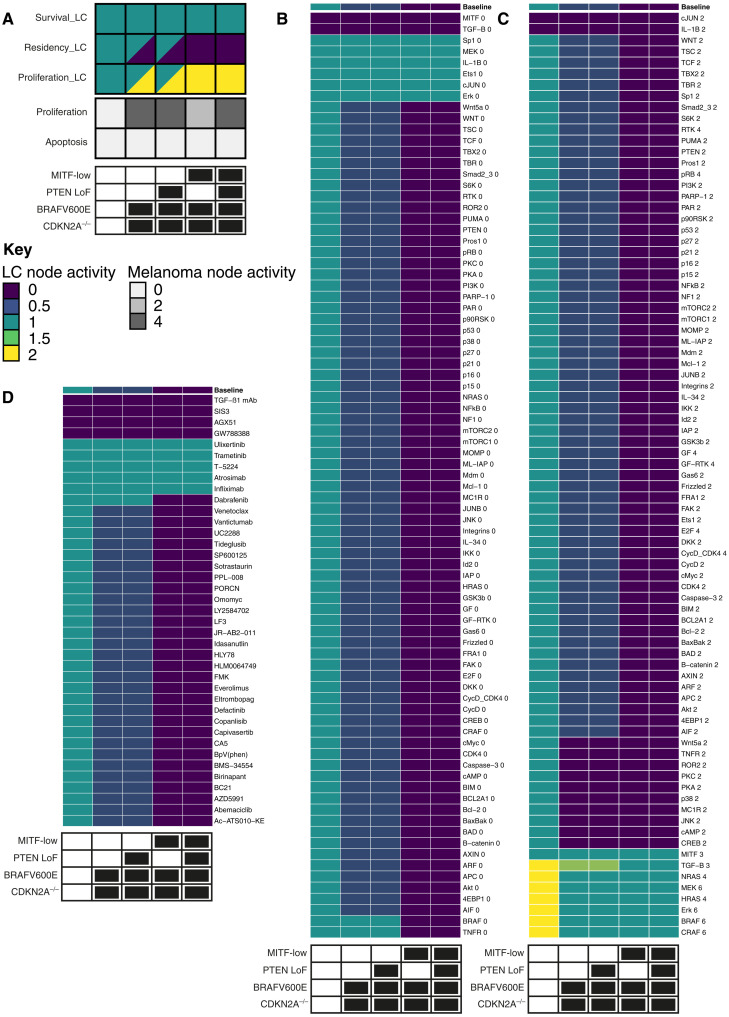
In silico screening identifies key mutations that inhibit LC migration. (**A**) Heatmap showing baseline, unperturbed levels of each behavior node. Multicolored squares indicate values of constraints (maximum and minimum) identified for each given node. The color of each cell corresponds to the level of the respective node. All data are shown in four melanoma backgrounds and a healthy skin control, indicated with black and white panels below each plot. (**B** to **D**) Heatmaps show effect on predicted LC residency of loss-of-function mutations, gain-of-function mutations, and targeted therapies. In (B) and (C), the number next to a node represents the value to which it is set in the perturbation.

The executable model of melanoma-LC interactions predicted that melanoma cells occupying transcriptional states with high TNF-α expression induced LC migration from the melanoma-adjacent epidermis (Fig. 5A). We reasoned that this would create a potential selection pressure to lose TNF-α expression to avoid initiation of the anti-tumor T cell response. Therefore, to test this, and to identify loss-of-function mutations that block LC migration, we used in silico screening to systematically investigate the impact of melanoma mutations on LC residency in four melanoma backgrounds driven by *BRAF*^V600E^ (table S10 and Fig. 5, B and C). In *MITF*^high^ backgrounds, regardless of *PTEN* mutation, the model can occupy one of two states (Fig. 4B)—one in which TNF-α is produced and LC migration is induced and another in which LCs remain resident in the epidermis. In both *MITF*^low^ backgrounds, the TNF-α loop is always engaged, leading to constitutive TNF-α production and reduced LC residency. Therefore, we sought mutations that could enhance LC residency in all of these backgrounds, potentially offering the melanoma cells a selective advantage.

Focusing on the loss-of-function mutations (Fig. 5B), a loss of cJUN, Sp1, and Ets-1 function, which are key to TNF-α production in the model, promoted residency of LCs irrespective of the genetic background of the melanomas, while loss of signaling via the TNF receptor enhanced residency only in *MITF*^high^ backgrounds. These data suggested that while TNF-α production by melanomas has a fundamental influence on LC behavior, reinforcement of the feed-forward loop via TNF-α signaling within melanomas is more highly dependent on the tumor genetics. Loss-of-function mutations in *BRAF*, *MEK*, or *ERK* could lead to loss of TNF-α and enhanced LC residency in the model, but these mutations are highly unlikely to offer a selective advantage in real tumors due to their detrimental effects on cell proliferation and apoptosis (figs. S4A and S5A). Likewise, MAPK pathway gain-of-function mutations that enhanced LC residency would also be associated with poor tumor growth since extremely high levels of MAPK signaling are detrimental to cells (figs. S4B and S5B) (*53*, *54*). *MITF* up-regulation, which is associated with reduced proliferation levels (fig. S5B) (*44*), is evident in tumors immediately following treatment with BRAF inhibitors (*55*, *56*) and was predicted to enforce LC residency in all backgrounds (Fig. 5C). Consistent with the biological role of TGF-β in controlling LC residency within the epidermis, our analysis demonstrated that loss of TGF-β was associated with a decrease in LC residency in healthy skin and in the *MITF*^high^ state (Fig. 5B), while mutations that directly promoted TGF-β gain of function strongly or indirectly led to TGF-β up-regulation (for example, MAPK gain-of-function mutants) retained LCs in healthy skin and across the melanoma backgrounds (Fig. 5C). We noted that loss of IL-1β expression could also enhance LC residency across the melanoma backgrounds, supporting the potential TNF-α–independent activation of LC migration by IL-1β (*57*). It was notable that the gain in function of a panel of genes including those coding Wnt5a, ROR2, PKC, PKA, p38, and JNK promotes LC migration in the *MITF*^high^ state (Fig. 5C). This mechanism occurs via activation of cJUN, leading to stabilization of the TNF-α feedback loop. As cJUN activity defines the *MITF*^low^ transcriptome (*48*), factors such as Wnt5a, which can activate cJUN, are associated with the dedifferentiated melanoma state (*58*). This reinforces the view that the *MITF*^low^ state is associated with the TNF-α feedback loop. Therefore, together, these data demonstrate that cutaneous melanomas may acquire mutations that limit LC migration to LNs without affecting fitness and survival of the growing tumors.

### Computational analysis predicts combinatorial melanoma treatments that preserve LC function in the skin

Targeted therapies are focused on disabling melanoma growth, but there is little understanding of how treatments may affect the establishment of immune surveillance. Optimal therapeutic approaches would disarm tumors while augmenting migration of tumor antigen–bearing LCs to T cells in draining LNs. Therefore, having defined how melanoma mutagenesis affects LC behavior, we next investigated the impact of targeted therapies on LC residency by repeating our in silico screens on the melanoma-LC model using a list of targeted therapies known to interact with proteins represented in the model (table S11 and Fig. 5D). We looked for therapies that would enhance LC migration across all transcriptional and genetic backgrounds. We identified three drugs targeting TGF-β signaling: TGF-β1 monoclonal antibodies, SIS3 and GW788388 (*59*), and the Id2 inhibitor AGX51 (*60*). Id2 acts downstream of TGF-β signaling in LCs (*61*) and is a direct inhibitor of Residency_LC in the model. Inhibitors of TGF-β signaling act directly on LCs, which migrate in response to loss of TGF-β signaling (*62*). However, these effects are nonspecific and have no activity against melanoma growth or survival (figs. S4C and S5C), and the drugs are predicted to affect LCs in healthy tissue as well. We also tested whether combinations of two drugs could affect LC migration in the model but found that no combination, except combinations including drugs that were effective individually, was effective (fig. S6). Therefore, TGF-β signaling inhibitors may be a therapeutic avenue to enhancing LC immune control of primary melanomas.

**Fig. 6.**
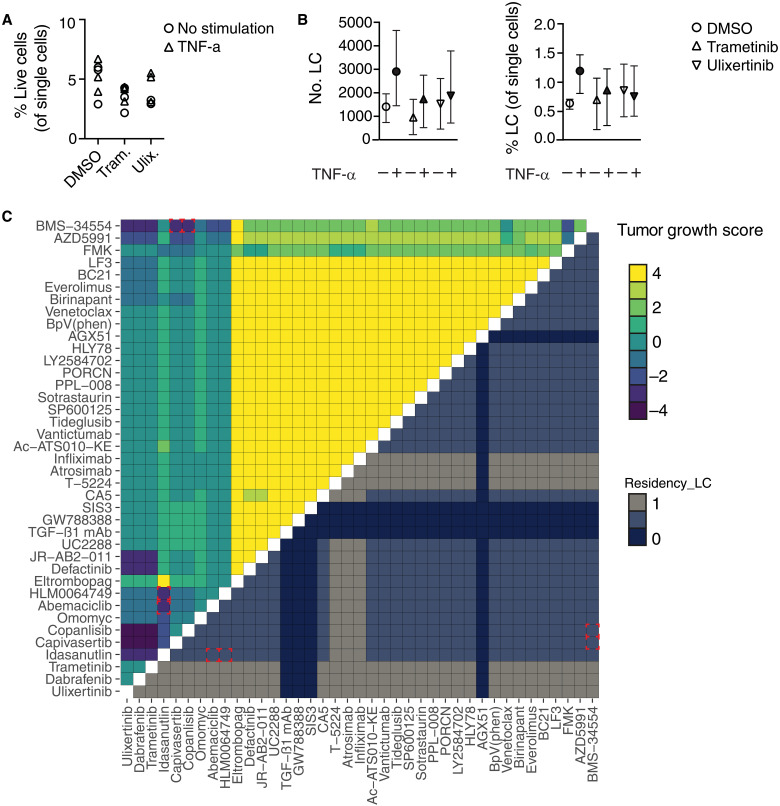
Combination screening predicts combinatorial melanoma treatments that preserve LC function in the skin. (**A** and **B**) Summary graphs show the impact of MAPK inhibitors on TNF-α–induced LC migration. (A) Summary graph showing viability (% live cells) in the different culture conditions. Tram., trametinib (0.1 μM); Ulix., ulixertinib (0.5 μM). Symbols represent individual samples from three pooled experiments without (circles) or with (triangles) TNF-α stimulation. (B) Summary graphs showing total number (left) and frequency (right) of LCs emigrating out of epidermal sheets in the presence or absence of TNF-α with DMSO (circles), trametinib (upright triangles), or ulixertinib (downward triangles). Symbols show mean ± range of three independent experiments. There were no significant differences between paired groups using a Wilcoxon nonparametric paired test. (**C**) Top, heatmap showing predicted tumor growth score (proliferation − apoptosis); bottom, heatmap showing the level of the Residency_LC node. Red squares highlight optimal combinations. Data are shown in the *BRAF^V600E^ CDKN2A^−/−^ PTEN^−/−^ MITF^high^* background. Drug targets are listed in table S11.

Our analyses revealed that the MAPK pathway inhibitors dabrafenib (mutant BRAF inhibitor), trametinib (MEK), and ulixertinib (ERK) reduce LC migration in *MITF*^high^ backgrounds. Note that vemurafenib, another mutant BRAF inhibitor commonly used to treat melanoma, would be modeled identically to dabrafenib; in this analysis, these drugs could be used interchangeably. All MAPK inhibitors enhance LC residency because the MAPK pathway is required for Ets-1 activity and, consequently, for *TNF* expression in melanoma in the model. Furthermore, trametinib and ulixertinib inhibit the MAPK pathway in LCs, which reduce both LC survival and proliferation (fig. S7). To test these predictions, we used an ex vivo LC migration assay in which the direct impact of trametinib and ulixertinib on TNF-α–induced LC migration could be analyzed in the absence of the additional impact of these drugs on tumor growth. Thus, murine epidermal sheets were cocultured for 48 hours with TNF-α with or without targeted drugs at concentrations known to inhibit MAPK signaling pathway components (*63*). We did not observe a difference in viability of LCs between groups in these short-term cultures (Fig. 6A). However, while TNF-α activated increased migration of LCs into the culture medium, we no longer observed this trend upon addition of trametinib or ulixertinib to the culture medium (Fig. 6B). Therefore, our data suggest that potential off-target effects leading to blockade of immune surveillance due to retention of LCs in the skin should be considered when using MAPK inhibitors.

Our in silico screening analysis demonstrated that none of the currently available treatments would induce LC migration in *MITF*^high^ melanomas, and that some, in fact, prevented egress of LCs out of the skin. Therefore, we extended the search to look for the most effective combination therapies that would not enhance LC residency (Fig. 6C). We identified four combination treatments matching these criteria. The IκB kinase (IKK) inhibitor BMS-34554 (*64*) was identified in combination with the phosphatidylinositol 3-kinase (PI3K) inhibitor copanlisib (*65*) and the Akt inhibitor capiversatib (*66*). However, the predicted survival and proliferation of the LCs under treatment with these factors (figs. S8 and S9) indicated that these drugs may be cytotoxic to LCs, which depend on PI3K/Akt pathway signaling for survival. Alternatively, we identified the MDM2 inhibitor idasanutlin (*67*) in combination with the CDK4 inhibitor abemaciclib (*68*) and E2F inhibitor HLM0064749 (*69*). These combinations did not affect LC migration and were not predicted to have adverse effects on LC survival or proliferation (figs. S8 and S9). Therefore, selection of these treatments may represent effective strategies to combat melanoma without undermining LC-based immune surveillance.

## DISCUSSION

Our understanding of immune interactions in metastatic melanomas has expanded substantially in the past decade, and with this, the use of monoclonal antibodies to reactivate immune detection and destruction of tumors has revolutionized treatments. Despite this, treatment of primary melanomas has remained unchanged. Surgical removal of primary melanomas can be curative, but the success of treatment in preventing reoccurrence of melanomas is highly dependent on the thickness of the malignant lesion; once melanoma cells escape the confines of the skin, the prognosis deteriorates rapidly (*70*). Activating early immune detection of melanomas could improve the treatment of primary thick tumors before metastasis, but this requires a better understanding of how tumors are able to develop undetected within the active immune environment of the skin. LCs are immune sentinels of the skin and reside in the epidermis at the site of melanoma development, but how tumor growth may influence LC behavior remains understudied. Here, we have combined a murine in vivo model of melanoma development with in silico executable modeling of melanoma-LC interactions to investigate whether and how LCs respond to malignancy in the skin. Our experimental data demonstrated that LCs do not respond to early skin tumors, but activation-induced migration to draining LNs is only triggered once melanomas have reached a critical mass. Combining in silico models with in vivo validation of tumor gene expression suggested a mechanism for melanoma immune evasion based on production of TNF-α by the tumor, whereby a positive feedback loop induced by TNF-α signaling allows for small tumors with negligible TNF-α production and large tumors producing significant amounts of the cytokine. In silico screening of the melanoma-LC model suggested that treatment of primary melanomas with MAPK pathway inhibitors could have the unintended consequence of further limiting LC control of the tumor. However, our screen demonstrated that patients may benefit from combinatorial delivery of targeted drugs to block tumor growth without disarming immune surveillance by LCs in the skin.

Our experimental data demonstrated that LCs are not activated upon development of small melanomas; injection of the tumor bolus induced proliferation of LCs in situ without activation and release out of the epidermis. However, LC migration was activated once skin tumors had reached a large critical volume. Analysis of LCs from patient sentinel LNs has demonstrated that they are inefficient at priming naïve T cells (*12*–*14*), and we observed variable levels of CD86 on LC in tumor-draining LN. However, we suggest that even in situations where immunogenic CD86^high^ LCs do encounter T cells, the delay in migration means that anti-tumor T cells are unable to counteract the tumor microenvironment and initiate rejection of the large established tumors. Our current experiments do not determine whether LCs are ignorant of melanoma growth or retained in the skin at early time points. We observed a trend in increase in TGF-β production by some YUMM tumors at day 14, and it has recently been shown that expression of the TGF-β–activating integrin αvβ8 in patients correlates with Breslow depth (*71*). Thus, activation of LC autocrine latent TGF-β by melanoma αvβ8 may contribute to retention of LCs in the skin. These data are consistent with earlier studies in which “progressor” squamous cell carcinomas inhibited LC migration in a TGF-αvβ8–dependent manner, while LC migration was unimpeded by “regressor tumors” (*72*).

We developed an executable LC model based on extensive review of the published literature and generated hypotheses to predict potential melanoma-derived factors that would affect the LC behavior observed in vivo. This analysis demonstrated a clear division of LC signaling between survival and proliferation versus residency. Factors such as the myeloid growth factors CSF1 and IL-34 and TAM receptor ligands Pros1 and Gas6 were important for LC survival and proliferation in situ, while the analysis confirmed the dominant migration-activating roles of the proinflammatory cytokines IL-1β and TNF-α, with enhanced residency conferred by TGF-β. To test the biological significance of our predictions, we compared gene expression between in vitro cultured YUMM1.7 cells and ex vivo large tumors 14 days after injection. This analysis demonstrated relatively high levels of *Tnf* and *Il1b* expression within the skin, supporting the observed migration of LCs at this time point. We further showed that at this point, the melanomas were the dominant source of TNF-α rather than KCs surrounding the tumor area. LC growth factors were not expressed at high levels by in vivo tumors; however, we observed significant increases in *Pros1* and *Gas6*; both TAM receptor ligands have previously been associated with the suppression of anti-tumor immunity (*73*, *74*), and Pros1 is known to inhibit differentiation of bone marrow–derived LCs (*75*), suggesting that growing melanomas may directly impair LC function in situ before migration.

**Fig. 7.**
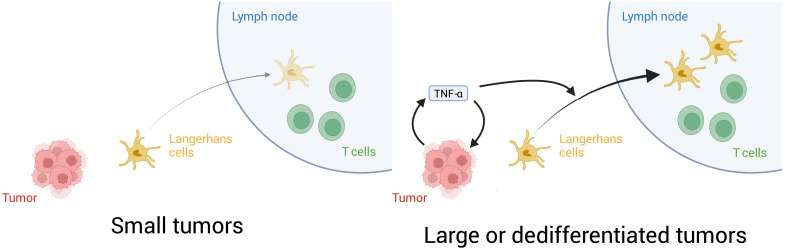
Schematic summarizing the findings from this study. Diagram shows the molecular mechanisms suggested by our data. In small tumors, TNF-α does not accumulate to sufficient levels to maintain an autocrine signaling loop in the tumor. In larger or dedifferentiated tumors, increased levels of TNF-α accumulate to engage the autocrine feedback loop and maintain high levels. Increased TNF-α triggers migration to LNs. However, patient data suggest that at this point LCs are dysfunctional and immune rejection of established melanomas does not occur.

To determine the molecular mechanisms by which melanomas impaired LC function, we generated a model of primary cutaneous melanoma and integrated this with our LC model. The transition from benign nevi to in situ melanomas has been well described (*76*, *77*), occurring through mutations to the MAPK signaling pathway, especially *BRAF*^V600E^ (*43*), and other tumor suppressor genes such as *CDKN2A* and *PTEN* (*78*). Such mutations may occur as a result of exposure to UV mutagenesis, while certain inherited variants of genes, such as *CDKN2A*, can also predispose individuals to melanoma (*79*). Together, these genetic changes result in proliferation and transformation of neoplastic nevi that may ultimately acquire the potential to break through the epidermal basement membrane and enter the circulation via the dermal lymphatics. We modeled *BRAF*^V600E^ and *NRAS*-mutant tumors, which form most patient cutaneous primary melanomas in the clinic (*43*), and considered a range of further molecular alterations, namely, *MITF* status, loss of *PTEN*, presence of *BRAF*^V600E^, and loss of *CDKN2A*. Reasoning that migration of LCs to draining LNs was central to the role of LCs in activating T cell responses, and thereby immune surveillance against cutaneous melanomas, our computational analyses focused on the factors that would alter LC residency in the epidermis. This approach identified melanoma-derived TNF-α as the major factor driving LC migration once tumors had reached a critical mass. Mechanistically, we predicted that a model of melanoma-LC interaction based on TNF-α production by melanoma cells exhibited bistability in *MITF*^high^ backgrounds. This is consistent with a paradigm where small tumors, or tumor cells in vitro, occupy a state devoid of TNF-α (Fig. 7). The development of a large tumor coincides with occupation of a state showing notable production of TNF-α. This could arise as a result of accumulation of the cytokine, which may be produced by melanoma cells at low level, or as a result of localized inflammation driven by infiltrating immune cells leading to widespread TNF-α production in the rest of the tumor. Our computational model also predicts that dedifferentiated *MITF*^low^ cells consistently occupy a state with high TNF-α production. Thus, our executable model encapsulates a hypothesis for the mechanism of immune evasion during development of primary melanoma.

The push toward precision oncology has created a need for techniques that can account for the unique nature of each patient’s cancer. Unlike costly and time-consuming patient-derived xenograft and organoid models, personalizable computational models offer a cost-effective alternative. If such computational models are to account for the impact of the immune system on effective therapy, then representations of stromal and immune components of tumors as well as cancerous cells will be required. A key strength of our computational approach is that, by modeling both tumor and immune cells, we can account for the impact of perturbations to both cell types. Computational modeling of KCs and melanocytes has proven an informative strategy to probe the impact of UVB radiation on melanogenesis (*80*), but here, we apply mechanistic, pathway-based modeling to melanoma and immune cells. Using our integrated executable LC-melanoma model, we screened the negative impact of targeted drugs that would affect melanoma signaling pathways and demonstrated that the MAPK pathway inhibitors dabrafenib (mutant BRAF inhibitor), trametinib (MEK), and ulixertinib (ERK) would be detrimental to immune surveillance by reducing LC migration to LNs. Consistent with this prediction, addition of trametinib and ulixertinib to LC emigration cultures reduced TNF-α–induced migration out of the epidermis. By controlling for this unwanted effect, we subsequently predicted effective drug combinations that did not limit LC migration and could also determine that LC survival was not impaired by these treatments. As our computational models are based on literature-derived networks, all of the effects identified are interpretable, meaning that each drug applied can be linked to a specific molecular mechanism. This is in contrast with black box machine learning approaches (*81*, *82*), which often cannot be linked to specific molecular mechanisms. While in this work we account solely for melanoma cells and LCs, future computational models may integrate other immune cells such as T cells. By integrating further cell types into the computational model, a more holistic representation of tumors could be generated, allowing for further characterization of off-target effects in primary melanoma.

Our computational modeling approach allows us to explore the molecular mechanisms behind the observed phenomena, but as with all models, they are dependent on the data analysis that underpins them. In executable models, this data analysis leads to the definition of edges in a network and target functions that determine its dynamic behavior. In this study, we built the model manually from information available in the literature (see tables S1, S4, and S7 for sources). While building models automatically from structured datasets, such as the STRING database, is a promising alternative to manual analysis, existing databases suffer from lack of cell type specificity and often do not include the signed, directed interactions needed for executable modeling (*83*). On the basis of our data analysis, our model explains melanoma cell apoptosis and proliferation and LC apoptosis, proliferation, and migration. Other behaviors, such as activation of LCs, are presently not included, and therefore, the model cannot predict how activation of LCs will be affected by perturbations, although it could be expanded in the future to explore these additional cell behaviors. In this study, we have implemented the model with the BMA tool, which uses a synchronous update scheme. Some other modeling techniques use asynchronous schemes, which can be more accurate representations of biological systems where chemical reactions do not occur at uniform speeds, or where a small number of molecules lead to stochastic decisions that are better captured by the nondeterminism inherent in asynchrony. However, for large networks with a low range of values for nodes, such as ours, the difference between synchronous and asynchronous models is small, and asynchronous computation may exaggerate small differences (*33*). Furthermore, to be able to profile many potential combination treatments requires an analysis method that scales to large networks, and asynchronous stable states are more difficult to compute (*84*). We previously demonstrated the utility of asynchronous update schemes in other modeling systems (*85*–*87*). Last, the unified model makes predictions relating to phenomena occurring over varying time scales, from phosphorylation events on the order of milliseconds to cell migrations occurring over hours. Although the model makes predictions about the behavior of cells on the longer time scale of migration, the model itself only aims to predict the establishment of cellular states, including those initiating migration. Therefore, the model only deals with reactions occurring on the shorter time scale of minutes.

In this study, we have explored one facet of cutaneous immune surveillance of melanomas—the early detection of growing tumors by LCs—using a unique synthesis of computational modeling and experimental validation to deepen our understanding in a way that could not be achieved by experiment alone. Our results add to an increasing appreciation of the complex immune networks, including conventional and regulatory T cells and other macrophage populations that are co-opted by melanomas to avoid immune detection and facilitate spread beyond the skin. Developing our understanding of the mechanisms of immune surveillance by LCs and other immune cells could lead to development of strategies to unleash immune control of melanoma in the skin.

## MATERIALS AND METHODS

### Murine model of cutaneous melanoma

Female C57BL/6 (B6) mice (6 to 8 weeks old) were purchased from Charles River. Mice were housed in specific pathogen–free conditions. All procedures were conducted in accordance with the UK Home Office Animals (Scientific Procedure) Act of 1986 and were approved by the UCL Animal Welfare and Ethical Review Body (PP4506002). Murine B6 YUMM1.7 tumor cells [American Type Culture Collection (ATCC) CRL-3362] were grown in Dulbecco’s modified Eagle’s medium (DMEM)–F12, Hepes (Thermo Fisher Scientific, no. 11330032) supplemented with 10% heat-inactivated fetal bovine serum (iFBS, Merck, F7524-500ML), β-mercaptoethanol, 1% penicillin, streptomycin, l-glutamine, and nonessential minimal essential medium (MEM) amino acids (all Thermo Fisher Scientific or Sigma-Aldrich) according to ATCC recommendations. Cells were harvested with 0.05% EDTA (Thermo Fisher Scientific, no. 25300062) and counted before injection. Mice received 5 × 10^5^ tumor cells injected intradermally into the shaved flank. Recipients were cohoused where possible.

### Generation of tissue single-cell suspensions

#### Back skin

Approximately 1-cm^2^ areas of skin were removed from either adjacent to the tumor site or the contralateral flank. The skin was floated on 3 ml of Hanks balanced salt solution (HBSS) (Thermo Fisher Scientific) 2% iFBS with 1 ml of dispase II (10 mg/ml; Thermo Fisher Scientific, catalog no. 04942078001) overnight at 4°C. The epidermis was peeled off, mechanically chopped up into small pieces, and vortexed in HBSS/10% iFBS. Epidermal cells were filtered through 70- and 40-μm cell strainers (Greiner, Thermo Fisher Scientific, nos. 542040 and 542070) before centrifugation and resuspension in fluorescence-activated cell sorting (FACS) buffer [1% iFBS/1 mM EDTA (Thermo Fisher Scientific, no. E7889-100ML) in phosphate-buffered saline (PBS)].

#### Lymph nodes

Draining LNs were mechanically disrupted in FACS buffer using a syringe plunger. The cells were filtered through a 40-μm cell strainer and resuspended in FACS buffer.

#### Tumor cells

Tumors were isolated with care to remove all attached skin. The tissue was manually chopped into small fragments and incubated at 37°C for 1 to 2 hours in digestion buffer [Liberase (25 μg/ml; Sigma-Aldrich Roche, 5401119001) and deoxyribonuclease (250 μg/ml; Thermo Fisher Scientific, Roche, catalog no. 10104159001) in 1× DNA buffer (1.21 tris base, 0.5 g of MgCl_2_, and 0.073 g of CaCl_2_) in 1 ml of PBS]. Digested tissue was transferred into C tubes (Miltenyi Biotec, no. 130-093-237) containing RPMI 1640 (Thermo Fisher Scientific) and 10% iFBS and physically disrupted using the GentleMACS tissue dissociator (Miltenyi Biotec). Cells were filtered through a 40-μm cell strainer and resuspended in Qiagen buffer RLT (Qiagen, no. 74004) containing 1% β-mercaptoethanol for isolation of RNA.

### Flow cytometry and cell sorting

Epidermal and LN cells were distributed in 96-well V-bottom plates and incubated in 2.4G2 hybridoma supernatant (containing αCD16/32) for at least 10 min at 4°C to block Fc receptors. For cell surface labeling, cells were incubated with fluorochrome-conjugated antibodies diluted in 100 μl of FACS buffer (PBS/1 mM EDTA/1% iFBS) at 4°C for at least 20 min in the dark. To detect LCs, the same flow panel was used for both epidermal and LN cell suspensions: anti–B220-BV786 (clone RA3-6B2 BD, Biosciences, no. 563894, RRID AB_2738472), anti–CD45.2-PerCP-Cy5.5 (clone 104, eBioscience, no. 45-0454-82, RRID AB_953590), anti–CD11b-efluor450 (eBioscience, no. 48-0112-82, RRID AB_1582236), anti–MHCII-APC-Cy7/780 (clone M5/114, eBioscience, no. 47-5321-82, RRID AB_1548783), anti-CD24-BV650 (clone M1/69, BD Horizon, no. 563545, RRID AB_2738271), and anti–EpCAM-APC (clone G8.8, eBioscience, no. 17-5791-82 RRID AB_2716944). Live cells were identified by exclusion of propidium iodide. LCs and KCs were sorted from epidermal single-cell suspensions using an equivalent flow cytometry panel as for isolation of LCs (fig. S3). Multicolor flow cytometry data were acquired with a BD LSRFortessa X20 analyzer equipped with BD FACSDiva software, and cells were sorted directly into RLT buffer (Qiagen, no. 74004) containing 1% β-mercaptoethanol using BD FACSAria Fusion. Flow cytometry data were analyzed with FlowJo v10 (LLC, USA), and live cells were pregated on singlets (FSC-A versus FSC-H) and a morphological FSC/SSC gate.

### Quantitative reverse transcription PCR

RNA was isolated using the Qiagen Micro RNeasy Kit (no. 74004). RNA was reverse-transcribed to cDNA with a High-Capacity RNA Reverse Transcription kit (Thermo Fisher Scientific, Life Technologies, no. 4368814). Quantitative reverse transcription polymerase chain reaction (qRT-PCR) was performed on the QuantStudio 5 Real-Time PCR System (Thermo Fisher Scientific) using SYBR Green (MAXIMA SYBER GREEN/ROX qpcr 2X VWR, no. K0221). Primers were synthesized by Thermo Fisher Scientific and are listed on table S12. Raw data for each gene were generated in the form of cycle threshold (Ct) values, and gene expression was calculated relative to β-actin using the 2^−ΔΔCT^ method.

### LC migration assay

Epidermis was separated from ear skin as described for back skin above, and the epidermis from one ear per well floated on 1 ml of completed medium (RPMI 1640, Lonza, Switzerland), 5% iFBS, 1% l-glutamine (2 mM), 1% penicillin-streptomycin (100 U/ml), and 50 μM β-mercaptoethanol (all from Thermo Fisher Scientific or Sigma-Aldrich, UK) in 24-well plates. Wells were supplemented with recombinant murine granulocyte-macrophage colony-stimulating factor (GM-CSF) (20 ng/ml) and TNF-α (25 ng/ml) (Thermo Fisher Scientific, PeproTech, nos. 315-03 and 315-01A). Cultures also received dimethyl sulfoxide (DMSO) vehicle, 0.1 μM trametinib, or 0.5 μM ulixertinib every 24 hours. After 48 hours, the medium was harvested and LC numbers were determined by flow cytometry using our LC flow cytometry panel and CountBright Absolute Counting Beads (Thermo Fisher Scientific, no. C36995), as per the manufacturer’s instructions. Exclusion of propidium iodide was used to determine viability. Trametinib (GSK1120212) (871700-17-3) was purchased from Generon, and ulixertinib (S7854) was purchased from SelleckChem. Drugs were dissolved in DMSO and stored at −20°C.

### Experimental study design and statistics

The study was designed according to ARRIVE guidelines. Sample sizes were based on previous experiments. No outliers were excluded, and the number of replicates and independent experiments is given in each figure. Samples analyzed by flow cytometry were excluded when technical errors resulted in an absence of cells being recorded. There was no randomization or blinding. Statistical analysis was performed, and the graphs were generated using GraphPad Prism version 9. Comparison of matched tissue samples from the same mouse was performed using a nonparametric Wilcoxon matched pairs signed-rank test (Figs. 2 and 3), and paired cell types within the same LN using a Friedman [one-way analysis of variance (ANOVA)] nonparametric test (Fig. 3). Gene expression data for Fig. 3 were not normally distributed, and the two samples were compared using a Mann-Whitney test. *P* < 0.05 was considered significant.

### Qualitative networks

We model the networks underlying melanoma and LC behavior as discrete qualitative networks (*33*). Qualitative networks are related to Boolean networks (*88*), but instead of being limited to an ON or OFF state, each node can take any integer value in a finite range. The networks are built using the freely available and open-source (MIT License) BMA tool (https://biomodelanalyzer.org) (*89*). The networks consist of nodes representing proteins, complexes, genes, the action of drugs such as mutant BRAF inhibitors (*90*), and processes such as proliferation. The interactions between nodes are represented as edges (see tables S1, S4, and S7), which can represent either activation [represented by an arrow (→)] or inhibition [represented by a flat head (⟞)]. At any time point in a simulation, each node has an associated level of activity, which can take any value within a range specific to that node. This level of activity is determined by a mathematical function, known as the target function, which takes the level of activity of the nodes regulating the node in question as its input. Unless otherwise specified, the target function is given by avg(*pos*) − avg(*neg*), where *pos* refers to positive regulators (activators) of the node and *neg* refers to negative regulators (inhibitors). When necessary, more complex functions are used to describe behavior such as cases where one input has a dominating effect over another. The target functions used in the models, and their rationale, are described in tables S2, S5, and S8. In cases where nodes with different ranges of activity interact, a scaling factor is used. If node *X* has a range of *a* − *b* and regulates node *X′* with range *a′ − b′*, then it is scaled in the target function of *X′* using the formula

$$\frac{\left(X-a)(b^{\prime }-a^{\prime }\right)}{(b-a)+a^{\prime }}$$

For a given initial state of the network, the model updates synchronously, eventually deterministically reaching a single attractor. When this attractor consists of a single steady state, it is called a fixed-point attractor; attractors with more than one state are referred to as loops. During model testing and analysis, we determine whether the network reaches a single fixed-point attractor from all initial states (*91*). When more than one attractor exists, we call this a bifurcation. When a single fixed-point is identified, the level of the nodes in this state is used as the model’s predictions. When a loop or bifurcation is identified, the midpoint between the limits identified as per Cook *et al*. (*91*) is used. A detailed description of the protocol for network simulation is given by Schaub *et al*. (*33*).

### Model testing

The models were built using an iterative, bottom-up approach. The melanoma and LC models were built using manual curation of experimental data from the literature; the evidence used for each edge is provided in tables S1, S4, and S7, respectively, and an in-depth discussion of the literature underlying the model is provided in the Supplementary Materials. We then gathered a separate list of experiments for each cell type to evaluate the models (tables S3, S6, and S9, respectively). For these experiments, we recorded how changes in genetic backgrounds or perturbations such as targeted therapies (such as BRAF inhibitors) or targeted genetic interventions (such as short hairpin RNAs) affected cellular behaviors. A list of background mutations and known *MITF* status used to simulate specific cell line is provided in table S13. Using these backgrounds, the experimental conditions were reproduced in the model and compared to the cellular behavior described in the literature (the background constraints used for screening can be found in tables S14 to S16). The model was developed through iterative rounds of model development and comparison to the experimental data.

### In silico screening

To identify melanoma mutations with the potential to alter LC signaling, we inactivated (set the target function to equal its minimum value) or activated (set target function to its maximum value) each node in the network individually and in pairwise combinations. We placed bounds on the network stable states under these conditions using the BMA Command Line tool BioCheckConsole and the VMCAI engine and record the impact on each of the melanoma and LC behavior nodes (Fig. 5, A and B). We also screened the network against a list of therapies known to target nodes in the network (table S11) by enforcing the relevant conditions through target functions, as we did for the mutation screen. We report the value of the behavior nodes in either the stable state identified or, if no steady state was found, the midpoint of the bounds place on the node using the algorithm described by Cook *et al.* (*91*) (full dataset given in data S1). The analysis was repeated for four mutational and transcriptional backgrounds and a healthy state control (table S10). Tumor growth score reported in Fig. 6 is calculated as the difference between the proliferation and apoptosis nodes.

### Illustrations

Figures 1, 4 (A and B), and 7 were created using BioRender.com. Plots were generated using R (*92*) with the pheatmap (*93*), RColorBrewer (*94*), ggplotify (*95*), and tidyverse (*96*) packages.

## Supplementary Materials

### This PDF file includes:

Supplementary Text Figs. S1 to S9 Tables S1 to S16 References

### Other Supplementary Material for this manuscript includes the following:

Supplementary Data S1

Supplementary References

## Notes

View/request a protocol for this paper from *Bio-protocol*.
