# Supplementary material for: Localized immune surveillance of primary melanoma in the skin deciphered through executable modeling
Source: Sci Adv. 2023 Apr 12;9(15):eadd1992. doi: 10.1126/sciadv.add1992 (PMC10096595; doi:10.1126/sciadv.add1992)
Supplement: Supplementary file 1 — Supplementary Text Figs. S1 to S9 Tables S1 to S16 References [file sciadv.add1992_sm.pdf]

Science Advances
AAAS

Supplementary Materials for

Localized immune surveillance of primary melanoma in the skin deciphered through executable modeling

Rowan Howell *et al.*

Corresponding author: Jasmin Fisher, jasmin.fisher@ucl.ac.uk; Clare L. Bennett, c.bennett@ucl.ac.uk

Sci. Adv. **9**, eadd1992 (2023)
DOI: 10.1126/sciadv.add1992

The PDF file includes:

Supplementary Text
Figs. S1 to S9
Tables S1 to S16
References

Other Supplementary Material for this manuscript includes the following:

Supplementary Data S1
Supplementary References

Langerhans cell model description

The Langerhans Cell (LC) Model is designed to describe how LCs respond to various cytokines and growth factors. The outputs of the model are Residency, Proliferation and Survival. All nodes in the LC network vary between 0-2. As LCs migrate, proliferate and die at a given rate in the epidermis (*97*), each of the output nodes will have value 1 in the unperturbed state. An increase to level 2 in Residency corresponds to a loss of normal migration while a decrease to level 0 indicates enhanced migration. Note that, where two nodes with the same name (such as MEK) exist in both the LC and melanoma models, the suffix "_LC" has been added to the LC version of the node.

TGF-β is a key cytokine controlling LC behaviour. Loss of TGF-β in the epidermis causes migration of LCs out of the skin, leaving the epidermis devoid of LCs (*62*). TGF-β signalling is mediated by the TGF-β receptor which mainly signals via the Smad2/3 complex and LAMTOR-p14 in LCs. Smad signalling engages activation of Pu.1, RUNX3 and Id2 transcription factors, which is key to maintain residency in the epidermis (*98*). TGF-β also activates the LAMTOR-p14 complex to maintain ERK and mTOR activity in LCs (*99*) for proliferation and survival signalling. Finally, TGF-β signalling contributes to expression of E-cadherin and other adhesion molecules such as EpCAM and Claudin-1 (*100*, *101*).

LCs express the CSF1R, Axl and Tyro3 receptor tyrosine kinases, which all contribute to proliferative and survival signalling through PI3K. CSF1R is a receptor both for CSF1 and IL-34; IL-34 provides vital survival signals for LCs in the epidermis (*102*). Axl is expressed downstream of TGF-β signalling and feedback between Axl and Tyro3 means that Tyro3 is expressed only in the absence of Axl (*103*). The ligands for Tyro3 are Gas6 and Pros1, while Axl accepts only Gas6 (*100*).

LCs respond to Wnt ligands through the canonical Wnt pathway involving the Frizzled receptor and activation of β-catenin (*104*, *105*), contributing to proliferative signalling. β-catenin is also regulated through interaction with E-cadherin (*106*), meaning that E-cadherin down-regulation by TGF-β signalling results in β-catenin activation (*101*). DKK proteins are soluble inhibitors of WNT-Frizzled interactions (*105*) and LCs are also responsive to DKK (*104*).

TNF-α is a key danger signal in the epidermis and LCs respond mainly through the TNFRII receptor (*57*). Keratinocytes produce the inflammatory cytokines IL-1β and TNF-α (in response to IL-1β) (*57*). These two signals induce LC migration (*107*) and converge on the IRF1 transcription factor (*108*). Co-expression of IRF1 with IRF4 is also associated with proinflammatory migratory LCs (*11*). LC migration requires downregulation of E-cadherin (*109*).

Melanoma model description

The core melanoma model aims to be a representation of melanoma cells that accounts for the main drivers of cutaneous melanoma and can use these drivers to predict their response (in terms of proliferation and apoptosis) to a variety of external stimuli and targeted therapies. It focuses on BRAF- and NRAS-driven tumours as these make up a significant fraction of cutaneous melanomas (*43*) and are the focus of nearly all experimental investigations. While the model has not been tested on NF1-driven tumours, due to a lack of representation in literature, it is likely it would behave well as NF1 is an inhibitor of RAS (*110*), and NRAS-driven tumours have been tested. "Triple-wild-type" tumours without

mutations in any of these genes make up a small but significant fraction of melanomas but are thought to be a heterogeneous group (*43*), and it is unclear how well the model would be able to predict the behaviour of any of these melanomas.

Melanoma cell lines or pre-clinical models are simulated using the known driver mutations present in these cells (Supplementary Table 13). Note that while melanomas do not always show CDKN2A/B mutations, they nearly always show either mutations or loss of expression of these genes (*77*). Therefore, we set the CDKN2AB node to 0 for all melanoma cells. The model attempts to simulate the impact of MITF (varying between 0 and 3) on the transcriptional state (*44*) but regulation of the MITF state itself is considered outside of the scope of the model. Information on the *MITF*-status of cell lines or tumours is not always provided so it is assumed that melanoma cells are in the *MITF*^{high} (MITF = 2) state unless there is information to the contrary.

Each of the nodes in the network is assigned to one of four “pathways” generally describing their behaviour, we will briefly outline each of these below.

Growth Signalling pathway

The growth pathway describes general growth signalling, usually mediated by growth factors and receptor tyrosine kinases, but commonly aberrantly activated in melanoma. This consists of the MAPK and PI3K pathways and their downstream targets. The model includes two inputs which are necessary for growth signalling – a generic growth factor and cell-cell contact, which is required for the maintenance of normal melanocytes. This growth factor is kept generic as many factors have been implicated either in the normal growth of melanocytes (for example ET-1 and FGF (*111*)) or in resistance to targeted therapies (for example by upregulation of RTKs like EGFR or PDGFR (*112*)), and they all converge on the same pathways (*113*). The level of growth factors ranges from 0-4, accounting for starvation (0), levels in standard media (1), levels used to stimulate melanocyte growth (2) and high levels that can drive melanoma growth (4). Activation of RTKs and FAK (via cell-cell contact signalling) leads to activation of PI3K and RAS, which activates both PI3K and RAF.

The MAPK pathway progresses by activation of RAF, MEK and ERK. While feedback from the MAPK pathway has been described (*114*), we have been unable to link these effects to the changes in steady state behaviour in terms of apoptosis and proliferation that are functionally important for this kind of modelling and so this has been excluded. In order to represent the complex interaction between different mutations observed in melanomas with acquired resistance to targeted therapies, MAPK pathway mutations are handled differently to other mutations in the model. Generally, a gain-of-function mutation will mean we set the value of a specific node to its highest value and to model drugs we set the value to 0. For MAPK node we include additional nodes to represent mutations and drugs, which then influence the node itself. For example, in addition to a “BRAF” node, we have a “BRAFV600E” node representing the gain-of-function mutation, a “BRAFamplification” node representing copy number gain of the mutant BRAF and a “BRAFi” node representing a mutant BRAF inhibitor like dabrafenib. The “BRAFV600E” node induces RAS-independent BRAF activity, while the “BRAFamplification” node enhances the degree of RAS-independent BRAF activity. The “BRAFi” node specifically inhibits RAS-independent BRAF activation. This applies to MEK and ERK but not NRAS.

The PI3K pathway includes PTEN, a tumour suppressor gene frequently mutated in melanoma, and an “Akt” node which represent any of the Akt isoforms. Downstream of the

PI3K pathway lies mTORC1 and 2, S6K and GSK3b. The Growth Signalling pathway also includes Myc which is downstream of many pathways including ERK, β-catenin, GSK3b, TGF-β and p53.

The Growth Signalling pathway is largely stand-alone, but it does have some feedback from the rest of the model. The RTK node can be upregulated by cJUN, to model the known upregulation of RTKs (*42*, *112*, *115*) in the MITF-low transcriptional state, which is characterised by high cJUN activity (*48*). PTEN is regulated by p53 and cJUN (*116*) and RAF is activated by PKC to model the known action of the tumour promoter TPA (12-O-Tetradecanoylphorbol-13-acetate (*117*)).

Intercellular Signalling pathway

The secreted factors pathway describes how signals from TGF-β, TNF-α, α-MSH and WNT ligands are recognised by melanoma cells and how these signals are propagated. Some of these factors, such as WNT ligands and TGF-β are produced by melanoma cells. TGF-β expression is downstream of the MAPK pathway (*118*) and so is controlled by ERK in the model. The control of expression of WNT ligands is less well understood and so, except for WNT5a which is inhibited by MITF to mirror its association with the low MITF state (*119*), the WNT ligands are inputs to the model. TNF-α can be produced in small amounts by melanoma cells in culture (*36*), but has been thought to be primarily derived from other elements of the tumour environment. However, in the context of the epidermis, we found that melanoma cells had the highest level of *TNF* expression (Figure 3D). The original melanoma model allowed for either possibility by treating the level of TNF-α as an input to the model, while the melanoma-LC includes an in-depth treatment of *TNF* expression (detail provided in main text). α-MSH is produced by keratinocytes (*120*) and is sometimes included in media to culture melanocytes and so is considered an input to the model.

In the base melanoma model, TNF-α signals via the TNFR node and stimulates IKK, leading to activation of NF-κB, although activation of IKK through Akt and ERK and synergistic activation of NF-κB targets by cJUN are also included in the model (*48*). Although TNF-α is known to induce the MITF-low state (*48*, *121*), the exact mechanism behind this switch is poorly understood and in any case, mechanisms of phenotype switching are considered beyond the scope of the model, and so this effect must be taken into account at the point of specifying inputs. TNF-α is known to induce apoptosis in some cell types however it appears to have the opposite effect in melanoma, and in some contexts it even protects from apoptosis (*122*). TNF signalling also activates JNK and p38 (*123*).

The TGF-β pathway is modelled by its receptor, TBR (TGF-βR), and Smad2/3, whose effects are broadly anti-proliferative in melanoma. The receptor TBR is inhibited by MITF to account for the fact that MITF low cells have enhanced TGF-β signalling (124) and induction of low MITF state causes induction of TGFBR2 gene (*112*), while conversely MITF overexpression inhibits TGF-β signal propagation (*125*).

The α-MSH pathway signals through the MC1R receptor leading to production of cAMP, and activation of PKA and CREB (*126*). CREB can also be activated by high levels of ERK activity (*127*) and its downstream target in the model is cJUN (*128*). PKA is also negatively regulated by Smad2/3 (*129*) and can activate β-catenin (*130*).

In melanoma, both canonical and non-canonical WNT signalling have been reported (*119*). Canonical WNT signalling through the Frizzled receptor leads to β-catenin activation through

inhibition of Axin, while multiple canonical WNT ligands (WNT3a, WNT10a, etc) exist they are not generally distinguished in the literature and so a single WNT node is used for canonical WNT ligands. The non-canonical WNT ligand Wnt5a signals through the ROR2 receptor, stimulating PKC and inhibiting the canonical WNT pathway through activation of the Siah2 E3 ligase which targets β-catenin for destruction (*131*). Generally, in melanoma, the two forms of WNT signalling are found to be mutually exclusive and in particular associated with transcriptional state (*124*). Wnt5a in particular is associated with and can induce the MITF low state (*132*), and therefore in the model is inhibited by MITF. Little appears to be known about regulation of WNT ligand expression in melanoma, but we have included the DKK proteins which are intercellular inhibitors of WNT receptor binding that are known to be differentially expressed in melanomas (*39*). The role of β-catenin is not totally clear in melanoma, with some studies arguing for a pro-melanoma role (*133*, *134*) while others argue that loss of β-catenin can enhance melanoma growth (*135*). These contradictions are likely due to the interplay between β-catenin activity and transcriptional state (*136*), where MITF itself has also been seen to have both pro- and anti-proliferative effects in melanoma (*137*, *138*). However, as regulation of MITF is considered beyond the scope of the model, we have not attempted to model the effects of β-catenin in melanoma progression and the only downstream targets of β-catenin, through TCF/LEF1 are p16 and Myc.

Cell Cycle pathway

The cell cycle pathway controls proliferation by modelling the G1/S checkpoint as a function of Cyclin D and CDK4 activity. While other cyclins, CDKs and checkpoints exist, the evidence suggests that this checkpoint is usually the limiting factor in proliferation of melanoma cells. Proliferation is downstream of E2F, which is controlled primarily by pRB which in turn is regulated by the CycD-CDK4 complex. Note that both the proliferation and apoptosis nodes very between 0 and 4 should be interpreted at the population level, so a higher level of proliferation may suggest either a higher fraction of proliferating cells or a shorter time between cell division and similarly for apoptosis.

The tumour suppressor genes p14/ARF (p19ARF in mice), p15 and p16/INK4A are included in the model with p15 and p16 inhibiting CDK4 and ARF targeting both Mdm, which regulates p53, and E2F (*139*). These proteins are expressed from the CDKN2A and CDKN2B genes which are close together in the genome and are frequently mutated in melanoma (*43*, *118)*. Many early papers debated whether genetic loss of factor such as p16 was critical for melanoma development (*140*, *141*). However, it has become increasingly clear that culturing of melanocytes can select for loss of p16 expression at the transcriptional level without underlying mutations (*142*). Furthermore, when a panel of uncultured melanoma cells were examined using DNA and RNA profiling, nearly all had either mutated CDKN2A, lost expression of p16 or had an additional known compensatory mutation in the CDK4 pathway (e.g. CDK4R24C, (*77*)). For these reasons, we assume that loss of CDKN2AB at either the genetic or transcriptional level is a pre-requisite for melanoma formation, and when simulating cell lines, we assume a loss of CDKN2AB even when none is recorded in the literature (as this may be at the transcriptional, not genetic level).

The tumour suppressors p21 and p27 are also included and their activity converges on the CycD-CDK4 complex. p21 lies downstream of p53, MITF, FRA1 and JUNB and is inhibited by Akt and TBX2. p53 itself is regulated by its inhibitor Mdm and a node called

"ReplicationStress", which is used to distinguish normal from transformed melanocytes. Although the exact details of this process are not well understood, this distinction is necessary to understand for example the differential sensitivity of melanocytes and melanoma cells to BH3 mimetic drugs (*143*). Apart from p21, p53 also regulates PUMA and BaxBak, in the survival pathway. p27 is assumed to have a level of constitutive activity in the model but is also activated by Smad2/3 and is inhibited by MITF, Akt, Myc and p90RSK.

MITF is also included in the cell cycle pathway and is an input to the model, as its regulation is highly complex (*144*) and for now we consider it outside the scope of the model. MITF is considered to act as a rheostat in melanoma, having quite different effects at its different levels of activity. At a very high level of MITF expression, cells exhibit a highly differentiated phenotype that is resistant to cell death but is also slowly proliferative. At a high level of MITF expression (which can be considered the "normal" level for melanoma cells), cells are proliferative. At a low level of MITF expression, cells become de-differentiated and are more mobile and less proliferative but more resistant to therapy due to upregulation of RTKs. Complete loss of MITF expression is highly detrimental to cells and is not seen in melanoma cells without experimental perturbation. It is worth noting that cells can switch between the MITF-high and MITF-low without mutation and that any population of cells will likely include some of each type (*145*). Furthermore, recent single-cell RNAseq experiments have dissected these states in even greater detail, and in fact it is possible that further subtypes dependent on other factors such as SOX10 may define the phenotype in greater detail (*55*, *146*–*149*). However, in the interests of keeping the model focused, we decided to limit the model's treatment of MITF to a simple rheostat. MITF regulates many other proteins in the model, which will be described as the arise in the text. One key downstream target of MITF is cJUN, which is thought to positively define the transcriptional program underlying the MITF low state (*48*, *150*). cJUN has various pro-melanoma roles, including upregulation of RTKs, inhibition of PTEN and co-activation of NF-κB targets.

Note that the model does not deal with senescence in melanoma. Short-term growth followed by stable growth arrest in naevi, which usually remain benign for decades, is a characteristic feature of melanocytes with oncogenic mutations (*151*). However, studies into senescence are plagued by irreproducibility and inconsistent definitions, at least in part due to significant differences between *in vivo* and *in vitro* conditions. As tools have developed to a point where naevi can be studied *in vivo*, a picture has emerged suggesting naevi formation is mediated by secreted factors rather than cell intrinsic factors (*24*). While p14, p15 and p16 are upregulated in naevi, there is significant evidence that genetic or transcriptional changes beyond loss of these factors are required for transition from naevus to melanoma (*141*, *152*, *153*). Longitudinal profiling has helped to identify CDKN2A and TERT promoter mutations as unlikely to occur in naevi but common in melanoma (*77*). For now, we largely follow McNeal *et al.* (2015) (*118*) for our treatment of naevi, but this is not a focus for the model and so they are largely avoided in the specification. Note that we also avoid discussion of TERT promoter mutations as these are complex to model, they are only important after tens of rounds of divisions, and they simply allow for long term growth without interacting with other parts of the melanoma network.

Survival pathway

The survival pathway describes how signals from the rest of the model are translated into apoptotic signals in the cell. As described above, the primary pro-apoptotic signal in the cell

is activation of BaxBak by p53, which leads to mitochondrial outer membrane permeabilization (MOMP). This process is opposed by Bcl-2, Mcl-1 and BCL2A1, which are inhibited by PUMA, BIM and BAD, which are, in turn inhibited by growth signalling from the MAPK and PI3K pathways.

MOMP leads to cell death by both caspase-dependent and caspase-independent pathways. The caspase-dependent pathway is initiated by caspase 3 activation but in melanoma is often not significant (*154*) due to expression of the caspase inhibitor ML-IAP (*155*). In order to balance loss of ML-IAP expression in the MITF low state, we have included an edge linking NF-κB to expression of IAP (a node comprising cIAP1/2), a known NF-κB target in fibroblasts (*156*). Caspase-independent death is mediated by AIF, which can also be activated by extremely high levels of MAPK signalling via PARP and PAR (*157*).

Melanoma model specification deviations

Line numbers correspond to Supplementary Table S6. Note that “numerical deviations”, where the overall behaviour of the system is maintained but there are differences in the exact level of the node, are not described in detail here.

- Line 21: McNeal *et al*, (2015) (*118*) state that p16 overexpression has no effect on growth of melanoma cells, in the model this has an effect. This is because the role of p16 in melanoma is unclear, but expression is frequently lost, suggesting a functional role.
- Line 23: McNeal *et al*, (2015) (*118*) state that loss of p16 does not allow melanoma cells to grow, while loss of p15 does. As above, the assumption that p16 has a functional role necessitates that this experiment is not matched.
- Line 47: Zhuang *et al*, (2008) (*158*) state that cMyc is essential for growth of NRAS-driven melanoma cells. This is not true in the model, as here growth arrest caused by loss of cMyc is mediated by loss of CycD expression of p27 activity, both of which are also downstream of PI3K/Akt activity, which is known to lower dependence of melanoma cells on MAPK pathway.
- Line 51: Solit *et al*, (2006) (*159*) show that NRAS-driven tumours are less sensitive to MEKi than BRAF-driven tumours, even growing in their presence. This result is supported to some extent by Kwong *et al*, (2012) (*160*) but is in disagreement with Posch *et al*, (2013) (*161*). The model currently predicts that the degree of apoptosis induced by MEKi will be reduced in NRAS tumours compared to BRAF-driven tumours, but proliferation will be the same.
- Line 66: Verhaegen *et al*, (2006) (*143*) state that knock down of p53 with shRNA reduces but does not eliminate apoptosis, whereas the model predicts it would totally prevent apoptosis. One explanation is that this experiment may not represent a total loss of p53 activity. On a more general note, there is a lack of mechanistic information to describe how melanoma, but not melanocytes, are susceptible to induction of apoptosis but in the model, this is entirely mediated by p53 activation downstream of replication stress.
- Line 85: Konieczkowski *et al*, (2014) (*115*) claim that BRAFi and MEKi but not ERKi can be overcome by AXL overexpression whereas in the model AXL overexpression can overcome BRAFi but not MEKi or ERKi. In the model, ERK is controlled by MEK so there is no way for these to have different effects in this experiment.

- Line 94: Shi *et al*, (2011) (*162*) state that M395 is susceptible to BRAFi vemurafenib however Su *et al*, (2017) (*55*) show that M395 is an MITF-low cell line, which Müller *et al*, (2014) (*163*) show makes melanoma resistant to BRAFi. It is worth noting that transcriptional state can change as a result of culture conditions or between labs. However, without evidence to the contrary we must assume that M395 was MITF low in the hands of Shi. (Note that the model matches data when M395 is labelled as MITF high).
- Line 97: As above.
- Line 104: Wagle *et al*, (2014) (*164*) find that ERKi is as effective against A375 cells even in the presence of the MEK2Q60P mutation. This is an issue as experiments of Moriceau *et al*, (2015) (*54*) show that MEK mutations can overcome BRAFi+MEKi in combination with BRAF amplification, suggesting that they work additively. As ERKi is treated as a dosable variable here, we would not expect low dose ERKi to overcome a combination of BRAF and MEK hyper-activity.
- Line 111: Van Allen *et al*, (2014) (*165*) found that MITF overexpression restored growth of melanoma cells treated with BRAFi. Generally, effects of high levels of MITF expression are anti-proliferative as they enforce a differentiated state, which is reflected in the model.
- Line 118: Moriceau *et al*, (2015) (*54*) show that a MEK Gain of Function (GoF) mutation requires additional BRAF amplification to restore growth to cells treated with BRAFi+MEKi but the model suggests that MEK GoF mutation alone is sufficient. This is because MEK GoF acts independently of MEKi (as shown by Wagle *et al*, (2014) (*164*)) meaning there is no contribution from BRAF required to overcome this.
- Line 123: Moriceau *et al*, (2015) (*54*) show that ERKi can actually enhance growth of cells with BRAF amplification and MEK GoF mutations however low level ERKi takes these cells to ERK=4, which is still sufficient for anti-proliferative activity (although not apoptosis) as per Hong *et al*, (2017) (*157*). As these are colony formation assays, they are only semi-quantitative, and it is hard to disassociate effects of rate of proliferation and apoptosis.
- Line 124: As above for high level BRAF amplification instead of mid level BRAF amplification and MEK GoF.
- Line 127: Moriceau *et al*, (2015) (*54*) found that a M395 strain which acquired resistance to BRAFV600E through BRAF amplification grew normally on drug. Note that if we assume M395 has MITF = 2, this is true but as described above Su *et al.* (2017) (*55*) found that M395 is MITF low.
- Line 128: Combination of above effect of M395 being MITF low and known growth inhibition caused by BRAF amplification (see Das Thakur *et al*, (2013) (*53*)) means no growth predicted by model.

Melanoma-LC model specification deviations

Line numbers correspond to Supplementary Table S9. These issues are in addition to those outlined above, unless otherwise indicated. Unlike those listed above, these issues are "numerical deviations" where the behaviour of the system is not seriously affected, but the exact numbers do not match.

- Lines 43, 45, 47, 49: Zhuang *et al*, (2008) (*158*) found that knockdown of Myc with shRNA leads to reduction of proliferation in both the SK-MEL-2 and SK-MEL-19 backgrounds, with the same effect occurring when combined with p53 shRNA. The

melanoma-LC model finds two steady states when simulating these experiments, in one the correct behaviour is identified and in the other the TNF-α signalling loop keeps JNK activity high, leading to enhanced cJUN activity and reducing the impact of Myc shRNA. As these experiments were performed in cell culture, and we found that YUMM1.7 cells in cell culture do not produce substantial TNF-α, it is possible that their measurements were of cells without engagement of the signalling loop.

- Line 71: Posch *et al*, (2013) (*161*) found that a combined PI3K/mTORC1/2 inhibitor (GSK2126458) reduced proliferation of d04 cells. Similar to above, our simulations of the melanoma-LC model found two steady states divided on engagement of the TNF-α signalling loop. In the state with TNF-α signalling engaged, a low level of proliferation is maintained.

Supplementary Data

SupplementaryData1.xlsx Fixed points of the network under single and double perturbations of the network, as determined by computational screening. Perturbations refer either to alterations to specific node (level specified in data file) or simulation of targeted therapy (levels of affected nodes specified in Supplementary Table S11). Each line refers to the effect of a specific perturbation on a single output node (melanoma cell apoptosis or proliferation or Langerhans cell apoptosis, proliferation or residency).

SupplementaryReferences.xlsx Full references for literature cited in the supplementary tables.

Supplementary Figures

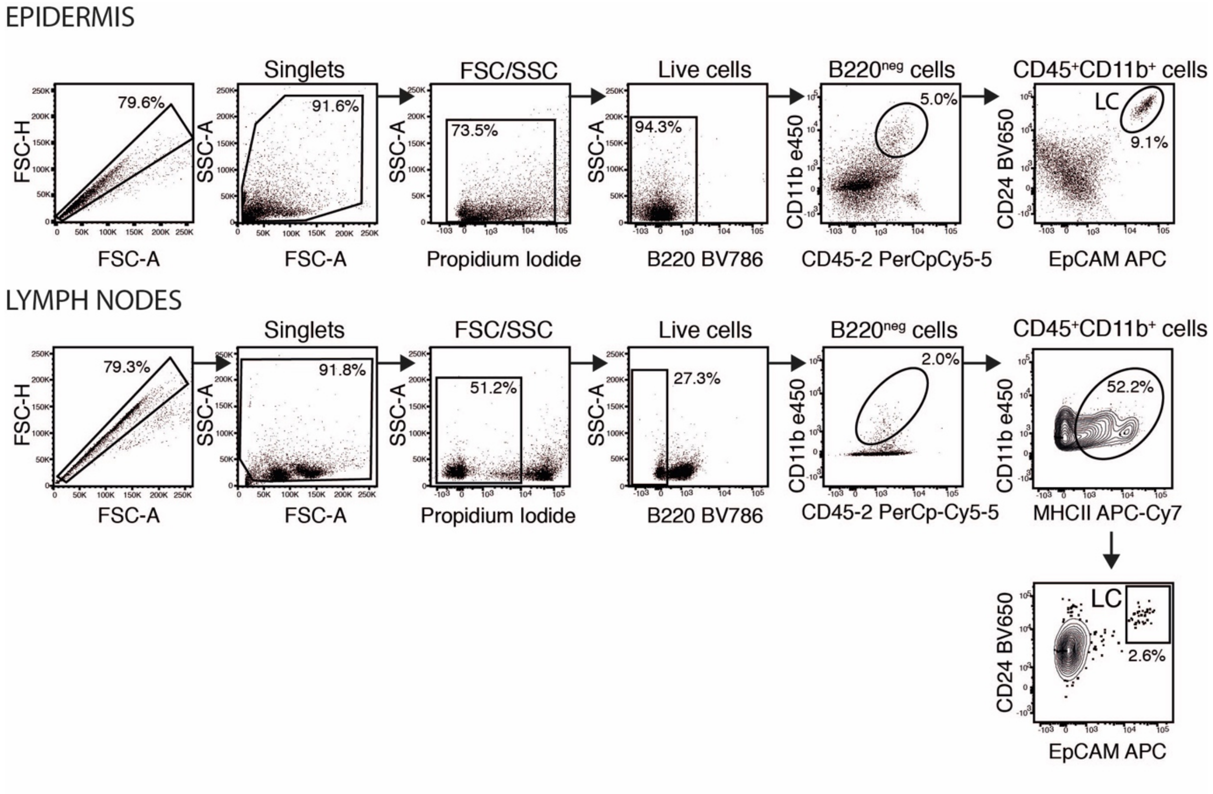

Figure S1. Gating strategy to isolate LCs. Representative flow cytometry plots show the gating strategy used to identify LCs within the epidermis (top) and LNs (bottom) of tumour-bearing mice.

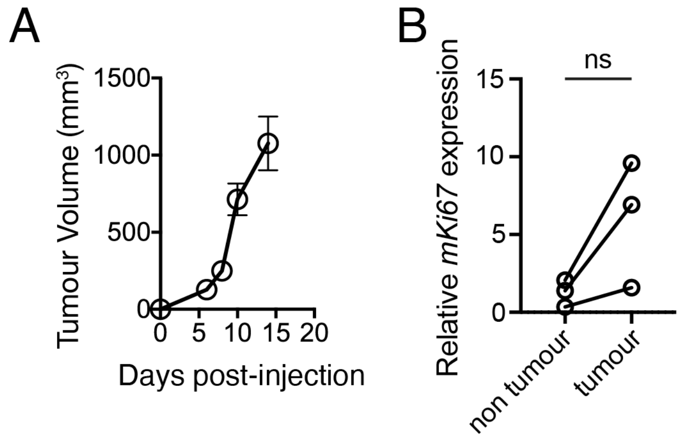

Figure S2. Characterisation of YUMM tumours. A) Line graph showing the increase in volume ± SD of YUMM1.7 cells after intra-dermal injection, showing one representative experiment from more than 3 independent experiments, n = 4 mice. B) Expression of *KI67* in LCs from either melanoma-adjacent or unrelated epidermis. Circles are independent samples pooled from 2 independent experiments. Expression measured by rtPCR, p-values calculated by Wilcoxon matched pair rank test, *ns* indicates p>0.05.

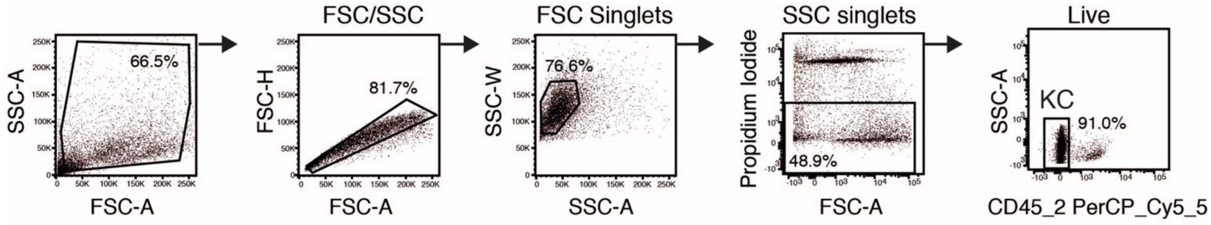

Figure S3. Gating strategy to isolate KCs. Representative flow cytometry plots show the gating strategy used to identity and sort epidermal KCs.

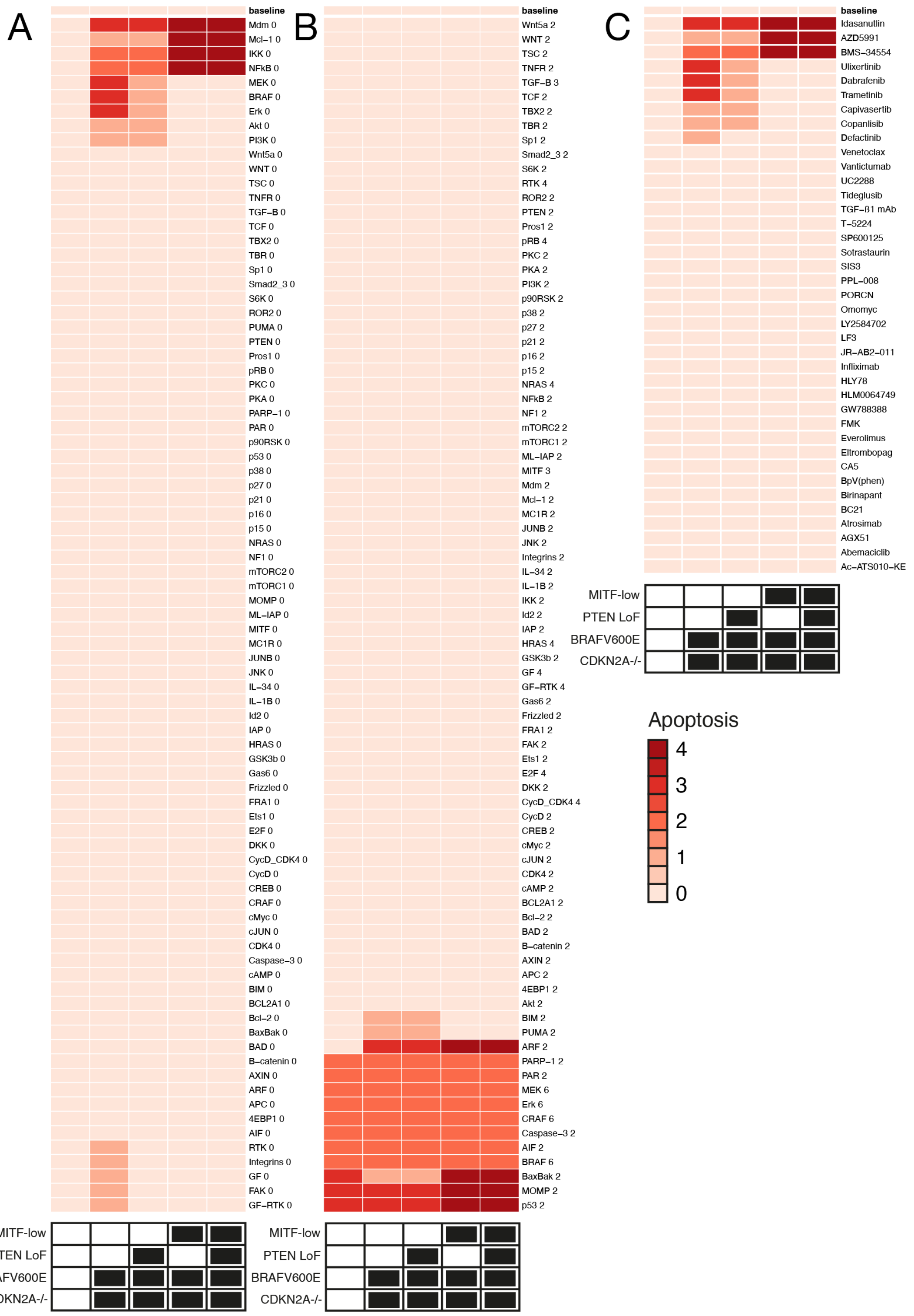

Figure S4: Predicted impact of single mutations and drugs on melanoma cell apoptosis. A-C) Heatmaps showing effect on melanoma cell apoptosis of loss-of-function mutations, gain-of-function mutations and targeted therapies. All data shown in 4 melanoma backgrounds indicated below main plot and a healthy skin control. The colour of each cell corresponds to level of melanoma cell apoptosis in panels A) and B) the number next to a node represents the value to which the node was set for the perturbation.

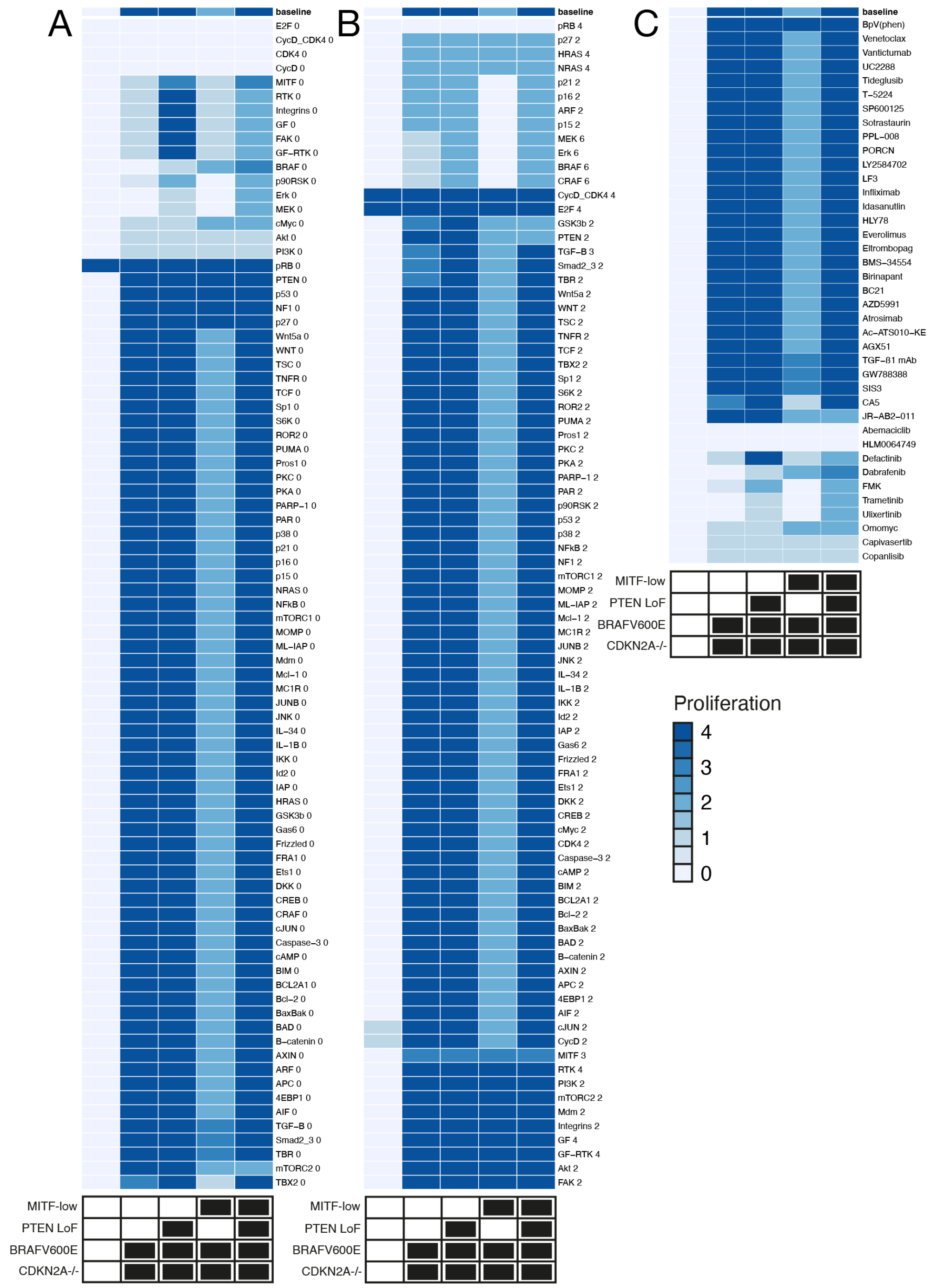

Figure S5: Predicted impact of single mutations and drugs on melanoma cell proliferation. A-C) Heatmaps showing effect on melanoma cell proliferation of loss-of-function mutations, gain-of-function mutations and targeted therapies. All data shown in 4 melanoma backgrounds indicated below main plot and a healthy skin control. The colour of each cell corresponds to level of melanoma cell proliferation in panels A) and B) the number next to a node represents the value to which the node was set for the perturbation.

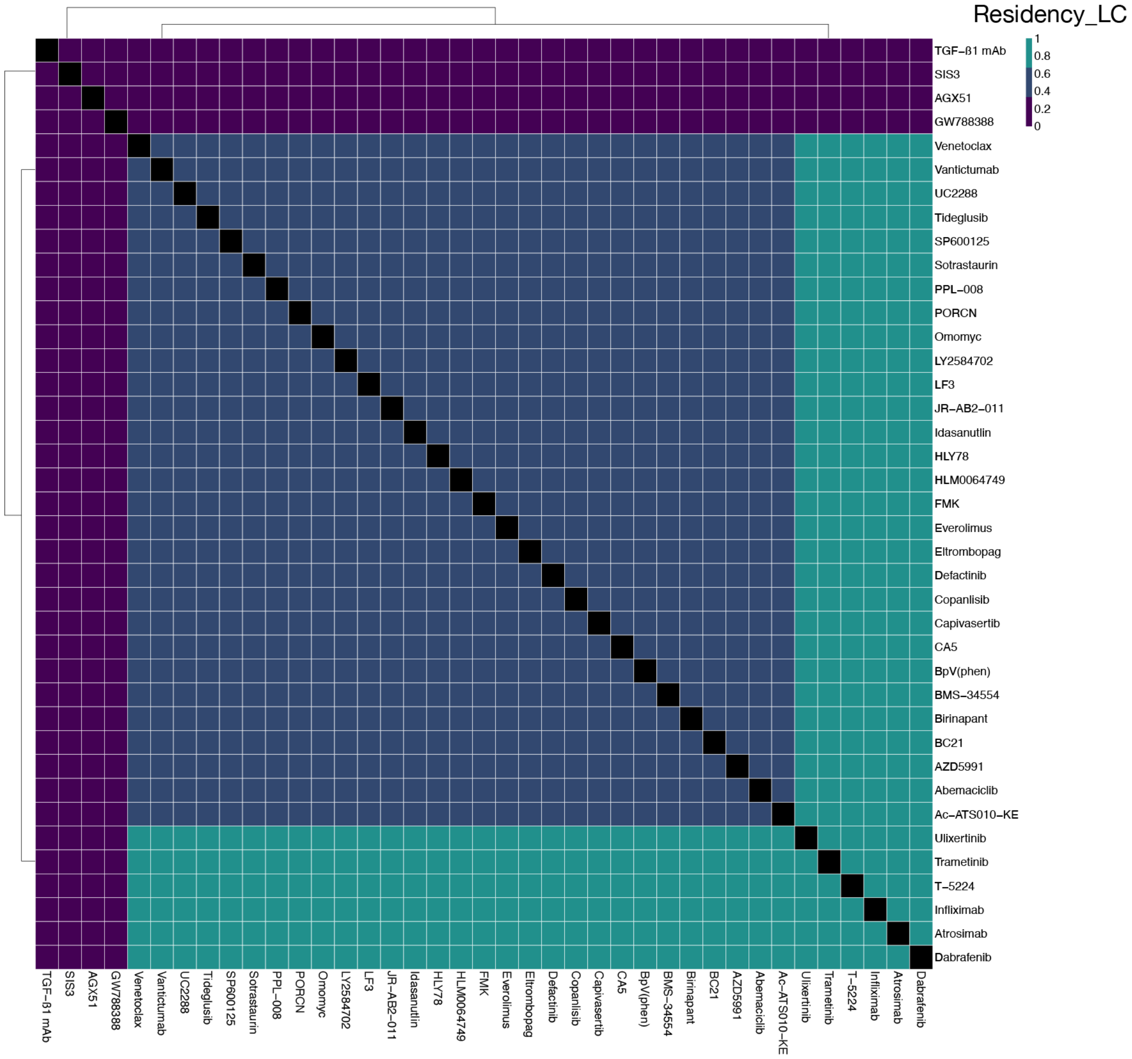

Figure S6: Predicted impact of drug combinations on LC residency. Data shown in BRAFV600E CDKN2A -/- PTEN -/- *MITF*^{high} background. Shade of each cell corresponds to level of LC residency. Drug targets are listed in Supplementary Table S11.

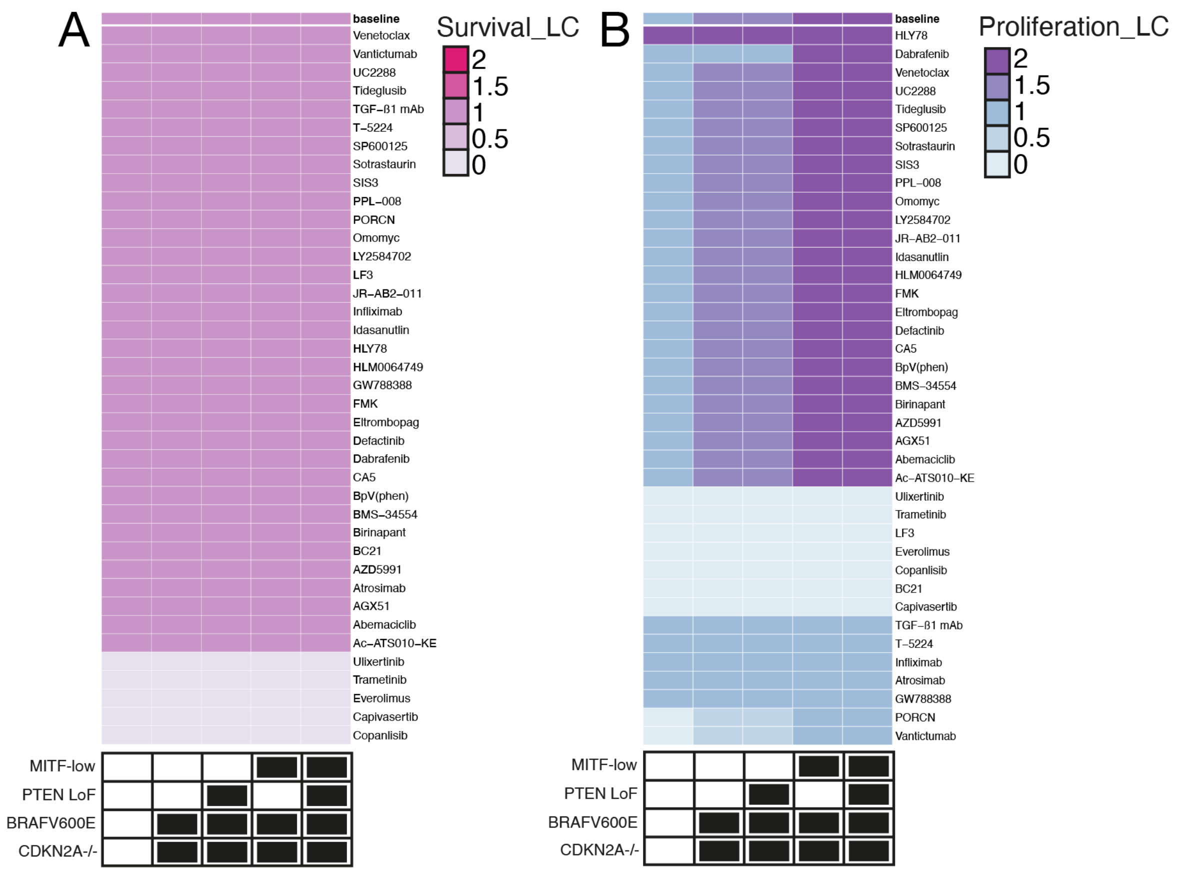

Figure S7: Predicted impact of drugs on LC survival and proliferation. A) Effect of targeted therapies on LC survival. B) Effect of targeted therapies on LC proliferation. All data shown in 4 melanoma backgrounds indicated below main plot and a healthy skin control. The colour of each cell corresponds to level of relevant behaviour node.

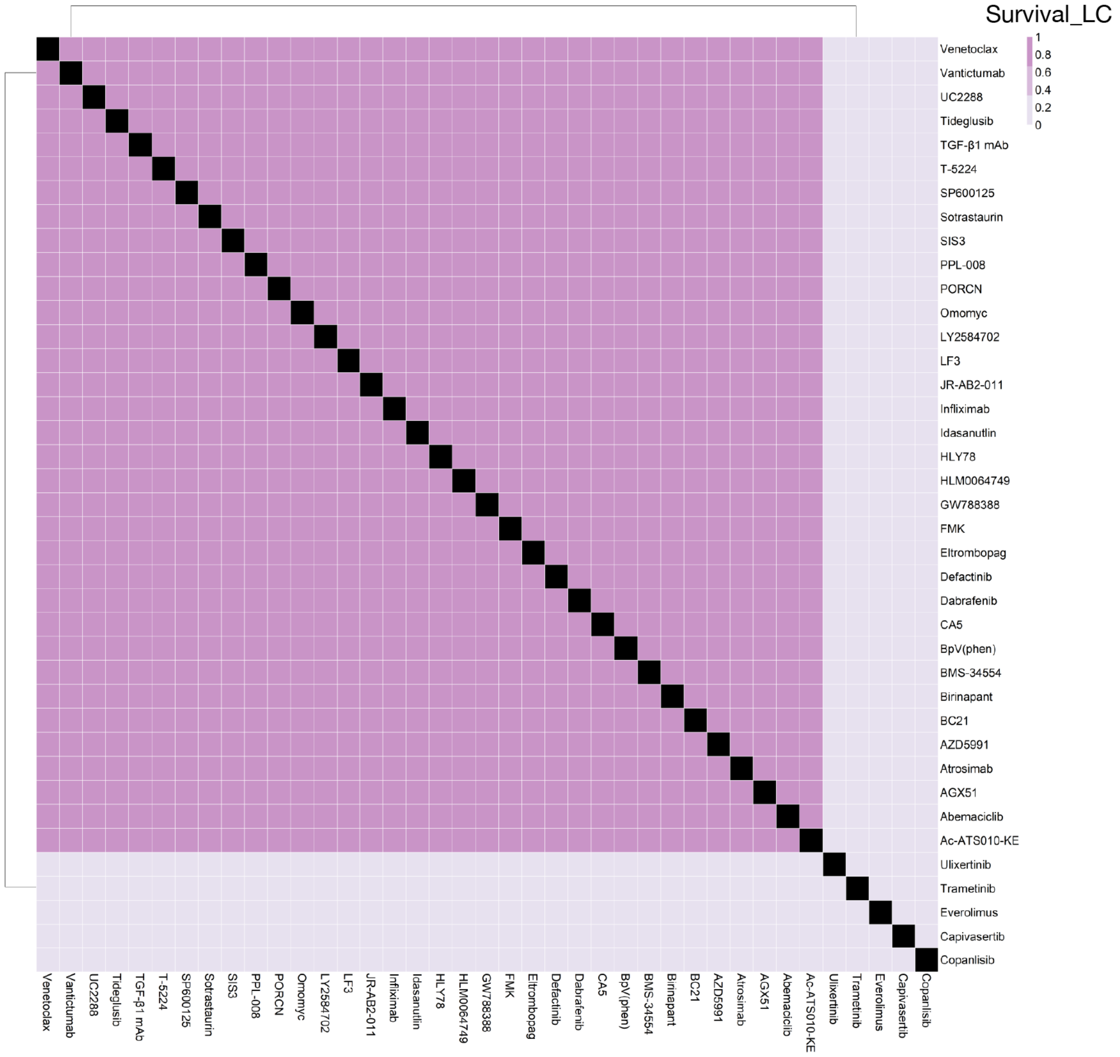

Figure S8: Predicted impact of drug combinations on LC survival. Data shown in BRAFV600E CDKN2A -/- PTEN -/- *MITF*^{high} background. Shade of each cell corresponds to level of LC residency. Drug targets are listed in Supplementary Table S11.

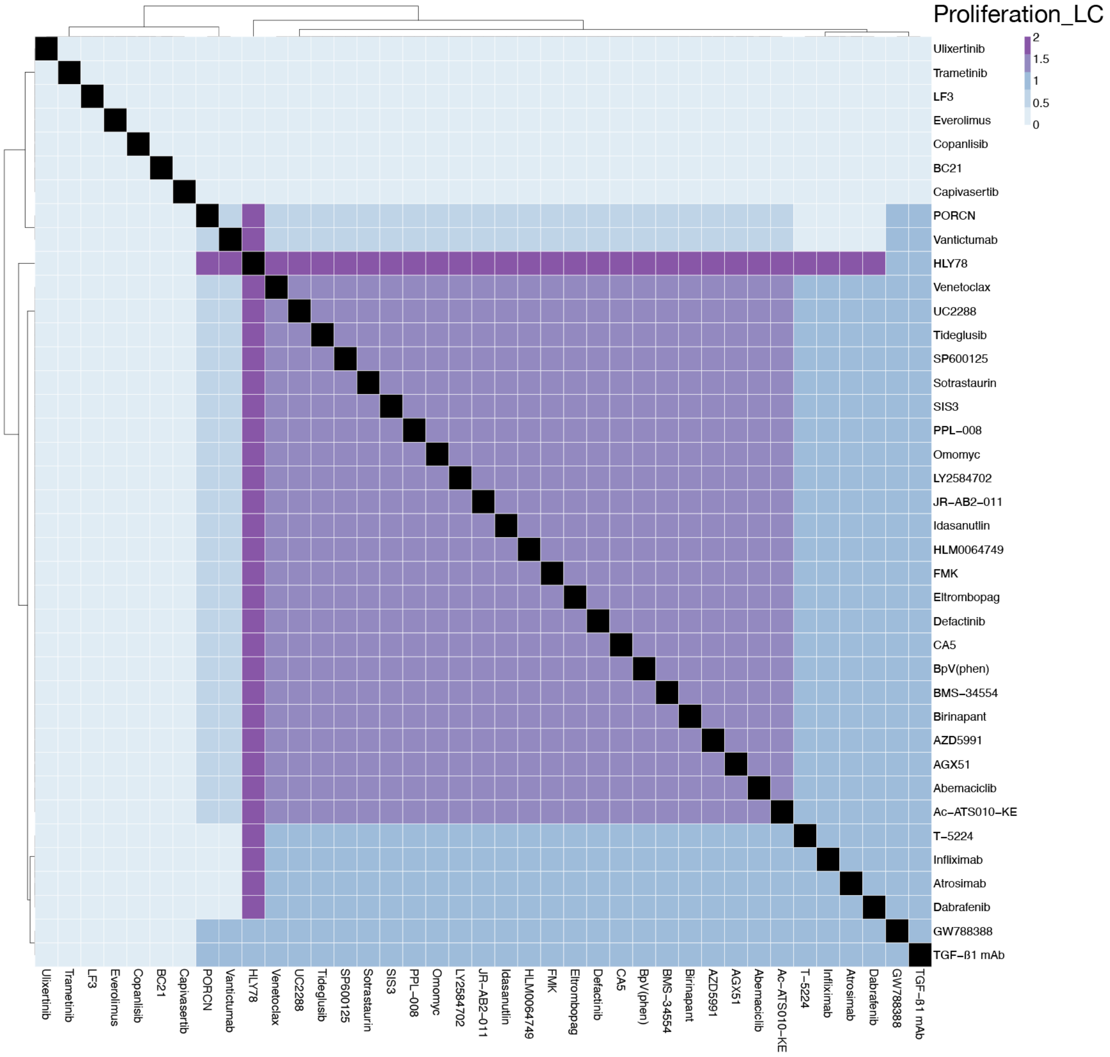

Figure S9: Predicted impact of drug combinations on LC proliferation. Data shown in BRAFV600E CDKN2A -/- PTEN -/- *MITF*^{high} background. Shade of each cell corresponds to level of LC residency. Drug targets are listed in Supplementary Table S11.

Supplementary Table S1. Regulatory edges in the Langerhans cell model

From	To	Type	Reference	Explanation
AdhesionMolecules	Residency_LC	Activator	Deckers et al., Front in Imm (2018)	Adhesion molecules prevent migration
Akt_LC	mTOR_LC	Activator	Liu et al. Nat Rev Mol Cell Bio (2020)	PI3K activates mTORC1 via Akt and Tsc
AXIN_LC	B-catenin_LC	Inhibitor	Zhang, Y. & Wang, X. J Hematol Oncol (2020)	Axin in complex with GSK3b, APC and CK1a inhibits B-catenin activity
Axl	PI3K_LC	Activator	Lemke et al. Nat Rev Immunology (2008)	Pi3K acts downstream of RTKs
Axl	Tyro3	Inhibitor	Bauer, T., et al. J Exp Med (2012)	Loss of Axl leads to uprgulation of Tyro3 and Mer
B-catenin_LC	TCF_LC	Activator	Zhang, Y. & Wang, X. J Hematol Oncol (2020)	B-catenin acts trhough TCF transcription factor
TCF_LC	Proliferation_LC	Activator	Becker, M. R., et al., J Invest Dermatol (2011)	Inhibitor of B-catenin (Dkk1) reduce proliferation
CBF-B	Id2_LC	Activator	Chopin et al., Sem Cell Dev Bio (2015)	Runx3 dimerises with CBF-B proteins
CSF1R	PI3K_LC	Activator	Stanley et al., CSH Pers Bio (2014)	Pi3K acts downstream of RTKs
DKK	Frizzled_LC	Inhibitor	Nakamura, T., et al. J Cell Mol Med (2008)	DKK binds Lrp5/6 preventing formation of Wnt/Frizzled/Lrp5/6 complex
E-cadherin	B-catenin_LC	Inhibitor	Van den Bossche et al., Blood (2012)	E-cadherin binds B-catenin inhibiting it and helping to sequester it at the plasma membrane
E-cadherin	Residency_LC	Activator	Kel et al. J Immunology (2010)	TBR KO leads to E-cadherin downregulation = increased migratory pheno of LCs
ERK_LC	Survival_LC	Activator	Collin et al., Curr Opin Hematology (2016)	Erk activity promotes cell survival and proliferation
ERK_LC	Survival_LC	Activator	Stanley et al., CSH Pers Bio (2014)	Erk activity promotes cell survival and proliferation
ERK_LC	Proliferation_LC	Activator	Collin et al., Curr Opin Hematology (2016)	Erk activity promotes cell survival and proliferation
ERK_LC	Proliferation_LC	Activator	Stanley et al., CSH Pers Bio (2014)	Erk activity promotes cell survival and proliferation
Frizzled_LC	AXIN_LC	Inhibitor	Galluzzi, L., et al., Trends Cell Biol (2018)	Binding of Wnt ligand to Fzd receptor leads to B-catenin activation via AXIN
Gas6	Axl	Activator	Collin et al., Curr Opin Hematology (2016)	Gas6 is a ligand for Axl and Tyro3
Gas6	Axl	Activator	Hieronymous et al., Sem Cell Dev Bio (2015)	Gas6 is a ligand for Axl and Tyro3
Gas6	Tyro3	Activator	Hieronymous et al., Sem Cell Dev Bio (2015)	Gas6 is a ligand for Axl and Tyro3
Id2_LC	Residency_LC	Activator	Sere et al., Immunity (2012)	Long term LCs are Id2 dependent
IL-18	IL-1B	Activator	Griffiths et al. Cytokine (2005)	IL-18 acts upstream of IL-1B
IL-1B	IL-1R	Activator	Griffiths et al. Cytokine (2005)	IL-1B signals mainly through IL-1R1 receptor in LCs
IL-1R	Irf1	Activator	Orzalli et al., Molecular cell (2018)	IL-1B antiviral response if Irf1 dependent
IL-1R	Irf1	Activator	Didovic, S., et al. Eur J Immunol (2016)	IL-1R signalling occurs in KCs not LCs, may be mediated by CCL2 or GM-CSF
IL-1R	TNF-a	Activator	Griffiths et al. Cytokine (2005)	IL-1B stimulates TNF production by keratinocytes
IL-34	CSF1R	Activator	Wang, Y., et al. Nat immunology (2012)	IL-34 is a ligand of CSF1R
Irf1	E-cadherin	Inhibitor	Jakob, T., & Udey, M. C. J immunology (1998)	TNFalpha and IL-1B signalling downregulate E-cadherin at mRNA level
Irf4	Residency_LC	Inhibitor	Sirvent et al. Nat Comms (2020)	Bulk RNA-seq
LAMTOR-p14	mTOR_LC	Activator	Collin et al., Curr Opin Hematology (2016)	LAMTOR-p14 is an adaptor complex that contributes to mTOR and ERK activation
LAMTOR-p14	ERK_LC	Activator	Collin et al., Curr Opin Hematology (2016)	LAMTOR-p14 is an adaptor complex that contributes to mTOR and ERK activation
LAMTOR-p14	ERK_LC	Activator	Nada, S. et al. Embo J (2009)	LAMTOR complex facilitates MEK-ERK interaction
MEK_LC	ERK_LC	Activator	Shaul and Seger, BBA, (2007)	Thr183, Y185 phosphorylation
mTOR_LC	Survival_LC	Activator	Collin et al., Curr Opin Hematology (2016)	kinase (growth/metabolism)
mTOR_LC	Proliferation_LC	Activator	Collin et al., Curr Opin Hematology (2016)	kinase (growth/metabolism)
PI3K_LC	Akt_LC	Activator	Liu et al. Nat Rev Mol Cell Bio (2020)	PI3K activates mTORC1 via Akt and Tsc
Pros1	Tyro3	Activator	Lemke et al. Nat Rev Immunology (2008)	Pros1 is a ligand for Tyro3
Pu.1	Runx3	Activator	Chopin, M., et al. J Exp Med (2013)	myeloid TF
Pu.1	Runx3	Activator	Chopin et al., Sem Cell Dev Bio (2015)	myeloid TF
Pu.1	Runx3	Activator	Deckers et al., Front in Imm (2018)	myeloid TF
Runx3	Id2_LC	Activator	Collin et al., Curr Opin Hematology (2016)	TF, dimerises with CBF-B
Smad2_3_LC	Pu.1	Activator	Collin et al., Curr Opin Hematology (2016)	Downstream signal pathway of TGFb
TBR_LC	E-cadherin	Activator	Hieronymous et al., Sem Cell Dev Bio (2015)	cytokine receptor
TBR_LC	AdhesionMolecu	Activator	Yasmin et al., JID (2013)	TGFb enhances the expression of epithelial adhesion molecules
TBR_LC	Smad2_3_LC	Activator	Collin et al., Curr Opin Hematology (2016)	TGFB1 receptor complex
TBR_LC	Axl	Activator	Bauer, T., et al. J Exp Med (2012)	Western blot after using TGFbR1 receptor blockers
TBR_LC	LAMTOR-p14	Activator	Collin et al., Curr Opin Hematology (2016)	TGFB1 receptor complex
TGF-B	TBR_LC	Activator	Hieronymous et al., Sem Cell Dev Bio (2015)	cytokine
TNF-a	TNFR_LC	Activator	Griffiths et al. Cytokine (2005)	TNF-a signals mainly through TNF-R2 receptor in LCs
TNFR_LC	Irf1	Activator	Davies et al. Front Imm (2021)	Single cell RNA-seq
Tyro3	PI3K_LC	Activator	Lemke et al. Nat Rev Immunology (2008)	Pi3K acts downstream of RTKs
WNT	Frizzled_LC	Activator	Galluzzi, L., et al., Trends Cell Biol (2018)	Binding of Wnt ligand to Fzd receptor leads to B-catenin activation
CSF1	CSF1R	Activator	Wang, T. et al. Pigm Cell Melanoma R (2012)	Melanoma cells produce CSF1 (M-CSF)
CSF1	CSF1R	Activator	Hamilton, J. A. Nat Rev Immunol (2008)	CSF1 is ligand for CSF1R

Supplementary Table S1. Regulatory edges in the Langerhans cell model. Each row shows a single edge, originating from the node in the "from" column and directed to the node in the "to" column. The table also contains details of the type of edge, a brief explanation of the mechanism of this regulation and a reference to the literature where required.

Supplementary Table S2. Target functions used in the Langerhans cell model

Node	Target Function	Reference	Explanation
AdhesionMolecules	generic		
Akt_LC	generic		
AXIN_LC	2-var(Frizzled_LC)	Galluzzi, L., et al., Trends Cell Biol (2018)	Binding of Wnt ligand to Fzd receptor leads to B-catenin activation via AXIN
Axl	var(Gas6)*var(TBR_LC)	Collin et al., Curr Opin Hematology (2016)	Axl is induced by TGF signalling, Gas6 is ligand for Axl
Axl	var(Gas6)*var(TBR_LC)	Bauer, T., et al. J Exp Med (2012)	Axl is induced by TGF signalling, Gas6 is ligand for Axl
B-catenin_LC	3-var(AXIN_LC)-var(E-cadherin)	Zhang, Y. & Wang, X. J Hematol Oncol (2020)	Axin in complex with GSK3b, APC and CK1a inhibits B-catenin activity
B-catenin_LC	3-var(AXIN_LC)-var(E-cadherin)	Van den Bossche et al., Blood (2012)	E-cadherin binds B-catenin inhibiting it and helping to sequester it at the plasma membrane
CBF-B	1		
CSF1	0		
CSF1R	max(var(IL-34), var(CSF1))	Hamilton, J. A. Nat Rev Immunol (2008)	CSF1R is receptor for both IL-34 and CSF1
DKK	1		
E-cadherin	generic		
ERK_LC	var(LAMTOR-p14)*var(MEK_LC)	Nada, S. et al. Embo J (2009)	LAMTOR complex facilitates MEK-ERK interaction
Frizzled_LC	2*var(WNT)-var(DKK)	Nakamura, T., et al. J Cell Mol Med (2008)	Frizzled is receptor for Wnt, can be inhibited by DKK, but at endogenous levels Wnt signalling still occurs.
Frizzled_LC	2*var(WNT)-var(DKK)	Becker, M. R., et al., J Invest Dermatol (2011)	Frizzled is receptor for Wnt, can be inhibited by DKK, but at endogenous levels Wnt signalling still occurs.
Gas6	1		
Id2_LC	var(CBF-B)*var(Runx3)	Collin et al., Curr Opin Hematology (2016)	Runx3 dimerise with CBF-B
IL-18	0		
IL-1B	1+var(IL-18)	Griffiths et al. Cytokine (2005)	IL-1B consitutively present in epidermis, increased levels trigger LC migration
IL-1R	generic		
IL-34	1		
Irf1	var(TNFR_LC)*var(IL-1R)	Griffiths et al. Cytokine (2005)	Combination of 2 cytokine signals are needed to induce LC migration
Irf4	1		
LAMTOR-p14	1+var(TBR_LC)	Collin et al., Curr Opin Hematology (2016)	LAMTOR complex responds to TGF signalling
MEK_LC	1		
mTOR_LC	min(var(LAMTOR-p14),var(Akt_LC))	Collin et al., Curr Opin Hematology (2016)	LAMTOR complex acts as adaptor to aid in mTOR activation, which is also mediated by PI3K phosphorylation
mTOR_LC	min(var(LAMTOR-p14),var(Akt_LC))	Liu et al. Nat Rev Mol Cell Bio (2020)	LAMTOR complex acts as adaptor to aid in mTOR activation, which is also mediated by PI3K phosphorylation
mTOR_LC	min(var(LAMTOR-p14),var(Akt_LC))	Laplante, M. & Sabatini, D. Cell (2012)	Functional AND gate between TSC and LAMTOR dependent mechanisms of mTOR activation
PI3K_LC	var(Axl)+var(CSF1R)+var(Tyro3)-1	Stanley et al., CSH Pers Bio (2014)	PI3K acts downstream of RTKs
PI3K_LC	var(Axl)+var(CSF1R)+var(Tyro3)-1	Lemke et al. Nat Rev Immunology (2008)	PI3K acts downstream of RTKs
Proliferation_LC	var(TCF_LC)*var(ERK_LC)*var(mTOR_LC)/2		
Pros1	1		
Pu.1	generic		
Residency_LC	floor(avg(var(AdhesionMolecules),var(E-cadherin),var(Id2_LC))) + 1 - ceil(var(Irf4)/2)		
Runx3	generic		
Smad2_3_LC	generic		
Survival_LC	min(var(ERK_LC),var(mTOR_LC))		
TBR_LC	generic		
TCF_LC	generic		
TGF-B	1		
TNF-a	var(IL-1R)-1	Griffiths et al. Cytokine (2005)	Production of TNF-a by KCs is induced by high elvels of IL-1B
TNFR_LC	generic		
Tyro3	max(var(Pros1),var(Gas6))*max(0,1-var(Axl))	Hieronymous et al., Sem Cell Dev Bio (2015)	Tyro3 downregulated by Axl, Pros1 and Gas6 are ligands
Tyro3	max(var(Pros1),var(Gas6))*max(0,1-var(Axl))	Bauer, T., et al. J Exp Med (2012)	Tyro3 downregulated by Axl, Pros1 and Gas6 are ligands
WNT	1		

Supplementary Table S2. Target functions used in the Langerhans cell model. The target function for each node is specified, along with a brief description of the mechanism and a reference where required. A number in the Target Function column represents nodes that are set to a constant value.

Supplementary Table S3. Experiments from the literature used to validate the Langerhans cell model

Paper	Cell Line	Experiment	Constraints	Expected Results	Model Result	Notes
Kel et al. J Immunology (2010)	Normal Mouse Langerhans Cells	TGF-βR1-/-	TBR_LC OFF	Survival_LC Mid, Residency_LC OFF, Proliferation_LC Mid	Survival_LC Mid, Residency_LC Low, Proliferation_LC Mid	
Sparber, F.,et al. Blood (2014)	Normal Mouse Langerhans Cells	p14-/-	LAMTOR-p14 OFF	Survival_LC OFF, Residency_LC Mid, Proliferation_LC OFF	Survival_LC OFF, Residency_LC Mid, Proliferation_LC OFF	
Cumberbatch, M., Fielding, I., & Kimber, I. (1994)	Normal Mouse Langerhans Cells	TNF-α injection	TNF-a High	Residency_LC OFF	Residency_LC OFF	
Kellersch, B., & Brocker, T. Blood (2013)	Normal Mouse Langerhans Cells	Raptor-/- (essential component of mTORC1)	mTOR_LC OFF	Survival_LC OFF	Survival_LC OFF	
Borkowski, T. A., et al. J Exp Med (1996)	Normal Mouse Langerhans Cells	TGF-β1-/-	TGF-B OFF	Residency_LC OFF	Residency_LC OFF	
Chopin, M., et al. J Exp Med (2013)	Normal Mouse Langerhans Cells	Pu.1-/-	Pu.1 OFF	Residency_LC OFF	Residency_LC OFF	
		Id2-/-	Id2_LC OFF	Residency_LC OFF	Residency_LC OFF	
Bauer, T., et al. J Exp Med (2012)	Normal Mouse Langerhans Cells	Tyro3-/-, Axl-/-	Tyro3 OFF, Axl OFF	Survival_LC OFF	Survival_LC OFF	Also Mertk-/-
		Axl-/-	Axl OFF	Survival_LC Mid	Survival_LC Mid	
Wang, Y., et al. Nat immunology (2012)	Normal Mouse Langerhans Cells	IL-34-/-	IL-34 OFF	Survival_LC OFF	Survival_LC OFF	
Becker, M. R., et al. JID (2011)	Normal Mouse Langerhans Cells	DKK overexpression	DKK High	Proliferation_LC OFF	Proliferation_LC OFF	
Cumberbatch, M.,et al. Immunology (2001)	Normal Mouse Langerhans Cells	IL-18 injection	IL-18 High	Residency_LC OFF	Residency_LC OFF	
		IL-18 injection + anti-TNF-α Ab	IL-18 High, TNF-a OFF	Residency_LC Mid	Residency_LC Mid	
		IL-18 injection + anti-IL-1R Ab	Il-18 High, IL-1R OFF	Residency_LC Mid	Residency_LC Mid	
Cumberbatch, M., et al. Immunology (1997)	Normal Mouse Langerhans Cells	IL-1β injection	IL-1B High	Residency_LC OFF	Residency_LC OFF	
		IL-1β injection +anti-TNF-α Ab	IL-1B High, TNF-a OFF	Residency_LC Mid	Residency_LC Mid	
		TNF-α injection + anti-IL-1β Ab	TNF-a High, IL-1B OFF	Residency_LC Mid	Residency_LC Mid	

Supplementary Table 3. Experiments from the literature used to validate the Langerhans cell model. For each experiment, the table shows the publication, the cell line used, the constraints applied to the model and the expected outcome, based on the experimental result. The sixth column shows the result of the model simulations, which exactly match the expected values.

Supplementary Table S4. Regulatory edges in the melanoma model

From	To	Type	Reference	Explanation
4EBP1	CycD	Inhibitor	Averous et al., Oncogene, (2008)	Translation inhibition
a-MSH	MC1R	Activator	Levy, C. et al. Trends in Mol Med (2006)	a-MSH binds MC1R receptor
AIF	Apoptosis	Activator	Hussein, M. R., et al., J Pathology (2003)	AIF is effector of caspase-independent apoptosis
Akt	p27	Inhibitor	Liang, J.,et al. Nature Medicine (2002)	Thr157-p; cytoplasmic translocation from nucleus
Akt	p27	Inhibitor	Manning and Cantley, Cell, (2007)	
Akt	p21	Inhibitor	Zhou BP et al., Nat Cell Biol, (2001)	Thr 145 phosphorylation
Akt	p21	Inhibitor	Manning and Cantley, Cell, (2007)	
Akt	TSC	Inhibitor	Memmott, R. M., & Dennis, P. A. Cellular Signalling (2009)	Akt regulates mTOR through phosphorylation of TSC
Akt	GSK3b	Inhibitor	Manning and Toker, Cell, (2017)	Ser9 phosphorylation
Akt	GSK3b	Inhibitor	Cross et al. Nature (1995)	
Akt	IKK	Activator	Bai, D., et al., IJC (2009)	Akt phosphorylates IKKa at T23, required for IKK phosphorylation of p65 at S534
Akt	BAD	Inhibitor	Datta et al., Cell, (1997)	Ser136
Akt	BAD	Inhibitor	She et al., Cancer Cell, (2005)	
Akt	BIM	Inhibitor	Madhunapantula, S. V., et al., Cancer Biol Ther (2011)	Akt phopshorylates BIM
APC	B-catenin	Inhibitor	Galluzzi, L., et al. Trends in Cell Biology (2018)	APC, AXIN and GSK3 (and other) form B-catenin destruction complex
ARF	Mdm	Inhibitor	Zhang et al., Cell, (1998)	Proteolytic degradation
ARF	Mdm	Inhibitor	Pomerantz et al., Cell, (1998)	
ARF	E2F	Inhibitor	Ha, L., et al., PNAS (2007)	ARF causes proteosome-dependent degradation of E2F
AXIN	B-catenin	Inhibitor	Galluzzi, L., et al. Trends in Cell Biology (2018)	APC, AXIN and GSK3 (and other) form B-catenin destruction complex
B-catenin	TCF	Activator	Goding, C. R., & Arnheiter, H. Genes & Development (2019)	TCF transcription factor downstream effector of B-catenin
BAD	Bcl-2	Inhibitor	Chen et al., Molecular Cell, (2005)	Bad binds Bcl-2
BaxBak	MOMP	Activator	Mohana-Kumaran et al. PC&MR (2014)	Bax and Bak cause membrane permealization of mitochondria, opposed by Bcl-2 and Mcl-1
Bcl-2	MOMP	Inhibitor	Mohana-Kumaran et al. PC&MR (2014)	Bax and Bak cause membrane permealization of mitochondria, opposed by Bcl-2 and Mcl-1
BCL2A1	MOMP	Inhibitor	Vogler, M. Cell Death Differ (2012)	BCL2A1 opposes mitochondrial membrane breakdown by BAX and BAK
BIM	Bcl-2	Inhibitor	Chen et al., Molecular Cell, (2005)	Binding BH3 domain
BIM	Mcl-1	Inhibitor	Chen et al., Molecular Cell, (2005)	Binding BH3 domain
BRAF	MEK	Activator	Burotto, M. et al. Cancer (2014)	BRAF is MAPKKK, phosphorylates MEK
BRAFamplification	BRAF	Activator	Luebker, S. A. & Koepsell, S. A. Frontiers Oncol (2019)	Amplification of BRAF gene can occur in response to targeted BRAF inhibition
BRAFi	BRAF	Inhibitor	Luebker, S. A. & Koepsell, S. A. Frontiers Oncol (2019)	Mutant BRAF inhibitors specifically inhibit BRAFV600E eg vemurafenib or dabrafenib
BRAFV600E	BRAF	Activator	Davies, H. et al. Nature (2002)	BRAFV600E mutation greatly increases activity of BRAF
cAMP	PKA	Activator	Kaur, A., et al. BJC (2016)	PKA is effector of MC1R signalling
Caspase-3	Apoptosis	Activator	Hussein, M. R., et al., J Pathology (2003)	Caspase-3 is a key effector of apoptosis
CDK4	CycD_CDK4	Activator	Morgan, Nature, (1995)	Complex formation
CDK4	CycD_CDK4	Activator	Maddika et al., Drug Resistance, (2007)	
CDKN2AB	p16	Activator	Sherr, C. J. Nat Rev Cancer (2006)	CDKN2A encodes p16 and p14/ARF
CDKN2AB	ARF	Activator	Sherr, C. J. Nat Rev Cancer (2006)	CDKN2A encodes p16 and p14/ARF
CDKN2AB	p15	Activator	Bennett, D. C. PC&MR (2016)	the CDKN2B locus is located close to CDKN2A and they are frequently lost together
CellCellContact	Integrins	Activator	Paoli, P., et al. BBA - Mol Cell Res (2013)	Cell-Cell contact is signalled through various integrins leading to FAK and Src activation
cJUN	CycD	Activator	Lopez-Bergami, P., et al. (2007)	AP-1 promotes CycD expression
cJUN	PTEN	Inhibitor	Hettinger et al., Cell death & Differentiation (2007)	cJun transcriptionally inhibit PTEN at 5 upstream region of its promoter
cJUN	RTK	Activator	Sensi, M. et al. JID (2011)	Low MITF melanomas express Axl RTK
cJUN	RTK	Activator	Riesenberg, S., et al. Nat Comms (2015)	Low MITF state associated with high cJUN, PDGFR, EGFR and ERBB3
cJUN	NFkB	Activator	Riesenberg, S., et al. Nat Comms (2015)	NF-kB works synergistically with AP-1.
cJUN	NFkB	Activator	Sabio, G., & Davis, R. J. Sem Imm (2014)	AP-1 and NF-kB act synergistically in reglation of genes like TNFa
cJUN	NFkB	Activator	Riesenberg, S., et al. Nat Comms (2015)	AP-1 and NF-kB expression profiles linked in panel of melanoma cell lines
cMyc	p27	Inhibitor	Maddika et al., Drug Resistance, (2007)	Ubiquitine-mediated degradation
cMyc	p27	Inhibitor	Obaya et al., JBC, (2002)	
cMyc	ARF	Activator	Sherr C, Genes & Dev (1998)	Transcriptional activator
cMyc	ARF	Activator	Zindy et al., Genes & Dev, (1998)	
cMyc	CycD	Activator	Mateyak et al., Mol Cel Bio, (1999)	Myc controls expression of cyclin D
cMyc	CycD	Activator	Gartel, A. L. & Shchors, K. Exp Cell Res (2003)	Myc controls expression of cyclin D
cMyc	p15	Inhibitor	Warner, B.J. et al. Mol and Cell Bio (1999)	Downregulation of Myc by TGF-B necessary for p15 induction
CRAF	MEK	Activator	Kyriakis et al., Nature (1992)	Ser218, Ser222 phosphorylation
CRAF	MEK	Activator	Burotto, M. et al. Cancer (2014)	
CREB	cJUN	Activator	Zhang, H., et al. Exp Hematology Oncol (2020)	CREB regulates expression of cJUN
CycD	CycD_CDK4	Activator	Satyanarayana and Kaldis, Oncogene, (2009)	Complex formation
CycD	CycD_CDK4	Activator	Maddika et al., Drug Resistance, (2007)	
CycD_CDK4	pRB	Inhibitor	Maddika et al., Drug Resistance, (2007)	Phosphorylation of pRb
DKK	Frizzled	Inhibitor	Nakamura, T., et al. J Cell Mol Med (2008)	DKK binds Lrp5/6 preventing formation of Wnt/Frizzled/Lrp5/6 complex
E2F	Proliferation	Activator	Johnson, Nature, (1993)	E2F required for G1/S transition
Erk	p16	Activator	Bennett, D. C. PC&MR (2008)	p16 regulation not well understood but induced by BRAF and NRAS, possibly via p38
Erk	ARF	Activator	Levine, A. J. Nat Revs Cancer (2020)	Speculative edge, based on induction ARF in response to RAS activity.
Erk	FRA1	Activator	Hong, A. et al. Cancer Discov (2017)	High levels of ERK induce p21 arrest via Fra1 and JUNB
Erk	JUNB	Activator	Hong, A. et al. Cancer Discov (2017)	High levels of ERK induce p21 arrest via Fra1 and JUNB
Erk	p90RSK	Activator	Shaul and Seger, BBA, (2007)	Thr359, Ser363 phosphorylation
Erk	p90RSK	Activator	Zhao et al., JBC (1996)	
Erk	p90RSK	Activator	Lara et al., Cancer Research, (2013)	
Erk	cMyc	Activator	Sears et al., Genes Dev, (2000)	Ser 62 phosphorylation by Erk increases half life of Myc
Erk	cMyc	Activator	Shaul and Seger, BBA, (2007)	
Erk	TSC	Inhibitor	Ma et al., Cell, (2005)	Ser664 phosphorylation
Erk	GSK3b	Inhibitor	Ding et al., Mol Cell, (2005)	Thr43 phosphorylation primes for RSK Ser9 phosphorylation
Erk	IKK	Activator	Liu, J., et al. Oncogene (2007)	MAPK pathway promotes IKK activity
Erk	CREB	Activator	Johannessen, M. & Moens, U. Front Biosci (2007)	ERK activates MAPKAPKs such as MSK1,2 and MK2 which phosphorylate CREB S133
Erk	TGF-B	Activator	McNeal, A. S., et al. Cancer Discovery (2015)	MAPK activation leads to TGF-B expression (could be mediated by EGR1)
Erk	PUMA	Inhibitor	Cook et al. FEBS J (2017)	ERK control expression of PUMA via FOXO3
Erk	BIM	Inhibitor	Luciano et al., Oncogene, (2003)	Ser69 phosphorylation
Erk	PARP-1	Activator	Hong, A. et al. Cancer Discov (2017)	High levels of ERK activity induce parthanatos
ERKi	Erk	Inhibitor	Arozarena, I. & Wellbrock, C. Ann Transl Medicine (2017)	ERK inhibitors like VTX11E or SCH772984
FAK	HRAS	Activator	Schaller, M. D. BBA - Mol Cell Res (2001)	FAK contributes to RAS activation via Grb2/SOS proteins

FAK	NRAS	Activator	Schaller, M. D. BBA - Mol Cell Res (2001)	FAK contributes to RAS activation via Grb2/SOS proteins
FAK	PI3K	Activator	Paoli, P., et al. BBA - Mol Cell Res (2013)	FAK activates PI3K
FRA1	p21	Activator	Hong, A. et al. Cancer Discov (2017)	High levels of ERK induce p21 arrest via Fra1 and JUNB
Frizzled	AXIN	Inhibitor	Galluzzi, L., et al., Trends Cell Biol (2018)	Binding of WNT to Frizzled receptor results in recruitment of AXIN to plasma membrane and B-catenin stabilisation
GF	GF-RTK	Activator	Easty, D. J., et al. PC&MR (2011)	Generic Growth Factor/Receptor Tyrosine Kinase interaction
GF-RTK	HRAS	Activator	Gentile, A., et al. Cancer and Metastasis Reviews (2008)	RAS is effector of RTK activation (can also activate PI3K directly).
GF-RTK	NRAS	Activator	Gentile, A., et al. Cancer and Metastasis Reviews (2008)	RAS is effector of RTK activation (can also activate PI3K directly).
GF-RTK	PI3K	Activator	Easty, D. J., et al. PC&MR (2011)	RTKs can activate PI3K directly through p85 subunit
GF-RTK	PKC	Activator	Easty, D. J., et al. PC&MR (2011)	RTKs activate PKC via PLCgamma
GSK3b	CycD	Inhibitor	Romero-Pozuelo et al., Cell Reports (2020)	Thr-286 phosphorylation promotes its nuclear export and cytoplasmic localization
GSK3b	CycD	Inhibitor	Liang and Slingerland, Cell cycle, (2003)	Transcriptional activator
GSK3b	cJUN	Inhibitor	Wei, W., et al. Cancer Cell (2005)	Phosphorylation of cJUN by GSK3b targets it for destruction via E3 ligase Fbw7
GSK3b	cMyc	Inhibitor	Sears et al, Genes Dev, (2000)	Thr58 phosphorylation by GSK3B when Erk mediated Ser 62 phosphorylation exists
GSK3b	B-catenin	Inhibitor	Galluzzi, L., et al., Trends Cell Biol (2018)	APC, AXIN and GSK3 (and other) form B-catenin destruction complex
HRAS	CRAF	Activator	Wee and Wang Cancers, (2017)	Ser338 and Y341 phosphorylation facilitated by RAS
HRAS	BRAF	Activator	Burotto, M. et al. Cancer (2014)	Ras activates Raf proteins
HRAS	PI3K	Activator	Rodriguez-Viciana, P. et al., Nature, (1994)	Direct interaction with catalytic subunit of PI3K
IAP	Caspase-3	Inhibitor	Mohana-Kumaran et al. PC&MR (2014)	c-IAPs inhibit caspases, via SMAC/DIABLO
Id2	p15	Inhibitor	Schlegel, N. C. et al. PC&MR (2009)	Id2 opposes TGF-B induced p15 upregulation.
IKK	NFkB	Activator	Aggarwal, B. B., et al. Blood (2012)	IKK activates NF-kB by inhbiting IkB, the repressor of NF-kB
Integrins	FAK	Activator	Paoli, P., et al. BBA - Mol Cell Res (2013)	Cell-Cell contact is signalled through various integrins leading to FAK and Src activation
JNK	cJUN	Activator	Kappelmann, M., et al EJCB (2014)	JNK phosphorylates c-JUN at S63 and S73, activating it.
JUNB	p21	Activator	Hong, A. et al. Cancer Discov (2017)	High levels of ERK induce p21 arrest via Fra1 and JUNB
MC1R	cAMP	Activator	Goding, C. R., & Arnheiter, H. Genes & Development (2019)	MC1R receptor stimulates CAMP signalling
Mcl-1	MOMP	Inhibitor	Mohana-Kumaran et al. PC&MR (2014)	Bax and Bak cause membrane permealization of mitochondria, opposed by Bcl-2 and Mcl-1
Mdm	p53	Inhibitor	Zhang et al., Cell, (1998)	Ubiquitine-mediated degradation
Mdm	p53	Inhibitor	Moll and Petrenko, Molecular Cancer Research, (2003)	central domain of FOXM1c functions as an RB- recruiting negative-regulatory domain
MEK	Erk	Activator	Shaul and Seger, BBA, (2007)	Thr183, Y185 phosphorylation
MEKGoF	MEK	Activator	Luebker, S. A. & Koepsell, S. A. Frontiers Oncol (2019)	Activating MEK mutations such as MEK1F129L or MEK2Q60P
MEKi	MEK	Inhibitor	Luebker, S. A. & Koepsell, S. A. Frontiers Oncol (2019)	Targeted MEK inhibitor eg trametinib
MITF	TBX2	Activator	Bennett, D. C. PC&MR (2008)	MITF induces expression of TBX2
MITF	cJUN	Inhibitor	Riesenberg, S., et al. Nat Comms (2015)	MITF binds to c-Jun enhancer
MITF	p21	Activator	Levy, C. et al. Trends in Mol Med (2006)	High levels of MITF cause differentitation and cell cycle arrest through p16 and p21
MITF	p27	Inhibitor	Carreira et al. Genes & Development (2006)	MITF destabilises p27 via Dia1
MITF	Id2	Inhibitor	Schlegel, N. C. et al. PC&MR (2009)	Invasive phenotype melanoma cells are resistant to repression of Id2 by TGF-B
MITF	TBR	Inhibitor	Hoek, K. S. et al. Pigm Cell Res (2006)	Speculative edge, low MITF state associated with enhanced TGFB signalling
MITF	TBR	Inhibitor	Javelaud, D., et al. PC&MR (2011)	Overexpression of MITF inhibits TGF-B driven induction of GLI2
MITF	TBR	Inhibitor	Sun et al. Nature (2014)	Sox10 KD (nducing MITF low state) leads to TGFBR2 expression
MITF	Wnt5a	Inhibitor	O'Connell, M. P. & Weeraratna, A. T. PC&MR (2009)	Speculative edge, Wnt5a singalling associated with MITF low state
MITF	Wnt5a	Inhibitor	Hoek, K. S. et al. Pigm Cell Res (2006)	Speculative edge, Wnt5a singalling associated with MITF low state
MITF	Wnt5a	Inhibitor	Shaffer, S. M. et al. Nature (2017)	Speculative edge, Wnt5a singalling associated with MITF low state
MITF	BCL2A1	Activator	Haq, R. et al. PNAS (2013)	MITF binds BCL2A1 promoter
MITF	ML-IAP	Activator	Goding, C. R., & Arnheiter, H. Genes & Development (2019)	MITF induces expression of ML-IAP
MITF	ML-IAP	Activator	Saladi et al. PC&MR (2013)	MITF promotes expression of ML-IAP, may also be other anti-apoptotic factors as lower in melanocytes than transformed melanoma cells
MITF	Bcl-2	Activator	Hartman, M. L., & Czyz, M. J Inv Dermatology (2015)	MITF acts as TF for Bcl-2
ML-IAP	Caspase-3	Inhibitor	Mohana-Kumaran et al. PC&MR (2014)	ML-IAP inhibits caspases, via SMAC/DIABLO
MOMP	Caspase-3	Activator	Mohana-Kumaran et al. PC&MR (2014)	Membrane breakdown causes release of cytochrome-c, formation of apoptosome and caspase activation
MOMP	AIF	Activator	Hussein, M. R., et al., J Pathology (2003)	Membrane breakdown leads to release of AIF, causing caspase-independent apoptosis
MOMP	AIF	Activator	Wang et al. Clin Cancer R (2007)	MEKi-induced apoptosis relies on caspase-independent pathway
mTORC1	S6K	Activator	Ma and Blenis, Nature Rev MCB, (2009)	Thr389 phosphorylation
mTORC1	S6K	Activator	Wee and Wang, Cancers, (2017)	
mTORC1	S6K	Activator	Isotani et al., JBC, (1999)	Thr-412 phosphorylation
mTORC1	4EBP1	Inhibitor	Ma and Blenis, Nature Rev MCB, (2009)	Thr37, Thr46 phosphorylation inhibits the 4EBP1-negative regulator of transaltion factor
mTORC1	4EBP1	Inhibitor	Brunn, Science (1997)	
mTORC1	4EBP1	Inhibitor	Wee and Wang, Cancers, (2017)	
mTORC2	Akt	Activator	Sarbassov et al., Science, (2005)	Ser473 phosphorylation
NF1	HRAS	Inhibitor	Vigil, D., et al Nat Rev Cancer (2010)	NF1 is a GAP for Ras
NF1	HRAS	Inhibitor	Maertens, et al. Cancer Discovery (2013)	effects of NF1 not mediated by NRAS in melanoma
NFkB	IAP	Activator	Wang, C.-Y., et al. Science (1998)	NF-kB activates expresion of c-IAP1 and c-IAP2
NFkB	Mcl-1	Activator	Akgul, C. et al CMLS (2000)	Mcl-1 5' region contain NF-kB binding sequence
NRAS	BRAF	Activator	Burotto, M. et al. Cancer (2014)	Ras activates Raf proteins
NRAS	PI3K	Activator	Rodriguez-Viciana, P. et al., Nature, (1994)	Direct interaction with catalytic subunit of PI3K
NRAS	CRAF	Activator	Wee and Wang Cancers, (2017)	Ser338 and Y341 phosphorylation facilitated by RAS
p15	CDK4	Inhibitor	Sharpless & Sherr Nat Revs Cancer (2015)	p15 inhibits formation of CDK4/6 cycD complex
p16	CDK4	Inhibitor	Kim and Sharpless, Cell, (2006)	Binding
p16	CDK4	Inhibitor	Kotake et al., Anticancer Research, (2015)	
p21	CycD_CDK4	Inhibitor	Harper et al., Cell, (1993)	CDK binding functional evidence
p27	CycD_CDK4	Inhibitor	Wander et al., Clin Cancer Res, (2011)	complex formation
p38	cJUN	Activator	Brinkman, B. M. N., et al. J Biol Chem (1999)	p38 activates ATF-2 in repsonse to TNF stimulation, ATF-2 is a co-activator of cJUN
p53	p21	Activator	El Deiry et al., Cell, (1993)	Transcriptional activator
p53	PTEN	Activator	Stambolic et al., Mol Cell, (2001)	Transcriptional activator
p53	cMyc	Inhibitor	Ho et al., Mol Cell Bio, (2005)	Transcriptional repressor, changes in promoter aceylation status
p53	PUMA	Activator	Villunger et al., Science, (2003)	Transcriptional activator
p53	BaxBak	Activator	Hussein, M. R., et al., J Pathology (2003)	p53 contributes to induction of apoptosis through activation of Bax and indirectly through BH3-domain proteins
p90RSK	CycD	Activator	Eisinger-Mathason et al., Steroids, (2010)	Facilitates Transcriptional activation vi cFos and cJun
p90RSK	p27	Inhibitor	Anjum and Blenis, Nat Rev Mol Cell Biol, (2008)	phosphorylation ,Threonine 198, preventing nuclear localisation
p90RSK	GSK3b	Inhibitor	Ding et al., Mol Cell, (2005)	Ser9 phosphorylation

p90RSK	GSK3b	Inhibitor	Anjum and Blenis, Nat Rev Mol Cell Biol, (2008)	
p90RSK	BAD	Inhibitor	Tan Y et al., JBC, (1999)	Ser112 phosphorylation
p90RSK	BAD	Inhibitor	Eisinger-Mathason et al., Steroids, (2010)	
PAR	AIF	Activator	David, K. K., et al., Front Biosci (2009)	PAR induces AIF release
PARP-1	PAR	Activator	David, K. K., et al., Front Biosci (2009)	PARP-1 synthesises PAR
PI3K	Akt	Activator	Stephens et al., Science, (1998)	Thr308 phosphorylation
PI3K	Akt	Activator	Alessi et al., Curr Biol. (1997)	
PI3K	Akt	Activator	Manning and Toker, Cell, (2017)	
PI3K	mTORC2	Activator	Gan et al., JBC, (2011)	Indirect assessment through AKT activation
PKA	GSK3b	Inhibitor	Fang, X. et al. Proc National Acad Sci (2000)	Serine 9
PKA	B-catenin	Activator	Kaur, A., et al. BJC (2016)	PKA phosphorylates B-catenin on S675 (can also inhibit GSK3b)
PKA	CREB	Activator	Johannessen, M. & Moens, U. Front Biosci (2007)	PKA phosphorylates CREB at S133 in response to cAMP signalling
PKC	JNK	Activator	López-Bergami, P., et al. Molecular Cell (2005)	Rack1 acts as an adaptor for PKC phosphorylation
PKC	CRAF	Activator	Ueda, Y., et al., JBC (1996)	TPA activation of MEK/Erk pathway depends on CRAF but not RAS, (may affect BRAF in other cell types?)
PKC	BRAF	Activator	Ueda, Y., et al., JBC (1996)	TPA activation of MEK/Erk pathway depends on CRAF but not RAS, (may affect BRAF in other cell types?)
pRB	E2F	Inhibitor	Weinberg, et al., Cell, (1995)	Transcriptional repression
PTEN	PI3K	Inhibitor	Worby, C. A. & Dixon, J. E. Annu Rev Biochem (2014)	PIP3 phosphatase, acts in opposition to PI3K activity.
PUMA	Bcl-2	Inhibitor	Chen et al., Molecular Cell, (2005)	Inhibiting BAX
PUMA	Mcl-1	Inhibitor	Chen et al., Molecular Cell, (2005)	Binding BH3 domain
ReplicationStress	p53	Activator	Mohana-Kumaran et al. PC&MR (2014)	DNA damage resulting from tumour growth leads to p53 activation via ATM, ATR etc, may also be a result of ROS
ROR2	B-catenin	Inhibitor	O'Connell, M. P., et al. Cancer Discovery (2013)	Wnt5a signalling leads to expression of Siah2 E3 ligase, which targets CTNNB1
ROR2	B-catenin	Inhibitor	Topol, L.,et al. JCB (2003)	Wnt5a signalling inhibits canonical Wnt signalling via Siah2 (not specifically ROR2 here)
ROR2	PKC	Activator	Dissanayake, S. K., et al. JBC (2007).	Wnt5a activates PKC
RTK	GF-RTK	Activator	Easty, D. J., et al. PC&MR (2011)	Generic Growth Factor/Receptor Tyrosine Kinase interaction
S6K	GSK3b	Inhibitor	Zhang et al., Mol Cell, (2006)	Ser9 phosphorylation
S6K	CREB	Activator	Johannessen, M. & Moens, U. Front Biosci (2007)	p70 phosphorylates CREB in vitro, in vivo evidence less clear, could also act via SGK or Akt
Smad2_3	p27	Activator	Lasfar et al. Carcinogenesis (2010)	p27 downstream target of TGF-B causing inhibition of melanoma cells.
Smad2_3	p15	Activator	Warner, B.J. et al. Mol and Cell Bio (1999)	TGF-B induces cell cycle arrest through p15 induction
Smad2_3	p15	Activator	Lasfar et al. Carcinogenesis (2010)	p15 target of TGF-B signalling
Smad2_3	Id2	Inhibitor	Schlegel, N. C. et al. PC&MR (2009)	Proliferative phenotype melanoma cells are susceptible to repression of Id2 by TGF-B
Smad2_3	cMyc	Inhibitor	Warner, B.J. et al. Mol and Cell Bio (1999)	Downregulation of Myc by TGF-B necessary for p15 induction
Smad2_3	PKA	Inhibitor	Pierrat, M.-J., et al. JBC (2012)	TGF-B leads to repression of PKA in melanoma cells
TBR	Smad2_3	Activator	Javelaud, D., et al. PC&MR (2008)	SMAD2/3 complex is effector of TGF-B signalling
TBX2	p21	Inhibitor	Vance, K. W., et al . Cancer Research (2005)	TBX2 interacts with HDAC1, targeting it to p21 promoter
TBX2	p21	Inhibitor	Bennett, D. C. PC&MR (2008)	TBX2 is anti-senenscence factor that regulates expression of ARF and p21
TBX2	ARF	Inhibitor	Bennett, D. C. PC&MR (2008)	TBX2 is anti-senenscence factor that regulates expression of ARF and p21
TCF	p16	Inhibitor	Delmas, V., et al. Genes & Development (2007)	TCF/LEF binds p16INK4a promoter
TCF	cMyc	Activator	He, T.-C., et al., Science (1998)	TCF promotes expression of MYC gene
TGF-B	Smad2_3	Activator	Javelaud, D., et al. PC&MR (2008)	SMAD2/3 complex is effector of TGF-B signalling
TNF-a	TNFR	Activator	Aggarwal, B. B., et al. Blood (2012)	TNFR1&2 are receptors for TNF-a
TNFR	JNK	Activator	Aggarwal, B. B., et al. Blood (2012)	TNFR leads to activation of JNK (and p38,Erk) via TRAF2 ,MEKK1 and MKK7
TNFR	IKK	Activator	Aggarwal, B. B., et al. Blood (2012)	TNF induces IKK activation through TRAF2
TNFR	p38	Activator	Sabio, G., & Davis, R. J. Sem Imm (2014)	p38 activated by TNF via MKK3 and MKK6
TNFR	p38	Activator	Brinkman, B. M. N., et al. J Biol Chem (1999)	p38 activates ATF-2 in repsonse to TNF stimulation, ATF-2 is a co-activator of cJUN
TSC	mTORC1	Inhibitor	Tee et al., PNAS, (2002)	Ser2448 phosphorylation in Mtor
TSC	mTORC1	Inhibitor	Inoki et al., Nat Cell Biol, (2002)	
WNT	Frizzled	Activator	Galluzzi, L., et al. Trends in Cell Biology (2018)	WNT binds frizzled receptor.
Wnt5a	ROR2	Activator	Asem, M. S. et al. Cancers (2016)	Many receptors recognise Wnt5a, including ROR family.
Wnt5a	ROR2	Activator	Dissanayake, S. K., et al. Cancer Research (2008)	ROR2 shown to be important RTK in this cell line

Supplementary Table S4. Regulatory edges in the melanoma model. Each row shows a single edge, originating from the node in the "from" column and directed to the node in the "to" column. The table also contains details of the type of edge, a brief explanation of the mechanism of this regulation and a reference to the literature where required.

Supplementary Table S5. Target functions used in the melanoma model

Node	Target Function	Reference	Explanation
4EBP1	2-var(mTORC1)	Ma and Blenis, Nature Rev MCB, (2009)	4EBP1 inhibited by mTORC1
a-MSH	0		
AIF	var(MOMP)+2*var(PAR)	Hussein, M. R., et al., J Pathology (2003)	AIF can be released either through membrane degradation (apoptosis) or by PAR (parthanatos)
AIF	var(MOMP)+2*var(PAR)	David, K. K., et al., Front Biosci (2009)	AIF can be released either through membrane degradation (apoptosis) or by PAR (parthanatos)
Akt	avg(var(PI3K),var(mTORC2))	Sarbassov et al., Science, (2005)	Activated by PI3K, and mTORC2. this dual phosphorylation is required for AKT to be activated
APC	1		
Apoptosis	generic		
ARF	floor(avg(var(cMyc),3*var(Erk))-var(TBX2)) * min(1,var(CDKN2AB))	Sherr, C. J. Nat Rev Cancer (2006)	ARF regulated by Myc, Erk and Tbx2, melanomas frequently lose ARF expression through mutation or loss of expression of CDKN2A gene.
ARF	floor(avg(var(cMyc),3*var(Erk))-var(TBX2)) * min(1,var(CDKN2AB))	Bennett, D. C. PC&MR (2008)	ARF regulated by Myc, Erk and Tbx2, melanomas frequently lose ARF expression through mutation or loss of expression of CDKN2A gene.
AXIN	2-var(Frizzled)	Galluzzi, L., et al., Trends Cell Biol (2018)	AXIN inhibited by WNT signalling through Frizzled
B-catenin	1+max(1,var(PKA))-(avg(var(APC),var(GSK3b),var(AXIN))+var(ROR2))	Galluzzi, L., et al., Trends Cell Biol (2018)	B-catenin targeted for destuction by B-catenin destruction complex (APC, AXIN and GSk3b), also stimulated by PKA and inhibited by non-canonical Wnt singalling through ROR2
B-catenin	1+max(1,var(PKA))-(avg(var(APC),var(GSK3b),var(AXIN))+var(ROR2))	O'Connell, M. P., et al. Cancer Discovery (2013)	B-catenin targeted for destuction by B-catenin destruction complex (APC, AXIN and GSk3b), also stimulated by PKA and inhibited by non-canonical Wnt singalling through ROR2
B-catenin	1+max(1,var(PKA))-(avg(var(APC),var(GSK3b),var(AXIN))+var(ROR2))	Kaur, A., et al. BJC (2016)	B-catenin targeted for destuction by B-catenin destruction complex (APC, AXIN and GSk3b), also stimulated by PKA and inhibited by non-canonical Wnt singalling through ROR2
BAD	3-(var(Akt)+var(p90RSK))	Madhunapantula, S. V., et al., Cancer Biol Ther (2011)	BAD is inhibited by pro-survival signalling through Akt and p90RSK
BAD	3-(var(Akt)+var(p90RSK))	Eisinger-Mathason et al., Steroids, (2010)	BAD is inhibited by pro-survival signalling through Akt and p90RSK
BaxBak	generic		
Bcl-2	1+max(0,(3*var(MITF)/2)-1) - ceil(avg(var(BAD),var(BIM),var(PUMA)))	Hartman, M. L., & Czyz, M. J Inv Dermatology (2015)	MITF varies between 0 and 3 and so adjustment is required, some MITF-independent expression requried, otherwise generic.
Bcl-2	1+max(0,(3*var(MITF)/2)-1) - ceil(avg(var(BAD),var(BIM),var(PUMA)))	Chen et al., Molecular Cell, (2005)	MITF varies between 0 and 3 and so adjustment is required, some MITF-independent expression requried, otherwise generic.
BCL2A1	min(1, 3*var(MITF)/2)	Haq, R. et al. PNAS (2013)	MITF required for expression of BCL2A1
BIM	3-(var(Akt)+3*var(Erk))	Madhunapantula, S. V., et al., Cancer Biol Ther (2011)	BIM is inhibited by pro-survival signalling through Akt and Erk
BIM	3-(var(Akt)+3*var(Erk))	Luciano et al., Oncogene, (2003)	BIM is inhibited by pro-survival signalling through Akt and Erk
BRAF	max(max(2*max(0,(var(PKC)/3)-1),max(2*var(HRAS)/3,2*var(NRAS)/3)),var(BRAFV600E)*(1+var(BRAFamplification)/3)/3 - 2*var(BRAFi)/3)	Burotto, M. et al. Cancer (2014)	BRAF can be activated by HRAS or NRAS or by very high level of PKC activity.
BRAF	max(max(2*max(0,(var(PKC)/3)-1),max(2*var(HRAS)/3,2*var(NRAS)/3)),var(BRAFV600E)*(1+var(BRAFamplification)/3)/3 - 2*var(BRAFi)/3)	Ueda, Y., et al., JBC (1996)	BRAF can be activated by HRAS or NRAS or by very high level of PKC activity.
BRAF	max(max(2*max(0,(var(PKC)/3)-1),max(2*var(HRAS)/3,2*var(NRAS)/3)),var(BRAFV600E)*(1+var(BRAFamplification)/3)/3 - 2*var(BRAFi)/3)	Luebker, S. A. & Koepsell, S. A. Frontiers Oncol (2019)	BRAFV600E mutation activates it and amplification of the mutant gene can enhance activity, inhibitors like vemurafenib specifically inhibit the mutant allele
BRAFamplification	0		
BRAFi	0		
BRAFV600E	1		
cAMP	generic		
Caspase-3	var(MOMP) - floor(avg(var(ML-IAP),var(IAP)))	Hussein, M. R., et al., J Pathology (2003)	Caspase-dependent apoptosis can be initiated via intrinsic pathway and is inhibited by IAPs.
CDK4	2 - avg(var(p16),var(p15))	Kim and Sharpless, Cell, (2006)	CDK4 inhibited by p16 and p15
CDK4	2 - avg(var(p16),var(p15))	Sharpless & Sherr, Nat Revs Cancer (2015)	CDK4 inhibited by p16 and p15
CDKN2AB	1		
CellCellContact	1		
cJUN	2+avg(var(CREB),var(JNK),var(p38)) - 2*max(0,3*var(MITF)/2-1)	Kappelmann, M., et al EJCB (2014)	cJUN activity promoted by p38, JNK phosphorylation and CREB, activity is mutually exclusive with MITF. MITF varies between 0 and 3 and so adjustment is required.
cJUN	2+avg(var(CREB),var(JNK),var(p38)) - 2*max(0,3*var(MITF)/2-1)	Riesenberg, S., et al. Nat Comms (2015)	cJUN activity promoted by p38, JNK phosphorylation and CREB, activity is mutually exclusive with MITF. MITF varies between 0 and 3 and so adjustment is required.
cJUN	2+avg(var(CREB),var(JNK),var(p38)) - 2*max(0,3*var(MITF)/2-1)	Zhang, H., et al. Exp Hematology Oncol (2020)	cJUN activity promoted by p38, JNK phosphorylation and CREB, activity is mutually exclusive with MITF. MITF varies between 0 and 3 and so adjustment is required.
cMyc	2*avg(var(TCF),max(0,var(Erk)*3-(var(GSK3b)-1))) - avg(var(p53),max(0,var(Smad2_3)-1))	Shaul and Seger, BBA, (2007)	Myc activated by Erk in Ras-dependent manner and it is inhibited by GSK3B in ERK-dependent way. TCF promotes expression of Myc. Myc is inhibited by p53. Myc is downregulated by high levels of TGF signalling through Smad2/3.
cMyc	2*avg(var(TCF),max(0,var(Erk)*3-(var(GSK3b)-1))) - avg(var(p53),max(0,var(Smad2_3)-1))	He, T.-C., et al., Science (1998)	Myc activated by Erk in Ras-dependent manner and it is inhibited by GSK3B in ERK-dependent way. TCF promotes expression of Myc. Myc is inhibited by p53. Myc is downregulated by high levels of TGF signalling through Smad2/3.
cMyc	2*avg(var(TCF),max(0,var(Erk)*3-(var(GSK3b)-1))) - avg(var(p53),max(0,var(Smad2_3)-1))	Ho et al., Mol Cell Bio, (2005)	Myc activated by Erk in Ras-dependent manner and it is inhibited by GSK3B in ERK-dependent way. TCF promotes expression of Myc. Myc is inhibited by p53. Myc is downregulated by high levels of TGF signalling through Smad2/3.
cMyc	2*avg(var(TCF),max(0,var(Erk)*3-(var(GSK3b)-1))) - avg(var(p53),max(0,var(Smad2_3)-1))	Warner, B.J. et al. Mol and Cell Bio (1999)	Myc activated by Erk in Ras-dependent manner and it is inhibited by GSK3B in ERK-dependent way. TCF promotes expression of Myc. Myc is inhibited by p53. Myc is downregulated by high levels of TGF signalling through Smad2/3.
CRAF	max(2*max(0,(var(PKC)/3)-1),max(2*var(HRAS)/3,2*var(NRAS)/3))	Burotto, M. et al. Cancer (2014)	CRAF can be activated by HRAS or NRAS or by very high level of PKC activity
CRAF	max(2*max(0,(var(PKC)/3)-1),max(2*var(HRAS)/3,2*var(NRAS)/3))	Ueda, Y., et al., JBC (1996)	CRAF can be activated by HRAS or NRAS or by very high level of PKC activity
CREB	max(var(PKA),max(max(0,3*var(Erk)-2), max(0,var(S6K)-1)))	Johannessen, M. & Moens, U. Front Biosci (2007)	CREB integrates signals from PKA, ERK and S6K
CycD	2*avg(var(cMyc),var(p90RSK),2*var(cJUN)) - avg(var(GSK3b),var(4EBP1))	Gartel, A. L. & Shchors, K. Exp Cell Res (2003)	Cyclin D expression is activated by p90RSK, cJUN and cMyc and it is inhibited by 4EBP1 and GSK3B. cMyc and cJun have strong effect
CycD	2*avg(var(cMyc),var(p90RSK),2*var(cJUN)) - avg(var(GSK3b),var(4EBP1))	Eisinger-Mathason et al., Steroids, (2010)	Cyclin D expression is activated by p90RSK, cJUN and cMyc and it is inhibited by 4EBP1 and GSK3B. cMyc and cJun have strong effect
CycD	2*avg(var(cMyc),var(p90RSK),2*var(cJUN)) - avg(var(GSK3b),var(4EBP1))	Lopez-Bergami, P., et al. (2007)	Cyclin D expression is activated by p90RSK, cJUN and cMyc and it is inhibited by 4EBP1 and GSK3B. cMyc and cJun have strong effect
CycD	2*avg(var(cMyc),var(p90RSK),2*var(cJUN)) - avg(var(GSK3b),var(4EBP1))	Averous et al., Oncogene, (2008)	Cyclin D expression is activated by p90RSK, cJUN and cMyc and it is inhibited by 4EBP1 and GSK3B. cMyc and cJun have strong effect
CycD_CDK4	var(CycD)*var(CDK4)/4 - avg(var(p21),var(p27))	Morgan, Nature, (1995)	CycD and CDK4 form complex, p21 and p27 inhibit formation of this complex.
CycD_CDK4	var(CycD)*var(CDK4)/4 - avg(var(p21),var(p27))	Harper et al., Cell, (1993)	CycD and CDK4 form complex, p21 and p27 inhibit formation of this complex.
CycD_CDK4	var(CycD)*var(CDK4)/4 - avg(var(p21),var(p27))	Wander et al., Clin Cancer Res, (2011)	CycD and CDK4 form complex, p21 and p27 inhibit formation of this complex.
DKK	1		
E2F	4 - (var(pRB)+var(ARF)/2)	Ha, L., et al., PNAS (2007)	E2F can be inhibited by pRB and ARF
E2F	4 - (var(pRB)+var(ARF)/2)	Weinberg, et al., Cell, (1995)	E2F can be inhibited by pRB and ARF
Erk	var(MEK)-2*var(ERKi)/3	Shaul and Seger, BBA, (2007)	Erk activated by MEK and can be inhibited by targeted inhibitors like Ulixertinib
Erk	var(MEK)-2*var(ERKi)/3	Arozarena, I. & Wellbrock, C. Ann Transl Medicine (2017)	Erk activated by MEK and can be inhibited by targeted inhibitors like Ulixertinib
ERKi	0		
FAK	generic		
FRA1	ceil(var(Erk)-1)	Hong, A. et al. Cancer Discov (2017)	High levels of ERK induce p21 arrest via Fra1 and JUNB
Frizzled	2*var(WNT)-var(DKK)	Nakamura, T., et al. J Cell Mol Med (2008)	Frizzled is receptor for Wnt, can be inhibited by DKK, but at endogenous levels Wnt signalling still occurs.
Frizzled	2*var(WNT)-var(DKK)	Spranger, S., et ak., Nature (2015)	Frizzled is receptor for Wnt, can be inhibited by DKK, but at endogenous levels Wnt signalling still occurs.
GF	1		
GF-RTK	var(GF)*var(RTK)	Easty, D. J., et al. PC&MR (2011)	Generic RTK, requires both RTK and GF to be active

GSK3b	2 - max(0, var(PKA)+3*var(Erk)*var(p90RSK) + 2*var(Akt)-3)	Manning and Toker, Cell, (2017)	GSK3b inhibited by Akt and PKA and through sequential phosphorylation by ERK and RSK
GSK3b	2 - max(0, var(PKA)+3*var(Erk)*var(p90RSK) + 2*var(Akt)-3)	Ding et al., Mol Cell, (2005)	GSK3b inhibited by Akt and PKA and through sequential phosphorylation by ERK and RSK
GSK3b	2 - max(0, var(PKA)+3*var(Erk)*var(p90RSK) + 2*var(Akt)-3)	Anjum and Blenis, Nat Rev Mol Cell Biol, (2008)	GSK3b inhibited by Akt and PKA and through sequential phosphorylation by ERK and RSK
GSK3b	2 - max(0, var(PKA)+3*var(Erk)*var(p90RSK) + 2*var(Akt)-3)	Zhang et al., Mol Cell, (2006)	GSK3b inhibited by Akt and PKA and through sequential phosphorylation by ERK and RSK
HRAS	floor(var(GF-RTK)*var(FAK)/4) + max(0,1-var(NF1)/2)	Schaller, M. D. BBA - Mol Cell Res (2001)	HRAS can be activated by GF stimulation through RTKs, in a way that depends on cell-cell contact mediated by FAK. Also constitutive activity inhibited by NF1.
HRAS	floor(var(GF-RTK)*var(FAK)/4) + max(0,1-var(NF1)/2)	Gentile, A., et al. Cancer and Metastasis Reviews (2008)	HRAS can be activated by GF stimulation through RTKs, in a way that depends on cell-cell contact mediated by FAK. Also constitutive activity inhibited by NF1.
HRAS	floor(var(GF-RTK)*var(FAK)/4) + max(0,1-var(NF1)/2)	Vigil, D., et al Nat Rev Cancer (2010)	HRAS can be activated by GF stimulation through RTKs, in a way that depends on cell-cell contact mediated by FAK. Also constitutive activity inhibited by NF1.
IAP	min(1,var(NFkB))	Wang, C.-Y., et al. Science (1998)	NF-kB activates expression of IAPs, but levels of expression capped.
Id2	max(1 - floor(3*var(MITF)/4),2-var(Smad2_3))	Schlegel, N. C. et al. PC&MR (2009)	Invasive phenotype (low MITF) resistant to TGF inhibition of Id2 via Smad2/3. MITF varies between 0 and 3 and so adjustment is required.
IKK	max(var(Akt),max(var(TNFR),3*var(Erk)))	Bai, D., et al., IJC (2009)	IKK can be activated independently by Akt, ERK or TNF signalling.
IKK	max(var(Akt),max(var(TNFR),3*var(Erk)))	Liu, J., et al. Oncogene (2007)	IKK can be activated independently by Akt, ERK or TNF signalling.
IKK	max(var(Akt),max(var(TNFR),3*var(Erk)))	Aggarwal, B. B., et al. Blood (2012)	IKK can be activated independently by Akt, ERK or TNF signalling.
Integrins	var(CellCellContact)/2	Paoli, P., et al. BBA - Mol Cell Res (2013)	Cell-Cell contact is a binary input (on or off), when on integrins are at normal activity level 1.
JNK	floor(avg(var(PKC),var(TNFR)))	López-Bergami, P., et al. Molecular Cell (2005)	JNK can be activated by PKC or TNF signalling.
JNK	floor(avg(var(PKC),var(TNFR)))	Aggarwal, B. B., et al. Blood (2012)	JNK can be activated by PKC or TNF signalling.
JUNB	ceil(var(Erk)-1)	Hong, A. et al. Cancer Discov (2017)	High levels of ERK induce p21 arrest via Fra1 and JUNB
MC1R	generic		
Mcl-1	2*var(NFkB)-ceil(avg(var(BIM),var(PUMA)))	Akgul, C. et al CMLS (2000)	Transcription of Mcl-1 controlled by NF-kB, inhibited by interaction with BIM or PUMA.
Mcl-1	2*var(NFkB)-ceil(avg(var(BIM),var(PUMA)))	Chen et al., Molecular Cell, (2005)	Transcription of Mcl-1 controlled by NF-kB, inhibited by interaction with BIM or PUMA.
Mdm	1-var(ARF)	Zhang et al., Cell, (1998)	Mdm inhibited by ARF
MEK	max(0,max(var(BRAF),var(CRAF)) - 2*var(MEKi)/3) + var(MEKGoF)/3	Burotto, M. et al. Cancer (2014)	MEK can be phosphorylated by either BRAF or CRAF
MEKGoF	0		
MEKi	0		
MITF	2		
ML-IAP	3*var(MITF)/2 - 1	Goding, C. R., & Arnheiter, H. Genes & Development (2019)	MITF varies between 0 and 3 and so adjustment is required.
MOMP	2*var(BaxBak)-(var(Bcl-2)+2*var(Mcl-1)+min(0,var(BCL2A1)-1))/2	Hussein, M. R., et al., J Pathology (2003)	BaxBak activity leads to mitochondrial membrane permeabilisation, resisted by BCL2A1, Bcl-2 and Mcl-1. Mcl-1 plays a critical role in resistance to apoptosis in melanoma.
MOMP	2*var(BaxBak)-(var(Bcl-2)+2*var(Mcl-1)+min(0,var(BCL2A1)-1))/2	Verhaegen, M. et al. Cancer Res (2006)	BaxBak activity leads to mitochondrial membrane permeabilisation, resisted by BCL2A1, Bcl-2 and Mcl-1. Mcl-1 plays a critical role in resistance to apoptosis in melanoma.
MOMP	2*var(BaxBak)-(var(Bcl-2)+2*var(Mcl-1)+min(0,var(BCL2A1)-1))/2	Vogler, M. Cell Death Differ (2012)	BaxBak activity leads to mitochondrial membrane permeabilisation, resisted by BCL2A1, Bcl-2 and Mcl-1. Mcl-1 plays a critical role in resistance to apoptosis in melanoma.
mTORC1	2-var(TSC)	Tee et al., PNAS, (2002)	Constitutive expression is inhibited by TSC
mTORC2	generic		
NF1	1		
NFkB	ceil(var(IKK)/2)*max(1,var(cJUN))	Aggarwal, B. B., et al. Blood (2012)	IKK phosphorylation is necessary for NF-kB activation. Activity can be enhanced by interaction with AP-1 (cJUN).
NFkB	ceil(var(IKK)/2)*max(1,var(cJUN))	Sabio, G., & Davis, R. J. Sem Imm (2014)	IKK phosphorylation is necessary for NF-kB activation. Activity can be enhanced by interaction with AP-1 (cJUN).
NRAS	floor(var(GF-RTK)*var(FAK)/4)	Schaller, M. D. BBA - Mol Cell Res (2001)	NRAS can be activated by GF stimulation through RTKs, in a way that depends on cell-cell contact mediated by FAK.
NRAS	floor(var(GF-RTK)*var(FAK)/4)	Gentile, A., et al. Cancer and Metastasis Reviews (2008)	NRAS can be activated by GF stimulation through RTKs, in a way that depends on cell-cell contact mediated by FAK.
p15	min(1,var(CDKN2AB))*(2*var(Smad2_3)-avg(var(Id2),var(cMyc)))	Lasfar et al. Carcinogenesis (2010)	p15 mediates TGF-B induced cell cycle arrest, but this can be prevented by activation of Myc or Id2. CDKN2AB deletions lead to loss of gene.
p15	min(1,var(CDKN2AB))*(2*var(Smad2_3)-avg(var(Id2),var(cMyc)))	Schlegel, N. C. et al. PC&MR (2009)	p15 mediates TGF-B induced cell cycle arrest, but this can be prevented by activation of Myc or Id2. CDKN2AB deletions lead to loss of gene.
p15	min(1,var(CDKN2AB))*(2*var(Smad2_3)-avg(var(Id2),var(cMyc)))	Warner, B.J. et al. Mol and Cell Bio (1999)	p15 mediates TGF-B induced cell cycle arrest, but this can be prevented by activation of Myc or Id2. CDKN2AB deletions lead to loss of gene.
p15	min(1,var(CDKN2AB))*(2*var(Smad2_3)-avg(var(Id2),var(cMyc)))	McNeal, A. S., et al. Cancer Discovery (2015)	
p16	min(1,var(CDKN2AB))* (3*var(Erk)-max(var(TCF)-1,0))	Bennett, D. C. PC&MR (2008)	p16 is produced in response to high levels of Erk activity, can be inhibited by binding of TCF to promoter. Melanomas frequently lose ARF expression through mutation or loss of expression of CDKN2A gene.
p16	min(1,var(CDKN2AB))* (3*var(Erk)-max(var(TCF)-1,0))	Delmas, V., et al. Genes & Development (2007)	p16 is produced in response to high levels of Erk activity, can be inhibited by binding of TCF to promoter. Melanomas frequently lose ARF expression through mutation or loss of expression of CDKN2A gene.
p21	avg(max(0,(3*var(MITF)/2-2))*3,var(p53)) - floor(avg(var(Akt),var(TBX2))) + 2*min(var(FRA1),var(JUNB))	Levy, C. et al. Trends in Mol Med (2006)	High levels of MITF induces p21 mediated arrest, as can p53 activation. P21 activation can be resisted by directe phosphorylation by Akt or by Tbx2 activation.
p21	avg(max(0,(3*var(MITF)/2-2))*3,var(p53)) - floor(avg(var(Akt),var(TBX2))) + 2*min(var(FRA1),var(JUNB))	El Deiry et al., Cell, (1993)	High levels of MITF induces p21 mediated arrest, as can p53 activation. P21 activation can be resisted by directe phosphorylation by Akt or by Tbx2 activation.
p21	avg(max(0,(3*var(MITF)/2-2))*3,var(p53)) - floor(avg(var(Akt),var(TBX2))) + 2*min(var(FRA1),var(JUNB))	Zhou BP et al., Nat Cell Biol, (2001)	High levels of MITF induces p21 mediated arrest, as can p53 activation. P21 activation can be resisted by directe phosphorylation by Akt or by Tbx2 activation.
p21	avg(max(0,(3*var(MITF)/2-2))*3,var(p53)) - floor(avg(var(Akt),var(TBX2))) + 2*min(var(FRA1),var(JUNB))	Bennett, D. C. PC&MR (2008)	High levels of MITF induces p21 mediated arrest, as can p53 activation. P21 activation can be resisted by directe phosphorylation by Akt or by Tbx2 activation.
p27	3-avg(3*var(cMyc),var(Akt),var(p90RSK),min(3*var(MITF)/2,2))+max(0,var(Smad2_3)-var(Akt))	Maddika et al., Drug Resistance, (2007)	p27 activated unless inhibited by cMyc, Akt, p90RSK or MITF. Of these cMyc is most important as loss of Myc alone can lead to p27 mediated arrest. P27 is also activated by TGF-B signalling, although this is specifically resisted by Akt phosphorylation. MITF inhibition maxes out at level 2.
p27	3-avg(3*var(cMyc),var(Akt),var(p90RSK),min(3*var(MITF)/2,2))+max(0,var(Smad2_3)-var(Akt))	Manning and Cantley, Cell, (2007)	p27 activated unless inhibited by cMyc, Akt, p90RSK or MITF. Of these cMyc is most important as loss of Myc alone can lead to p27 mediated arrest. P27 is also activated by TGF-B signalling, although this is specifically resisted by Akt phosphorylation. MITF inhibition maxes out at level 2.
p27	3-avg(3*var(cMyc),var(Akt),var(p90RSK),min(3*var(MITF)/2,2))+max(0,var(Smad2_3)-var(Akt))	Lasfar et al. Carcinogenesis (2010)	p27 activated unless inhibited by cMyc, Akt, p90RSK or MITF. Of these cMyc is most important as loss of Myc alone can lead to p27 mediated arrest. P27 is also activated by TGF-B signalling, although this is specifically resisted by Akt phosphorylation. MITF inhibition maxes out at level 2.
p27	3-avg(3*var(cMyc),var(Akt),var(p90RSK),min(3*var(MITF)/2,2))+max(0,var(Smad2_3)-var(Akt))	Carreira et al. Genes & Development (2006)	p27 activated unless inhibited by cMyc, Akt, p90RSK or MITF. Of these cMyc is most important as loss of Myc alone can lead to p27 mediated arrest. P27 is also activated by TGF-B signalling, although this is specifically resisted by Akt phosphorylation. MITF inhibition maxes out at level 2.
p38	generic		
p53	generic		
p90RSK	3*var(Erk)	Shaul and Seger, BBA, (2007)	Factor of 3 undoes automated recaling
PAR	var(PARP-1)-1	David, K. K., et al., Front Biosci (2009)	PARP-1 synthesises PAR, which is usually degraded by PARG
PARP-1	1+max(0,3*var(Erk)-4)	Hong, A. et al. Cancer Discov (2017)	High levels of ERK activity induce parthanatos
PI3K	max(2*var(HRAS),max(2*var(NRAS),var(GF-RTK)*var(FAK)))+max(0,1-var(PTEN))	Rodriguez-Viciana, P. et al., Nature, (1994)	PI3K can be activated by growth signalling (dependent on cell cell contact through FAK) directly or through RAS. Constitutive activation resisted by PTEN.
PI3K	max(2*var(HRAS),max(2*var(NRAS),var(GF-RTK)*var(FAK)))+max(0,1-var(PTEN))	Manning and Cantley, Cell, (2007)	PI3K can be activated by growth signalling (dependent on cell cell contact through FAK) directly or through RAS. Constitutive activation resisted by PTEN.
PI3K	max(2*var(HRAS),max(2*var(NRAS),var(GF-RTK)*var(FAK)))+max(0,1-var(PTEN))	Paoli, P., et al. BBA - Mol Cell Res (2013)	PI3K can be activated by growth signalling (dependent on cell cell contact through FAK) directly or through RAS. Constitutive activation resisted by PTEN.
PKA	var(cAMP)-max(var(Smad2_3)-1,0)	Kaur, A., et al. BJC (2016)	PKA induced by cAMP signalling, inhibited by high levels of TGF signalling.
PKA	var(cAMP)-max(var(Smad2_3)-1,0)	Pierrat, M.-J., et al. JBC (2012)	PKA induced by cAMP signalling, inhibited by high levels of TGF signalling.

PKC	max(min(1,var(GF-RTK)),var(ROR2))	Easty, D. J., et al. PC&MR (2011)	PKC stimulated by RTK signalling, including Ror2
PKC	max(min(1,var(GF-RTK)),var(ROR2))	Dissanayake, S. K., et al. JBC (2007).	PKC stimulated by RTK signalling, including Ror2
pRB	4 -(var(CycD_CDK4))	Maddika et al., Drug Resistance, (2007)	pRB inhibited by phosphorylation by CycD-Cdk4 complex.
Proliferation	generic		
PTEN	1+var(p53)-floor(var(cJUN)/2)	Hettinger et al., Cell death & Differentiation (2007)	PTEN activated by p53, constitutive expression inhibited by cJun.
PTEN	1+var(p53)-floor(var(cJUN)/2)	Stambolic et al., Mol Cell, (2001)	PTEN activated by p53, constitutive expression inhibited by cJun.
PUMA	var(p53)-3*var(Erk)/2	Villunger et al., Science, (2003)	Transciptional activation by p53 balanced by regulation by Erk through Foxo3
PUMA	var(p53)-3*var(Erk)/2	Cook et al. FEBS J (2017)	Transciptional activation by p53 balanced by regulation by Erk through Foxo3
ReplicationStress	1		
ROR2	generic		
RTK	max(1,var(cJUN)-2)	Sensi, M. et al. JID (2011)	Invasive state (MITF low) associated with increased expression of various RTKs. Otherwise, expressed at normal levels.
RTK	max(1,var(cJUN)-2)	Riesenberg, S., et al. Nat Comms (2015)	Invasive state (MITF low) associated with increased expression of various RTKs. Otherwise, expressed at normal levels.
S6K	generic		
Smad2_3	var(TBR)*var(TGF-B)	Javelaud, D., et al. PC&MR (2008)	Smad2/3 responds to TGF-B signalling through TBR receptor.
TBR	3-ceil(var(MITF))	Javelaud, D., et al. PC&MR (2011)	TBR expression lower in MITF high state.
TBR	3-ceil(var(MITF))	Sun et al. Nature (2014)	TBR expression lower in MITF high state.
TBR	3-ceil(var(MITF))	Hoek, K. S. et al. Pigm Cell Res (2006)	TBR expression lower in MITF high state.
TBX2	ceil(3*var(MITF)/8)	Bennett, D. C. PC&MR (2008)	MITF induces expression of TBX2, MITF varies between 0 and 3 and so adjustment is required.
TCF	generic		
TGF-B	floor(3*var(Erk)/2)	McNeal, A. S., et al. Cancer Discovery (2015)	MAPK activation leads to TGF-B expression (could be mediated by EGR1)
TNF-a	0		
TNFR	generic		
TSC	2 - avg(var(Akt),3*var(Erk))	Ma et al., Cell, (2005)	Consitutive TSC activity is inhibited by Akt and Erk.
TSC	2 - avg(var(Akt),3*var(Erk))	Inoki et al., Nat Cell Biol, (2002)	Consitutive TSC activity is inhibited by Akt and Erk.
WNT	1		
Wnt5a	1 - floor(3*var(MITF)/4)	O'Connell, M. P. & Weeraratna, A. T. PC&MR (2009)	Wnt5a singalling associated with low MITF state, MITF varies between 0 and 3 and so adjustment is required.

Supplementary Table S5. Target functions used in the melanoma model. The target function for each node is specified, along with a brief description of the mechanism and a reference where required. A number in the Target Function column represents nodes that are set to a constant value.

Supplementary Table S6. Experiments from the literature used to validate the melanoma model

Line Number	Paper	Cell Line	Experiment	Constraints	Expected Results	Model Result	Notes
3	Wellbrock, C., et al. Cancer Research (2004)	melan-a	Control (TPA + cAMP in media)	cAMP High, PKC High	Proliferation High	Proliferation High	
4			Control (cAMP, no TPA in media)	cAMP High	Proliferation OFF	Proliferation OFF	
5			BRAFV600E (TPA + cAMP in media)	BRAFV600E, cAMP High, PKC High, MITF High	Proliferation Very High	Proliferation Very High	Wellbrock, C., & Marais, R. JCB (2005) state that these cells become less differentiated.
6			BRAFV600E (cAMP, no TPA in media)	BRAFV600E, cAMP High, MITF High	Proliferation Very High	Proliferation Very High	
7	Wellbrock, C., & Marais, R. JCB (2005)	melan-a	Control	BRAFV600E, MITF High	Proliferation Very High	Proliferation Very High	
8			Control + MITF overexpression	BRAFV600E, MITF Very High	Proliferation High	Proliferation High	
9	Whitwam, T., et al. Oncogene (2007)	D6-MEL	Control (soft agar growth)	CellCellContact OFF	Proliferation OFF	Proliferation OFF	
10			NRASG12V (soft agar growth)	CellCellContact OFF, NRAS Mid	Proliferation Very High	Proliferation Very High	As Wellbrock, C., & Marais, R. (2005), assume MITF downregulated when MAPK activated.
11	Yang, J. et al. J Clin Invest (2010)	dox-inducible HRASG12V CDKN2A-/- mouse melanoma model	Control (TPA in media)	HRAS Mid, CDKN2AB OFF, PKC High	Apoptosis OFF	Apoptosis OFF	
12			IKK-/- mice	HRAS Mid, CDKN2AB OFF, PKC High, IKK OFF	Apoptosis Mid	Apoptosis Mid	
13			IKK-/- mice, p53 siRNA	HRAS Mid, CDKN2AB OFF, PKC High, IKK OFF, p53 OFF	Apoptosis OFF	Apoptosis OFF	
14	Swope, V. B., et al. Exp Cell Res (1995)	Normal human melanocytes	Control (normal media with bFGF and FBS)	MITF High, GF Low, ReplicationStress OFF	Proliferation OFF	Proliferation OFF	
15			Normal media + ET-1 growth factor	MITF High, GF Mid, ReplicationStress OFF	Proliferation OFF	Proliferation OFF	
16			Normal media + α-MSH	MITF High, GF Low, ReplicationStress OFF, a-MSH High	Proliferation OFF	Proliferation OFF	
17			Normal media + ET-1 + α-MSH	MITF High, GF Mid, ReplicationStress OFF, a-MSH High	Proliferation Mid	Proliferation Mid	
18	McNeal, A. S. et al. Cancer Discov (2015)	Normal human melanocytes	Naevus cell	BRAFV600E ON, MITF Very High, GF Mid, cAMP High, ReplicationStress ON	Proliferation OFF	Proliferation OFF	Presumably media contains cholera toxin or equivalent, but details of media not provided.
19			Normal melanocyte	MITF Very High, GF Mid, cAMP High	Proliferation Mid	Proliferation Mid	
20			Normal melanocyte + p15 expression	MITF Very High, GF Mid, cAMP High, p15 High	Proliferation OFF	Proliferation OFF	
21			Normal melanocyte + p16 expression	MITF Very High, GF Mid, cAMP High, p16 High	Proliferation Low	**Proliferation OFF**	
22			doxycyclin-induced BRAFV600E Cell + p15 shRNA	BRAFV600E ON, MITF Very High, GF Mid, cAMP High, p15 OFF	Proliferation Low	Proliferation Low	
23			doxycyclin-induced BRAFV600E Cell + p16 shRNA	BRAFV600E ON, MITF Very High, GF Mid, cAMP High, p16 OFF	Proliferation OFF	**Proliferation Low**	
24			Normal melanocyte + TGF-β	MITF Very High, GF Mid, cAMP High, TGF-B High	Proliferation OFF	Proliferation OFF	
25			Naevus cell (growth in tissue) + CDKR24C mutation	BRAFV600E ON, MITF Very High, GF Mid, cAMP High, ReplicationStress ON, CDK4 High	Proliferation Low	Proliferation Low	
26			Naevus cell (growth in tissue) + CDKR24C mutation + TP53R284W (dominant negative)	BRAFV600E ON, MITF Very High, GF Mid, cAMP High, ReplicationStress ON, CDK4 High, p53 OFF	Proliferation Mid	Proliferation Mid	
27	Byron, S. A., et al Mol Cancer (2012)	UACC903	Control		Proliferation Very High, Apoptosis OFF	Proliferation Very High, Apoptosis OFF	
28			MEKi (E6201)	MEKi ON	Proliferation OFF, Apoptosis Low	Proliferation OFF, Apoptosis Low	
29			PI3Ki (LY294002)	PI3K OFF	Proliferation OFF, Apoptosis Low	***Proliferation Low***, Apoptosis Low	
30			MEKi (E6201) + PI3Ki (LY294002)	MEKi ON + PI3K OFF	Proliferation OFF, Apoptosis Very High	Proliferation OFF, Apoptosis Very High	
31		WM35	Control		Proliferation Very High, Apoptosis OFF	Proliferation Very High, Apoptosis OFF	
32			MEKi (E6201)	MEKi ON	Proliferation OFF, Apoptosis High	Proliferation OFF, Apoptosis High	
33			PI3Ki (LY294002)	PI3K OFF	Proliferation OFF, Apoptosis Low	***Proliferation Low***, Apoptosis Low	
34			MEKi (E6201) + PI3Ki (LY294002)	MEKi ON + PI3K OFF	Proliferation OFF, Apoptosis Very High	Proliferation OFF, Apoptosis Very High	
35	Cheung, M., et al. Cancer Research (2008)	WM35	Control (anchorage-independent growth)	CellCellContact OFF	Apoptosis Low	Apoptosis Low	
36			BRAF siRNA (anchorage-independent growth)	CellCellContact OFF, BRAF OFF	Apoptosis Very High	Apoptosis Very High	
37			Myristoylated-Akt3 expression	CellCellContact OFF, Akt High	Apoptosis OFF	Apoptosis OFF	
38		UACC903	Control (anchorage-independent growth, no serum)	CellCellContact OFF, GF OFF	Proliferation Very High, Apoptosis OFF	Proliferation Very High, Apoptosis OFF	
39			BRAF siRNA (anchorage-independent growth, no serum)	CellCellContact OFF, GF OFF, BRAF OFF	Proliferation OFF, Apoptosis Very High	Proliferation OFF, Apoptosis Very High	
40			Akt3 siRNA (anchorage-independent growth, no serum)	CellCellContact OFF, GF OFF, Akt OFF	Proliferation OFF, Apoptosis Low	***Proliferation Low***, Apoptosis Low	
41			BRAF + Akt3 siRNA (anchorage-independent growth, no serum)	CellCellContact OFF, GF OFF, Akt OFF, BRAF OFF	Proliferation OFF, Apoptosis Very High	Proliferation OFF, Apoptosis Very High	
42	Zhuang, D. et al. Oncogene (2008)	SK-MEL-19	Control		Proliferation Very High	Proliferation Very High	Note that cellosaurus describes p53 mutation in both cell lines but Zhuang et al claim pathway is intact, p53 shRNA experiments they perform show this is not relevant to result.
43			c-Myc shRNA	cMyc OFF	Proliferation OFF	Proliferation OFF	
44			p53 shRNA	p53 OFF	Proliferation Very High	Proliferation Very High	
45			c-Myc + p53 shRNA	c-Myc + p53 shRNA	Proliferation OFF	Proliferation OFF	
46		SK-MEL-2	Control		Proliferation Very High	Proliferation Very High	
47			c-Myc shRNA	cMyc OFF	Proliferation OFF	Proliferation OFF	
48			p53 shRNA	p53 OFF	Proliferation Very High	Proliferation Very High	
49			c-Myc + p53 shRNA	c-Myc + p53 shRNA	Proliferation OFF	Proliferation OFF	
50	Solit, D. B., et al. Nature (2006)	Malme-3M	MEKi (CI-1040)	MEKi ON	Proliferation OFF, Apoptosis High	Proliferation OFF, Apoptosis High	Same control as Zhuang et al. (2008) SK-MEL-19.
51		SK-MEL-103	MEKi (CI-1040)	MEKi ON	Proliferation Low, Apoptosis Low	**Proliferation OFF**, Apoptosis Low	Same control as Zhuang et al. (2008) SK-MEL-2.
52	Shellman, Y. G., et al. JID (2000)	WM35	TGF-β	TGF-B High	Proliferation High	Proliferation High	Same control as Byron, S. A., et al Mol Cancer (2012) WM35.
53			NRASG12D expression	NRAS Mid	Proliferation Very High	Proliferation Very High	
54			TGF-β + NRASG12D expression	TGF-B High, NRAS Mid	Proliferation Very High	Proliferation Very High	
55	Donovan, J. C. H., et al. JBC (2002)	WM35	TGF-β + p27 anti-sense oligonucleotide	TGF-B High + p27 OFF	Proliferation Very High	Proliferation Very High	Same control as Shellman, Y. G., et al. JID (2000) WM35.
56	Liang, J.,et al. Nature Medicine (2002)	WM239	Control		Proliferation Very High	Proliferation Very High	
57			TGF-β	TGF-B High	Proliferation Very High	Proliferation Very High	
58			TGF-β + PTEN expression	TGF-B High, PTEN WT	Proliferation High	Proliferation High	
59	Verhaegen, M., et al. Cancer Research (2006)	SK-MEL-103	Control		Apoptosis OFF	Apoptosis OFF	
60			MEKi (U0126)	MEKi ON	Apoptosis Low	Apoptosis Low	
61			Bcl-2 shRNA	Bcl-2 OFF	Apoptosis OFF	Apoptosis OFF	
62			Mcl-1 shRNA	Mcl-1 OFF	Apoptosis Low	Apoptosis Low	
63			Bcl-2 shRNA + MEKi (U0126)	MEKi ON, Bcl-2 OFF	Apoptosis Low	Apoptosis Low	
64			Mcl-1 shRNA + MEKi (U0126)	MEKi ON, Mcl-1 OFF	Apoptosis High	Apoptosis High	
65			TW-37 (BH3 mimetic) + MEKi (U0126)	MEKi ON, Mcl-1 OFF, Bcl-2 OFF	Apoptosis High	Apoptosis High	
66			TW-37 (BH3 mimetic) + MEKi (U0126) + p53 shRNA	MEKi ON, Mcl-1 OFF, Bcl-2 OFF, p53 OFF	Apoptosis Low	**Apoptosis OFF**	
67			TW-37 (BH3 mimetic) + MEKi (U0126) + Caspase inhibitor (zVAD)	MEKi ON, Mcl-1 OFF, Bcl-2 OFF, Caspase-3 OFF	Apoptosis Mid	Apoptosis Mid	
68		Normal human melanocytes	TW-37 (BH3 mimetic) + MEKi (U0126)	ReplicationStress OFF, MITF Very High, MEKi ON, Mcl-1 OFF, Bcl-2 OFF	Apoptosis OFF	Apoptosis OFF	

69	Posch, C. et al. PNAS (2013)	d04	Control		Proliferation Very High, Apoptosis OFF	Proliferation Very High, Apoptosis OFF	
70			MEKi (JTP-74057)	MEKi ON	Proliferation OFF, Apoptosis Low	Proliferation OFF, Apoptosis Low	
71			PI3K/mTORC1/2i (GSK2126458)	PI3K OFF, mTORC1 OFF, mTORC2 OFF	Proliferation OFF, Apoptosis Low	Proliferation OFF, Apoptosis Low	
72			PI3K/mTORC1/2i (GSK2126458) + MEKi (JTP-74057)	PI3K OFF, mTORC1 OFF, mTORC2 OFF, MEKi ON	Proliferation OFF, Apoptosis Very High	Proliferation OFF, Apoptosis Very High	
73	Straussman, R., et al. Nature (2012)	SK-MEL-5	Control		Proliferation Very High, Apoptosis OFF	Proliferation Very High, Apoptosis OFF	
74			BRAFi PLX-4720	BRAFi ON	Proliferation OFF, Apoptosis High	Proliferation OFF, Apoptosis High	
75			BRAFi PLX-4720 + HGF	BRAFi ON, GF Very High	Proliferation Very High, Apoptosis OFF	Proliferation Very High, Apoptosis OFF	
76	Konieczkowski, D. J., et al. Cancer Discovery (2014)	WM266-4	Control		Proliferation Very High, Apoptosis OFF	Proliferation Very High, Apoptosis OFF	
77			ERKi (VTX11E)	ERKi ON	Proliferation OFF, Apoptosis Low	Proliferation OFF, Apoptosis Low	
78			ERKi (VTX11E) + TNF-α	ERKi ON, TNF-a High, MITF Low	Proliferation Mid, Apoptosis OFF	Proliferation Mid, Apoptosis OFF	TNF-α induces MITF low state.
79		A375	Control		Proliferation Very High, Apoptosis OFF	Proliferation Very High, Apoptosis OFF	Using MITF level determined by Muller et al 2014, not Wouters et al 2020.
80			BRAFi (PLX4720)	BRAFi ON	Proliferation OFF, Apoptosis High	Proliferation OFF, Apoptosis High	
81			MEKi (AZD2644)	MEKi ON	Proliferation OFF, Apoptosis High	Proliferation OFF, Apoptosis High	
82			ERKi (VTX11E)	ERKi ON	Proliferation OFF, Apoptosis High	Proliferation OFF, Apoptosis High	
83			AXL overexpression	RTK Very High	Proliferation Very High, Apoptosis OFF	Proliferation Very High, Apoptosis OFF	
84			BRAFi (PLX4720) + AXL overexpression	BRAFi ON, RTK Very High	Proliferation Very High, Apoptosis OFF	Proliferation Very High, Apoptosis OFF	
85			MEKi (AZD2644) + AXL overexpression	MEKi ON, RTK Very High	Proliferation Very High, Apoptosis OFF	**Proliferation OFF, Apoptosis Low**	
86			ERKi (VTX11E) + AXL overexpression	ERKi ON, RTK Very High	Proliferation OFF, Apoptosis Low	Proliferation OFF, Apoptosis Low	
87	Müller, J. et al. Nat Commun (2014)	WM266-4	BRAFi (PLX4720)	BRAFi ON	Proliferation OFF, Apoptosis Low	Proliferation OFF, Apoptosis Low	Same control as Konieczkowski, D. J., et al. Cancer Discovery (2014) WM266-4
88			BRAFi resistant clone (MITF low) + BRAFi (PLX4720)	BRAFi ON, MITF Low	Proliferation High, Apoptosis OFF	Proliferation High, Apoptosis OFF	
89			MEKi (Trametinib)	MEKi ON	Proliferation OFF, Apoptosis Low	Proliferation OFF, Apoptosis Low	
90			BRAFi resistant clone (MITF low) + MEKi (Trametinib)	MEKi ON, MITF Low	Proliferation Mid, Apoptosis OFF	Proliferation Mid, Apoptosis OFF	
91			ERKi (SCH772984)	ERKi ON	Proliferation OFF, Apoptosis Low	Proliferation OFF, Apoptosis Low	
92			BRAFi resistant clone (MITF low) + ERKi (SCH772984)	ERKi ON, MITF Low	Proliferation Mid, Apoptosis OFF	Proliferation Mid, Apoptosis OFF	
93	Shi, H., et al. Nat Commun (2012)	M395	Control		Apoptosis OFF	Apoptosis OFF	
94			BRAFi (Vemurafenib)	BRAFi ON	Apoptosis High	**Apoptosis OFF**	
95			BRAFV600E overexpression	BRAFamplification Low	Apoptosis OFF	Apoptosis OFF	
96			BRAFV600E overexpression + BRAFi (Vemurafenib)	BRAFamplification Low, BRAFi ON	Apoptosis OFF	Apoptosis OFF	
97			BRAFV600E overexpression + high BRAFi (Vemurafenib)	BRAFamplification Low, BRAFi High	Apoptosis High	**Apoptosis OFF**	
98			BRAFi resistant clone (BRAFV600E overexpression) + MEKi (AZD6244)	BRAFamplification Low, MEKi ON	Apoptosis OFF	Apoptosis OFF	
99		M249	BRAFi resistant clone (NRASQ61K mutation)	NRAS Mid	Apoptosis OFF	Apoptosis OFF	
100			BRAFi resistant clone (NRASQ61K mutation) + BRAFi (Vemurafenib)	NRAS Mid, BRAFi ON	Apoptosis OFF	Apoptosis OFF	
101			BRAFi resistant clone (NRASQ61K mutation) + MEKi (AZD6244)	NRAS Mid, MEKi ON	Apoptosis High	***Apoptosis Low***	
102	Wagle, N. et al., Cancer Discov (2014)	A375	MEK2Q60P expression + BRAFi (dabrafenib)	MEKGoF ON, BRAFi ON	Proliferation Very High, Apoptosis OFF	Proliferation Very High, Apoptosis OFF	Using MITF level determined by Muller et al 2014, not Wouters et al 2020; Same control as Konieczkowski, D. J., et al. Cancer Discovery (2014) A375
103			MEK2Q60P expression + BRAFi (dabrafenib) + MEKi (trametinib)	MEKGoF ON, BRAFi ON, MEKi ON	Proliferation Very High, Apoptosis OFF	Proliferation Very High, Apoptosis OFF	
104			MEK2Q60P expression + ERKi (VTX11E)	MEKGoF ON, ERKi ON	Proliferation OFF, Apoptosis High	**Proliferation Very High, Apoptosis OFF**	
105	Allen, E. M. V., et al., Cancer Discov (2014)	WM266-4	MITF overexpression	MITF Very High	Apoptosis OFF	Apoptosis OFF	Same control as Konieczkowski, D. J., et al. Cancer Discovery (2014) WM266-4.
106			MITF overexpression + BRAFi (dabrafenib)	MITF Very High, BRAFi ON	Apoptosis OFF	Apoptosis OFF	
107			MITF overexpression + MEKi (trametinib)	MITF Very High, MEKi ON	Apoptosis OFF	Apoptosis OFF	
108			MITF overexpression + ERKi (VTX11E)	MITF Very High, ERKi ON	Apoptosis OFF	Apoptosis OFF	
109		UACC-62	Control		Proliferation Very High, Apoptosis OFF	Proliferation Very High, Apoptosis OFF	
110			BRAFi (PLX4720)	BRAFi ON	Proliferation OFF, Apoptosis Low	Proliferation OFF, Apoptosis Low	
111			BRAFi (PLX4720) + MITF overexpression	BRAFi (PLX4720) + MITF overexpression	Proliferation Low, Apoptosis OFF	**Proliferation OFF**, Apoptosis OFF	
112	Thakur, M. D. et al. Nature (2013)	HMEX1906	Control		Proliferation Very High, Apoptosis OFF	Proliferation Very High, Apoptosis OFF	
113			BRAFi-resistant clone (BRAF amplification)	BRAFamplification Low	Proliferation Mid, Apoptosis OFF	***Proliferation Low***, Apoptosis OFF	
114			BRAFi-resistant clone (BRAF amplification) + BRAFi	BRAFamplification Low, BRAFi ON	Proliferation Very High, Apoptosis OFF	Proliferation Very High, Apoptosis OFF	
115			BRAFi-resistant clone (BRAF amplification) + high BRAFi	BRAFamplification Low, BRAFi High	Proliferation OFF, Apoptosis Low	Proliferation OFF, Apoptosis Low	
116	Moriceau, G. et al. Cancer Cell (2015)	M249	BRAFi + MEKi	BRAFi ON, MEKi ON	Proliferation OFF, Apoptosis Low	Proliferation OFF, Apoptosis Low	
117			BRAFamplification + BRAFi + MEKi	BRAFamplification Low, BRAFi ON, MEKi ON	Proliferation OFF, Apoptosis Low	Proliferation OFF, Apoptosis Low	
118			MEK1F129L + BRAFi + MEKi	MEKGoF ON, BRAFi ON, MEKi ON	Proliferation OFF, Apoptosis Low	**Proliferation Very High, Apoptosis OFF**	
119			BRAFamplification + MEK1F129L + BRAFi + MEKi	BRAFamplification Low, MEKGoF ON, BRAFi ON, MEKi ON	Proliferation Very High, Apoptosis OFF	Proliferation Very High, Apoptosis OFF	
120			BRAF ultra-amplification + BRAFi + MEKi	BRAFamplification High, BRAFi ON, MEKi ON	Proliferation Very High, Apoptosis OFF	Proliferation Very High, Apoptosis OFF	
121			BRAFi + MEKi resistant clone (BRAFamplification + MEK1F129L) without drugs	BRAFamplification Low, MEKGoF ON	Proliferation Mid, Apoptosis Mid	Proliferation Mid, Apoptosis Mid	
122			BRAFi + MEKi resistant clone (BRAF ultra-amplification) without drugs	BRAFamplification High	Proliferation Mid, Apoptosis Mid	Proliferation Mid, Apoptosis Mid	
123			BRAFi + MEKi resistant clone (BRAFamplification + MEK1F129L) + ERKi (SCH772984)	BRAFamplification Low, MEKGoF ON, ERKi ON	Proliferation Very High, Apoptosis OFF	**Proliferation Mid**, Apoptosis OFF	
124			BRAFi + MEKi resistant clone (BRAF ultra-amplification) + ERKi (SCH772984)	BRAFamplification High, ERKi ON	Proliferation Very High, Apoptosis OFF	**Proliferation Mid**, Apoptosis OFF	
125			BRAFi resistant clone (NRASQ61K) + BRAFi	NRAS Mid, BRAFi ON	Proliferation Very High, Apoptosis OFF	Proliferation Very High, Apoptosis OFF	
126			BRAFi resistant clone (BRAF NRASQ61K)	NRAS Mid	Proliferation Very High, Apoptosis OFF	Proliferation Very High, Apoptosis OFF	
127		M395	BRAFi resistant clone (BRAF amplification) + BRAFi	BRAFamplifcation Low, BRAFi ON	Proliferation Very High, Apoptosis OFF	**Proliferation Mid**, Apoptosis OFF	
128			BRAFi resistant clone (BRAF amplification)	BRAFamplifcation Low	Proliferation Very High, Apoptosis OFF	**Proliferation OFF**, Apoptosis OFF	
129		M229	BRAFi resistant clone (PDGFRβ upregulation) + BRAFi	RTK High, BRAFi ON	Proliferation Very High, Apoptosis OFF	Proliferation Very High, Apoptosis OFF	
130			BRAFi resistant clone (PDGFRβ upregulation)	RTK High	Proliferation Very High, Apoptosis OFF	Proliferation Very High, Apoptosis OFF	
131	Johannessen, C. M. et al. Nature	WM266-4	cAMP induction (Forskolin)	cAMP High, MITF Very High	Apoptosis OFF	Apoptosis OFF	Forskolin induces MITF expression.
132			cAMP induction (Forskolin) + BRAFi (PLX4720)	cAMP High, MITF Very High, BRAFi ON	Apoptosis OFF	Apoptosis OFF	
133			cAMP induction (Forskolin) + BRAFi (PLX4720) + MITF shRNA	cAMP High, MITF OFF, BRAFi ON	Apoptosis Mid	Apoptosis Mid	
134			MITF shRNA	MITF OFF	Apoptosis OFF	Apoptosis OFF	
135			BRAFi (PLX4720) + MITF shRNA	MITF OFF, BRAFi ON	Apoptosis Mid	Apoptosis Mid	

136	(2013)		BRAFi (PLX4720)	BRAFi ON	Apoptosis Low	Apoptosis Low	
137			cAMP induction (Forskolin) + BRAFi (PLX4720) + CREB dominant negative (CREBR301L)	cAMP High, BRAFi ON, CREB OFF	Apoptosis Low	Apoptosis Low	CREB required for induction of MITF (Goding, C. R. & Arnheiter, H. Gene Dev (2019)).

Supplementary Table S6. Experiments from the literature used to validate the melanoma model. For each experiment, the table shows the publication, the cell line used, the constraints applied to the model and the expected outcome, based on the experimental result. The sixth column shows the result of the model simulations, with deviations from the expected results highlighted in bold; bold results in italics are cases where the trend is correct but exact numerical value is not matched.

Supplementary Table S7. Regulatory edges in the melanoma-LC model

From	To	Type	Reference	Explanation
4EBP1	CycD	Inhibitor	Averous et al., Oncogene, (2008)	Translation inhibition
a-MSH	MC1R	Activator	Levy, C. et al. Trends in Mol Med (2006)	a-MSH binds MC1R receptor
AdhesionMolecules	Residency_LC	Activator	Deckers et al., Front in Imm (2018)	Adhesion molecules prevent migration
AIF	Apoptosis	Activator	Hussein, M. R., et al., J Pathology (2003)	AIF is effector of caspase-independent apoptosis
Akt	p27	Inhibitor	Liang, J.,et al. Nature Medicine (2002)	Thr157-p; cytoplasmic translocation from nucleus
Akt	p27	Inhibitor	Manning and Cantley, Cell, (2007)	
Akt	p21	Inhibitor	Zhou BP et al., Nat Cell Biol, (2001)	Thr 145 phosphorylation
Akt	p21	Inhibitor	Manning and Cantley, Cell, (2007)	
Akt	TSC	Inhibitor	Memmott, R. M., & Dennis, P. A. Cellular Signalling (2009)	Akt regulates mTOR through phosphorylation of TSC
Akt	GSK3b	Inhibitor	Manning and Toker, Cell, (2017)	Ser9 phosphorylation
Akt	GSK3b	Inhibitor	Cross et al. Nature (1995)	
Akt	IKK	Activator	Bai, D., et al., IJC (2009)	Akt phosphorylates IKKa at T23, required for IKK phosphorylation of p65 at S534
Akt	BAD	Inhibitor	Datta et al., Cell, (1997)	Ser136
Akt	BAD	Inhibitor	She et al., Cancer Cell, (2005)	
Akt	BIM	Inhibitor	Madhunapantula, S. V., et al., Cancer Biol Ther (2011)	Akt phopshorylates BIM
Akt_LC	mTOR_LC	Activator	Liu et al. Nat Rev Mol Cell Bio (2020)	PI3K activates mTORC1 via Akt and Tsc
APC	B-catenin	Inhibitor	Galluzzi, L., et al., Trends Cell Biol (2018)	APC, AXIN and GSK3 (and other) form B-catenin destruction complex
ARF	Mdm	Inhibitor	Zhang et al., Cell, (1998)	Proteolytic degradation
ARF	Mdm	Inhibitor	Pomerantz et al., Cell, (1998)	
ARF	E2F	Inhibitor	Ha, L., et al., PNAS (2007)	ARF causes proteosome-dependent degradation of E2F
AXIN	B-catenin	Inhibitor	Galluzzi, L., et al., Trends Cell Biol (2018)	APC, AXIN and GSK3 (and other) form B-catenin destruction complex
AXIN_LC	B-catenin_LC	Inhibitor	Zhang, Y. & Wang, X. J Hematol Oncol (2020)	Axin in complex with GSK3b, APC and CK1a inhibits B-catenin activity
Axl	PI3K_LC	Activator	Lemke et al. Nat Rev Immunology (2008)	Pi3K acts downstream of RTKs
Axl	Tyro3	Inhibitor	Bauer, T., et al. J Exp Med (2012)	Loss of Axl leads to uprgulation of Tyro3 and Mer
B-catenin	TCF	Activator	Goding, C. R., & Arnheiter, H. Genes & Development (2019)	TCF transcription factor downstream effector of B-catenin
B-catenin_LC	TCF_LC	Activator	Zhang, Y. & Wang, X. J Hematol Oncol (2020)	B-catenin acts trhough TCF transcription factor
BAD	Bcl-2	Inhibitor	Chen et al., Molecular Cell, (2005)	Bad binds Bcl-2
BaxBak	MOMP	Activator	Mohana-Kumaran et al. PC&MR (2014)	Bax and Bak cause membrane permealization of mitochondria, opposed by Bcl-2 and Mcl-1
Bcl-2	MOMP	Inhibitor	Mohana-Kumaran et al. PC&MR (2014)	Bax and Bak cause membrane permealization of mitochondria, opposed by Bcl-2 and Mcl-1
BCL2A1	MOMP	Inhibitor	Vogler, M. Cell Death Differ (2012)	BCL2A1 opposes mitochondrial membrane breakdown by BAX and BAK
BIM	Bcl-2	Inhibitor	Chen et al., Molecular Cell, (2005)	Binding BH3 domain
BIM	Mcl-1	Inhibitor	Chen et al., Molecular Cell, (2005)	Binding BH3 domain
BRAF	MEK	Activator	Burotto, M. et al. Cancer (2014)	BRAF is MAPKKK, phosphorylates MEK
BRAFamplification	BRAF	Activator	Luebker, S. A. & Koepsell, S. A. Frontiers Oncol (2019)	Amplification of BRAF gene can occur in response to targeted BRAF inhibition
BRAFi	BRAF	Inhibitor	Luebker, S. A. & Koepsell, S. A. Frontiers Oncol (2019)	Mutant BRAF inhibitors specifically inhibit BRAFV600E eg vemurafenib or dabrafenib
BRAFV600E	BRAF	Activator	Davies, H. et al. Nature (2002)	BRAFV600E mutation greatly increases activity of BRAF
cAMP	PKA	Activator	Kaur, A., et al. BJC (2016)	PKA is effector of MC1R signalling
Caspase-3	Apoptosis	Activator	Hussein, M. R., et al., J Pathology (2003)	Caspase-3 is a key effector of apoptosis
CBF-B	Id2_LC	Activator	Chopin et al., Sem Cell Dev Bio (2015)	Runx3 dimerises with CBF-B proteins
CDK4	CycD_CDK4	Activator	Morgan, Nature, (1995)	Complex formation
CDK4	CycD_CDK4	Activator	Maddika et al., Drug Resistance, (2007)	
CDKN2AB	p16	Activator	Sherr, C. J. Nat Rev Cancer (2006)	CDKN2A encodes p16 and p14/ARF
CDKN2AB	ARF	Activator	Sherr, C. J. Nat Rev Cancer (2006)	CDKN2A encodes p16 and p14/ARF
CDKN2AB	p15	Activator	Bennett, D. C. PC&MR (2016)	the CDKN2B locus is located close to CDKN2A and they are frequently lost together
CellCellContact	Integrins	Activator	Paoli, P., et al. BBA - Mol Cell Res (2013)	Cell-Cell contact is signalled through various integrins leading to FAK and Src activation
cJUN	CycD	Activator	Lopez-Bergami, P., et al. Cancer Cell (2007)	AP-1 promotes CycD expression
cJUN	PTEN	Inhibitor	Hettinger et al., Cell death & Differentiation (2007)	cJun transcriptionally inhibit PTEN at 5 upstream region of its promoter
cJUN	RTK	Activator	Sensi, M. et al. JID (2011)	Low MITF melanomas express Axl RTK
cJUN	RTK	Activator	Riesenberg, S., et al. Nat Comms (2015)	Low MITF state associated with high cJUN, PDGFR, EGFR and ERBB3
cJUN	NFkB	Activator	Riesenberg, S., et al. Nat Comms (2015)	NF-kB works synergistically with AP-1.
cJUN	NFkB	Activator	Sabio, G., & Davis, R. J. Sem Imm (2014)	AP-1 and NF-kB act synergistically in reglation of genes like TNFa
cJUN	NFkB	Activator	Riesenberg, S., et al. Nat Comms (2015)	AP-1 and NF-kB expression profiles linked in panel of melanoma cell lines
cJUN	TNF-a	Activator	Falvo, J. V., et al. Curr dir autoimm (2010)	Ets1, Sp1 and cJUN/ATF2 are involved in TNF expression
cJUN	TNF-a	Activator	Brinkman, B. M. N., et al. J Biol Chem (1999)	Ets1, Sp1 and cJUN/ATF2 are involved in TNF expression
cMyc	p27	Inhibitor	Maddika et al., Drug Resistance, (2007)	Ubiquitine-mediated degradation
cMyc	p27	Inhibitor	Yang et al., Oncogene (2001)	
cMyc	p27	Inhibitor	Obaya et al., JBC, (2002)	
cMyc	ARF	Activator	Sherr C, Genes & Dev (1998)	Transcriptional activator
cMyc	ARF	Activator	Zindy et al., Genes & Dev, (1998)	
cMyc	CycD	Activator	Mateyak et al., Mol Cel Bio, (1999)	Myc controls expression of cyclin D
cMyc	CycD	Activator	Gartel, A. L. & Shchors, K. Exp Cell Res (2003)	Myc controls expression of cyclin D
cMyc	p15	Inhibitor	Warner, B.J. et al. Mol and Cell Bio (1999)	Downregulation of Myc by TGF-B necessary for p15 induction
CRAF	MEK	Activator	Kyriakis et al., Nature (1992)	Ser218, Ser222 phosphorylation
CRAF	MEK	Activator	Burotto, M. et al. Cancer (2014)	
CREB	cJUN	Activator	Zhang, H., et al. Exp Hematology Oncol (2020)	CREB regulates expression of cJUN
CSF1	CSF1R	Activator	Wang, T. et al. Pigm Cell Melanoma R (2012)	Melanoma cells produce CSF1 (M-CSF)
CSF1	CSF1R	Activator	Hamilton, J. A. Nat Rev Immunol (2008)	CSF1 is ligand for CSF1R
CSF1R	PI3K_LC	Activator	Stanley et al., CSH Pers Bio (2014)	Pi3K acts downstream of RTKs
CycD	CycD_CDK4	Activator	Satyanarayana and Kaldis, Oncogene, (2009)	Complex formation
CycD	CycD_CDK4	Activator	Maddika et al., Drug Resistance, (2007)	
CycD_CDK4	pRB	Inhibitor	Maddika et al., Drug Resistance, (2007)	Phosphorylation of pRb
DKK	Frizzled	Inhibitor	Nakamura, T., et al. J Cell Mol Med (2008)	DKK binds Lrp5/6 preventing formation of Wnt/Frizzled/Lrp5/6 complex

DKK	Frizzled_LC	Inhibitor	Nakamura, T., et al. J Cell Mol Med (2008)	DKK binds Lrp5/6 preventing formation of Wnt/Frizzled/Lrp5/6 complex
E-cadherin	B-catenin_LC	Inhibitor	Van den Bossche et al., Blood (2012)	E-cadherin binds B-catenin inhibiting it and helping to sequester it at the plasma membrane
E-cadherin	Residency_LC	Activator	Kel et al. J Immunology (2010)	TBR KO leads to E-cadherin downregulation = increased migratory pheno of LCs
E2F	Proliferation	Activator	Johnson, Nature, (1993)	E2F required for G1/S transition
Erk	p16	Activator	Bennett, D. C. PC&MR (2008)	p16 regulation not well understood but induced by BRAF and NRAS, possibly via p38
Erk	ARF	Activator	Levine, A. J. Nat Revs Cancer (2020)	Speculative edge, based on induction ARF in response to RAS activity.
Erk	FRA1	Activator	Hong, A. et al. Cancer Discov (2017)	High levels of ERK induce p21 arrest via Fra1 and JUNB
Erk	JUNB	Activator	Hong, A. et al. Cancer Discov (2017)	High levels of ERK induce p21 arrest via Fra1 and JUNB
Erk	p90RSK	Activator	Shaul and Seger, BBA, (2007)	Thr359, Ser363 phosphorylation
Erk	p90RSK	Activator	Zhao et al., JBC (1996)	
Erk	p90RSK	Activator	Lara et al., Cancer Research, (2013)	
Erk	cMyc	Activator	Sears et al., Genes Dev, (2000)	Ser 62 phosphorylation by Erk increases half life of Myc
Erk	cMyc	Activator	Shaul and Seger, BBA, (2007)	
Erk	TSC	Inhibitor	Ma et al., Cell, (2005)	Ser664 phosphorylation
Erk	GSK3b	Inhibitor	Ding et al., Mol Cell, (2005)	Thr43 phosphorylation primes for RSK Ser9 phosphorylation
Erk	IKK	Activator	Liu, J., et al. Oncogene (2007)	MAPK pathway promotes IKK activity
Erk	CREB	Activator	Johannessen, M. & Moens, U. Front Biosci (2007)	ERK activates MAPKAPKs such as MSK1,2 and MK2 which phosphorylate CREB S133
Erk	TGF-B	Activator	McNeal, A. S., et al. Cancer Discovery (2015)	MAPK activation leads to TGF-B expression (could be mediated by EGR1)
Erk	PUMA	Inhibitor	Cook et al. FEBS J (2017)	ERK control expression of PUMA via FOXO3
Erk	BIM	Inhibitor	Luciano et al., Oncogene, (2003)	Ser69 phosphorylation
Erk	PARP-1	Activator	Hong, A. et al. Cancer Discov (2017)	High levels of ERK activity induce parthanatos
Erk	Ets1	Activator	Wasylyk, B., et al. Trends Biochem Sci (1998)	Ets1 is a target of MAPK signalling
ERK_LC	Survival_LC	Activator	Collin et al., Curr Opin Hematology (2016)	Erk activity promotes cell survival and proliferation
ERK_LC	Survival_LC	Activator	Stanley et al., CSH Pers Bio (2014)	Erk activity promotes cell survival and proliferation
ERK_LC	Proliferation_LC	Activator	Collin et al., Curr Opin Hematology (2016)	Erk activity promotes cell survival and proliferation
ERK_LC	Proliferation_LC	Activator	Stanley et al., CSH Pers Bio (2014)	Erk activity promotes cell survival and proliferation
ERKi	Erk	Inhibitor	Arozarena, I. & Wellbrock, C. Ann Transl Medicine (2017)	ERK inhibitors like VTX11E or SCH772984
Ets1	TNF-a	Activator	Falvo, J. V., et al. Curr dir autoimm (2010)	Ets1, Sp1 and cJUN/ATF2 are involved in TNF expression
Ets1	TNF-a	Activator	Tsai, E. Y. et al. Mol Cell Biol (2000)	Ets1, Sp1 and cJUN/ATF2 are involved in TNF expression
FAK	HRAS	Activator	Schaller, M. D. BBA - Mol Cell Res (2001)	FAK contributes to RAS activation via Grb2/SOS proteins
FAK	NRAS	Activator	Schaller, M. D. BBA - Mol Cell Res (2001)	FAK contributes to RAS activation via Grb2/SOS proteins
FAK	PI3K	Activator	Paoli, P., et al. BBA - Mol Cell Res (2013)	FAK activates PI3K
FRA1	p21	Activator	Hong, A. et al. Cancer Discov (2017)	High levels of ERK induce p21 arrest via Fra1 and JUNB
Frizzled	AXIN	Inhibitor	Galluzzi, L., et al., Trends Cell Biol (2018)	Binding of WNT to Frizzled receptor results in recruitment of AXIN to plasma membrane and B-catenin stabilisation
Frizzled_LC	AXIN_LC	Inhibitor	Galluzzi, L., et al., Trends Cell Biol (2018)	Binding of Wnt ligand to Fzd receptor leads to B-catenin activation via AXIN
Gas6	Axl	Activator	Collin et al., Curr Opin Hematology (2016)	Gas6 is a ligand for Axl and Tyro3
Gas6	Axl	Activator	Hieronymous et al., Sem Cell Dev Bio (2015)	Gas6 is a ligand for Axl and Tyro3
Gas6	Tyro3	Activator	Hieronymous et al., Sem Cell Dev Bio (2015)	Gas6 is a ligand for Axl and Tyro3
GF	GF-RTK	Activator	Easty, D. J., et al. PC&MR (2011)	Generic Growth Factor/Receptor Tyrosine Kinase interaction
GF-RTK	HRAS	Activator	Gentile, A., et al. Cancer and Metastasis Reviews (2008)	RAS is effector of RTK activation (can also activate PI3K directly).
GF-RTK	NRAS	Activator	Gentile, A., et al. Cancer and Metastasis Reviews (2008)	RAS is effector of RTK activation (can also activate PI3K directly).
GF-RTK	PI3K	Activator	Easty, D. J., et al. PC&MR (2011)	RTKs can activate PI3K directly through p85 subunit
GF-RTK	PKC	Activator	Easty, D. J., et al. PC&MR (2011)	RTKs activate PKC via PLCgamma
GSK3b	CycD	Inhibitor	Romero-Pozuelo et al., Cell Reports (2020)	Thr-286 phosphorylation promotes its nuclear export and cytoplasmic localization
GSK3b	CycD	Inhibitor	Liang and Slingerland, Cell cycle, (2003)	Transcriptional activator
GSK3b	cJUN	Inhibitor	Wei, W., et al. Cancer Cell (2005)	Phosphorylation of cJUN by GSK3b targets it for destruction via E3 ligase Fbw7
GSK3b	cMyc	Inhibitor	Sears et al, Genes Dev, (2000)	Thr58 phosphorylation by GSK3B when Erk mediated Ser 62 phosphorylation exists
GSK3b	B-catenin	Inhibitor	Galluzzi, L., et al., Trends Cell Biol (2018)	APC, AXIN and GSK3 (and other) form B-catenin destruction complex
HRAS	CRAF	Activator	Wee and Wang Cancers, (2017)	Ser338 and Y341 phosphorylation facilitated by RAS
HRAS	BRAF	Activator	Burotto, M. et al. Cancer (2014)	Ras activates Raf proteins
HRAS	PI3K	Activator	Rodriguez-Viciana, P. et al., Nature, (1994)	Direct interaction with catalytic subunit of PI3K
IAP	Caspase-3	Inhibitor	Mohana-Kumaran et al. PC&MR (2014)	c-IAPs inhibit caspases, via SMAC/DIABLO
Id2	p15	Inhibitor	Schlegel, N. C. et al. PC&MR (2009)	Id2 opposes TGF-B induced p15 upregulation.
Id2_LC	Residency_LC	Activator	Sere et al., Immunity (2012)	Long term LCs are Id2 dependent
IKK	NFkB	Activator	Aggarwal, B. B., et al. Blood (2012)	IKK activates NF-kB by inhbiting IkB, the repressor of NF-kB
IL-18	IL-1B	Activator	Griffiths et al. Cytokine (2005)	IL-18 acts upstream of IL-1B
IL-1B	IL-1R	Activator	Griffiths et al. Cytokine (2005)	IL-1B signals mainly through IL-1R1 receptor in LCs
IL-1R	Irf1	Activator	Orzalli et al., Molecular cell (2018)	IL-1B antiviral response if Irf1 dependent
IL-1R	TNF-a	Activator	Griffiths et al. Cytokine (2005)	IL-1B stimulates TNF production by keratinocytes
IL-34	CSF1R	Activator	Wang, Y., et al. Nat immunology (2012)	IL-34 is a ligand of CSF1R
Integrins	FAK	Activator	Paoli, P., et al. BBA - Mol Cell Res (2013)	Cell-Cell contact is signalled through various integrins leading to FAK and Src activation
Irf1	E-cadherin	Inhibitor	Jakob, T., & Udey, M. C. J immunology (1998)	TNFalpha and IL-1B signalling downregulate E-cadherin at mRNA level
Irf4	Residency_LC	Inhibitor	Sirvent et al. Nat Comms (2020)	Bulk RNA-seq
JNK	cJUN	Activator	Kappelmann, M., et al EJCB (2014)	JNK phosphorylates c-JUN at S63 and S73, activating it.
JUNB	p21	Activator	Hong, A. et al. Cancer Discov (2017)	High levels of ERK induce p21 arrest via Fra1 and JUNB
LAMTOR-p14	mTOR_LC	Activator	Collin et al., Curr Opin Hematology (2016)	LAMTOR-p14 is an adaptor complex that contributes to mTOR and ERK activation
LAMTOR-p14	ERK_LC	Activator	Collin et al., Curr Opin Hematology (2016)	LAMTOR-p14 is an adaptor complex that contributes to mTOR and ERK activation
MC1R	cAMP	Activator	Goding, C. R., & Arnheiter, H. Genes & Development (2019)	MC1R receptor stimulates CAMP signalling
Mcl-1	MOMP	Inhibitor	Mohana-Kumaran et al. PC&MR (2014)	Bax and Bak cause membrane permealization of mitochondria, opposed by Bcl-2 and Mcl-1
Mdm	p53	Inhibitor	Zhang et al., Cell, (1998)	Ubiquitine-mediated degradation

Mdm	p53	Inhibitor	Moll and Petrenko, Molecular Cancer Research, (2003)	central domain of FOXM1c functions as an RB- recruiting negative-regulatory domain
MEK	Erk	Activator	Shaul and Seger, BBA, (2007)	Thr183, Y185 phosphorylation
MEK_LC	ERK_LC	Activator	Shaul and Seger, BBA, (2007)	Thr183, Y185 phosphorylation
MEKGoF	MEK	Activator	Luebker, S. A. & Koepsell, S. A. Frontiers Oncol (2019)	Activating MEK mutations such as MEK1F129L or MEK2Q60P
MEKi	MEK	Inhibitor	Luebker, S. A. & Koepsell, S. A. Frontiers Oncol (2019)	Targeted MEK inhibitor eg trametinib
MITF	TBX2	Activator	Bennett, D. C. PC&MR (2008)	MITF induces expression of TBX2
MITF	cJUN	Inhibitor	Riesenberg, S., et al. Nat Comms (2015)	MITF binds to c-Jun enhancer
MITF	p21	Activator	Levy, C. et al. Trends in Mol Med (2006)	High levels of MITF cause differentitation and cell cycle arrest through p16 and p21
MITF	p27	Inhibitor	Carreira et al. Genes & Development (2006)	MITF destabilises p27 via Dia1
MITF	Id2	Inhibitor	Schlegel, N. C. et al. PC&MR (2009)	Invasive phenotype melanoma cells are resistant to repression of Id2 by TGF-B
MITF	TBR	Inhibitor	Hoek, K. S. et al. Pigm Cell Res (2006)	Speculative edge, low MITF state associated with enhanced TGFB signalling
MITF	TBR	Inhibitor	Javelaud, D., et al. PC&MR (2011)	Overexpression of MITF inhibits TGF-B driven induction of GLI2
MITF	TBR	Inhibitor	Sun et al. Nature (2014)	Sox10 KD (nducing MITF low state) leads to TGFBR2 expression
MITF	Wnt5a	Inhibitor	O'Connell, M. P. & Weeraratna, A. T. PC&MR (2009)	Speculative edge, Wnt5a singalling associated with MITF low state
MITF	Wnt5a	Inhibitor	Hoek, K. S. et al. Pigm Cell Res (2006)	Speculative edge, Wnt5a singalling associated with MITF low state
MITF	Wnt5a	Inhibitor	Shaffer, S. M. et al. Nature (2017)	Speculative edge, Wnt5a singalling associated with MITF low state
MITF	BCL2A1	Activator	Haq, R. et al. PNAS (2013)	MITF binds BCL2A1 promoter
MITF	ML-IAP	Activator	Goding, C. R., & Arnheiter, H. Genes & Development (2019)	MITF induces expression of ML-IAP
MITF	ML-IAP	Activator	Saladi et al. PC&MR (2013)	MITF promotes expression of ML-IAP, may also be other anti-apoptotic factors as lower in melanocytes than transformed melanoma cells
MITF	Bcl-2	Activator	Hartman, M. L., & Czyz, M. J Inv Dermatology (2015)	MITF acts as TF for Bcl-2
ML-IAP	Caspase-3	Inhibitor	Mohana-Kumaran et al. PC&MR (2014)	ML-IAP inhibits caspases, via SMAC/DIABLO
MOMP	Caspase-3	Activator	Mohana-Kumaran et al. PC&MR (2014)	Membrane breakdown causes release of cytochrome-c, formation of apoptosome and caspase activation
MOMP	AIF	Activator	Hussein, M. R., et al., J Pathology (2003)	Membrane breakdown leads to release of AIF, causing caspase-independent apoptosis
MOMP	AIF	Activator	Wang et al. Clin Cancer R (2007)	MEKi-induced apoptosis relies on caspase-independent pathway
mTOR_LC	Survival_LC	Activator	Collin et al., Curr Opin Hematology (2016)	kinase (growth/metabolism)
mTOR_LC	Proliferation_LC	Activator	Collin et al., Curr Opin Hematology (2016)	kinase (growth/metabolism)
mTORC1	S6K	Activator	Ma and Blenis, Nature Rev MCB, (2009)	Thr389 phosphorylation
mTORC1	S6K	Activator	Wee and Wang, Cancers, (2017)	
mTORC1	S6K	Activator	Isotani et al., JBC, (1999)	Thr-412 phosphorylation
mTORC1	4EBP1	Inhibitor	Ma and Blenis, Nature Rev MCB, (2009)	Thr37, Thr46 phosphorylation inhibits the 4EBP1-negative regulator of transaltion factor
mTORC1	4EBP1	Inhibitor	Brunn, Science (1997)	
mTORC1	4EBP1	Inhibitor	Wee and Wang, Cancers, (2017)	
mTORC2	Akt	Activator	Sarbassov et al., Science, (2005)	Ser473 phosphorylation
NF1	HRAS	Inhibitor	Vigil, D., et al Nat Rev Cancer (2010)	NF1 is a GAP for Ras
NF1	HRAS	Inhibitor	Maertens, et al. Cancer Discovery (2013)	effects of NF1 not mediated by NRAS in melanoma
NFkB	IAP	Activator	Wang, C.-Y., et al. Science (1998)	NF-kB activates expresion of c-IAP1 and c-IAP2
NFkB	Mcl-1	Activator	Akgul, C. et al CMLS (2000)	Mcl-1 5' region contain NF-kB binding sequence
NRAS	BRAF	Activator	Burotto, M. et al. Cancer (2014)	Ras activates Raf proteins
NRAS	PI3K	Activator	Rodriguez-Viciana, P. et al., Nature, (1994)	Direct interaction with catalytic subunit of PI3K
NRAS	CRAF	Activator	Wee and Wang, Cancers, (2017)	Ser338 and Y341 phosphorylation facilitated by RAS
p15	CDK4	Inhibitor	Sharpless & Sherr Nat Revs Cancer (2015)	p15 inhibits formation of CDK4/6 cycD complex
p16	CDK4	Inhibitor	Kim and Sharpless, Cell, (2006)	Binding
p16	CDK4	Inhibitor	Kotake et al., Anticancer Research, (2015)	
p21	CycD_CDK4	Inhibitor	Harper et al., Cell, (1993)	CDK binding functional evidence
p27	CycD_CDK4	Inhibitor	Wander et al., Clin Cancer Res, (2011)	complex formation
p38	cJUN	Activator	Brinkman, B. M. N., et al. J Biol Chem (1999)	p38 activates ATF-2 in repsonse to TNF stimulation, ATF-2 is a co-activator of cJUN
p53	p21	Activator	El Deiry et al., Cell, (1993)	Transcriptional activator
p53	PTEN	Activator	Stambolic et al., Mol Cell, (2001)	Transcriptional activator
p53	cMyc	Inhibitor	Ho et al., Mol Cell Bio, (2005)	Transcriptional repressor, changes in promoter aceylation status
p53	PUMA	Activator	Villunger et al., Science, (2003)	Transcriptional activator
p53	BaxBak	Activator	Hussein, M. R., et al., J Pathology (2003)	p53 contributes to induction of apoptosis through activation of Bax and indirectly through BH3-domain proteins
p90RSK	CycD	Activator	Eisinger-Mathason et al., Steroids, (2010)	Facilitates Transcriptional activation vi cFos and cJun
p90RSK	GSK3b	Inhibitor	Ding et al., Mol Cell, (2005)	Ser9 phosphorylation
p90RSK	GSK3b	Inhibitor	Anjum and Blenis, Nat Rev Mol Cell Biol, (2008)	
p90RSK	BAD	Inhibitor	Tan Y et al., JBC, (1999)	Ser112 phosphorylation
p90RSK	BAD	Inhibitor	Eisinger-Mathason et al., Steroids, (2010)	
PAR	AIF	Activator	David, K. K., et al., Front Biosci (2009)	PAR induces AIF release
PARP-1	PAR	Activator	David, K. K., et al., Front Biosci (2009)	PARP-1 synthesises PAR
PI3K	Akt	Activator	Stephens et al., Science, (1998)	Thr308 phosphorylation
PI3K	Akt	Activator	Alessi et al., Curr Biol. (1997)	
PI3K	Akt	Activator	Manning and Toker, Cell, (2017)	
PI3K	mTORC2	Activator	Gan et al., JBC, (2011)	Indirect assessment through AKT activation
PI3K_LC	Akt_LC	Activator	Liu et al. Nat Rev Mol Cell Bio (2020)	PI3K activates mTORC1 via Akt and Tsc
PKA	GSK3b	Inhibitor	Fang, X. et al. Proc National Acad Sci (2000)	Serine 9
PKA	B-catenin	Activator	Kaur, A., et al. BJC (2016)	PKA phosphorylates B-catenin on S675 (can also inhibit GSK3b)
PKA	CREB	Activator	Johannessen, M. & Moens, U. Front Biosci (2007)	PKA phosphorylates CREB at S133 in response to cAMP signalling
PKC	JNK	Activator	López-Bergami, P., et al. Molecular Cell (2005)	Rack1 acts as an adaptor for PKC phosphorylation
PKC	CRAF	Activator	Ueda, Y., et al., JBC (1996)	TPA activation of MEK/Erk pathway depends on CRAF but not RAS, (may affect BRAF in other cell types?)
PKC	BRAF	Activator	Ueda, Y., et al., JBC (1996)	TPA activation of MEK/Erk pathway depends on CRAF but not RAS, (may affect BRAF in other cell types?)
pRB	E2F	Inhibitor	Weinberg, et al., Cell, (1995)	Transcriptional repression
Pros1	Tyro3	Activator	Lemke et al. Nat Rev Immunology (2008)	Pros1 is a ligand for Tyro3

PTEN	PI3K	Inhibitor	Worby, C. A. & Dixon, J. E. Annu Rev Biochem (2014)	PIP3 phosphatase, acts in opposition to PI3K activity.
Pu.1	Runx3	Activator	Chopin, M., et al. J Exp Med (2013)	myeloid TF
Pu.1	Runx3	Activator	Chopin et al., Sem Cell Dev Bio (2015)	myeloid TF
Pu.1	Runx3	Activator	Deckers et al., Front in Imm (2018)	myeloid TF
PUMA	Bcl-2	Inhibitor	Chen et al., Molecular Cell, (2005)	Inhibiting BAX
PUMA	Mcl-1	Inhibitor	Chen et al., Molecular Cell, (2005)	Binding BH3 domain
ReplicationStress	p53	Activator	Mohana-Kumaran et al. PC&MR (2014)	DNA damage resulting from tumour growth leads to p53 activation via ATM, ATR etc, may also be a result of ROS
ROR2	B-catenin	Inhibitor	O'Connell, M. P., et al. Cancer Discovery (2013)	Wnt5a signalling leads to expression of Siah2 E3 ligase, which targets CTNNB1
ROR2	B-catenin	Inhibitor	Topol, L.,et al. JCB (2003)	Wnt5a signalling inhibits canonical Wnt signalling via Siah2 (not specifically ROR2 here)
ROR2	PKC	Activator	Dissanayake, S. K., et al. JBC (2007).	Wnt5a activates PKC
RTK	GF-RTK	Activator	Easty, D. J., et al. PC&MR (2011)	Generic Growth Factor/Receptor Tyrosine Kinase interaction
Runx3	Id2_LC	Activator	Collin et al., Curr Opin Hematology (2016)	TF, dimerises with CBF-B
S6K	GSK3b	Inhibitor	Zhang et al., Mol Cell, (2006)	Ser9 phosphorylation
S6K	CREB	Activator	Johannessen, M. & Moens, U. Front Biosci (2007)	p70 phosphorylates CREB in vitro, in vivo evidence less clear, could also act via SGK or Akt
Smad2_3	p27	Activator	Lasfar et al. Carcinogenesis (2010)	p27 downstream target of TGF-B causing inhibition of melanoma cells.
Smad2_3	p15	Activator	Warner, B.J. et al. Mol and Cell Bio (1999)	TGF-B induces cell cycle arrest through p15 induction
Smad2_3	p15	Activator	Lasfar et al. Carcinogenesis (2010)	p15 target of TGF-B signalling
Smad2_3	Id2	Inhibitor	Schlegel, N. C. et al. PC&MR (2009)	Proliferative phenotype melanoma cells are susceptible to repression of Id2 by TGF-B
Smad2_3	cMyc	Inhibitor	Warner, B.J. et al. Mol and Cell Bio (1999)	Downregulation of Myc by TGF-B necessary for p15 induction
Smad2_3	PKA	Inhibitor	Pierrat, M.-J., et al. JBC (2012)	TGF-B leads to repression of PKA in melanoma cells
Smad2_3_LC	Pu.1	Activator	Collin et al., Curr Opin Hematology (2016)	Downstream signal pathway of TGFb
Sp1	TNF-a	Activator	Falvo, J. V., et al. Curr dir autoimm (2010)	Ets1, Sp1 and cJUN/ATF2 are involved in TNF expression
Sp1	TNF-a	Activator	Tsai, E. Y. et al. Mol Cell Biol (2000)	Ets1, Sp1 and cJUN/ATF2 are involved in TNF expression
Sp1	TNF-a	Activator	Novak, E. M. et al. Gene Ther (2003)	Sp1 plays a role in TNF expression in melanoma cells
TBR	Smad2_3	Activator	Javelaud, D., et al. PC&MR (2008)	SMAD2/3 complex is effector of TGF-B signalling
TBR_LC	E-cadherin	Activator	Hieronymous et al., Sem Cell Dev Bio (2015)	cytokine receptor
TBR_LC	AdhesionMolecules	Activator	Yasmin et al., JID (2013)	TGFb enhances the expression of epithelial adhesion molecules
TBR_LC	Smad2_3_LC	Activator	Collin et al., Curr Opin Hematology (2016)	TGFB1 receptor complex
TBR_LC	Axl	Activator	Bauer, T., et al. J Exp Med (2012)	Western blot after using TGFbR1 receptor blockers
TBR_LC	LAMTOR-p14	Activator	Collin et al., Curr Opin Hematology (2016)	TGFB1 receptor complex
TBX2	p21	Inhibitor	Vance, K. W., et al . Cancer Research (2005)	TBX2 interacts with HDAC1, targeting it to p21 promoter
TBX2	p21	Inhibitor	Bennett, D. C. PC&MR (2008)	TBX2 is anti-senenscence factor that regulates expression of ARF and p21
TBX2	ARF	Inhibitor	Bennett, D. C. PC&MR (2008)	TBX2 is anti-senenscence factor that regulates expression of ARF and p21
TCF	p16	Inhibitor	Delmas, V., et al. Genes & Development (2007)	TCF/LEF binds p16INK4a promoter
TCF	cMyc	Activator	He, T.-C., et al., Science (1998)	TCF promotes expression of MYC gene
TCF_LC	Proliferation_LC	Activator	Becker, M. R., et al., J Invest Dermatol (2011)	Inhibitor of B-catenin (Dkk1) reduce proliferation
TGF-B	Smad2_3	Activator	Javelaud, D., et al. PC&MR (2008)	SMAD2/3 complex is effector of TGF-B signalling
TGF-B	TBR_LC	Activator	Hieronymous et al., Sem Cell Dev Bio (2015)	cytokine
TNF-a	TNFR	Activator	Aggarwal, B. B., et al. Blood (2012)	TNFR1&2 are receptors for TNF-a
TNF-a	TNFR_LC	Activator	Griffiths et al. Cytokine (2005)	TNF-a signals mainly through TNF-R2 receptor in LCs
TNFR	p38	Activator	Sabio, G., & Davis, R. J. Sem Imm (2014)	p38 activated by TNF via MKK3 and MKK6
TNFR	p38	Activator	Brinkman, B. M. N., et al. J Biol Chem (1999)	p38 activates ATF-2 in repsonse to TNF stimulation, ATF-2 is a co-activator of cJUN
TNFR	JNK	Activator	Aggarwal, B. B., et al. Blood (2012)	TNFR leads to activation of JNK (and p38,Erk) via TRAF2 ,MEKK1 and MKK7
TNFR	IKK	Activator	Aggarwal, B. B., et al. Blood (2012)	TNF induces IKK activation through TRAF2
TNFR_LC	Irf1	Activator	Davies et al. Front Imm (2021)	Single cell RNA-seq
TSC	mTORC1	Inhibitor	Tee et al., PNAS, (2002)	Ser2448 phosphorylation in Mtor
TSC	mTORC1	Inhibitor	Inoki et al., Nat Cell Biol, (2002)	
Tyro3	PI3K_LC	Activator	Lemke et al. Nat Rev Immunology (2008)	Pi3K acts downstream of RTKs
WNT	Frizzled	Activator	Galluzzi, L., et al., Trends Cell Biol (2018)	WNT binds frizzled receptor.
WNT	Frizzled_LC	Activator	Galluzzi, L., et al., Trends Cell Biol (2018)	Binding of Wnt ligand to Fzd receptor leads to B-catenin activation
Wnt5a	ROR2	Activator	Asem, M. S. et al. Cancers (2016)	Many receptors recognise Wnt5a, including ROR family.
Wnt5a	ROR2	Activator	Dissanayake, S. K., et al. Cancer Research (2008)	ROR2 shown to be important RTK in this cell line

Supplementary Table S7. Regulatory edges in the melanoma-LC model. Each row shows a single edge, originating from the node in the "from" column and directed to the node in the "to" column. The table also contains details of the type of edge, a brief explanation of the mechanism of this regulation and a reference to the literature where required.

Supplementary Table S8. Target functions used in the melanoma-LC model

Node	Target Function	Reference	Explanation
4EBP1	2-var(mTORC1)	Ma and Blenis, Nature Rev MCB, (2009)	4EBP1 inhibited by mTORC1
a-MSH	0		
AdhesionMolecules	generic		
AIF	var(MOMP)+2*var(PAR)	Hussein, M. R., et al., J Pathology (2003)	AIF can be released either through membrane degradation (apoptosis) or by PAR (parthanatos)
AIF	var(MOMP)+2*var(PAR)	David, K. K., et al., Front Biosci (2009)	AIF can be released either through membrane degradation (apoptosis) or by PAR (parthanatos)
Akt	avg(var(PI3K),var(mTORC2))	Sarbassov et al., Science, (2005)	Activated by PI3K, and mTORC2. this dual phosphorylation is required for AKT to be activated
Akt_LC	generic		
APC	1		
Apoptosis	generic		
ARF	floor(avg(var(cMyc),3*var(Erk))-var(TBX2)) * min(1,var(CDKN2AB))	Sherr, C. J. Nat Rev Cancer (2006)	ARF regulated by Myc, Erk and Tbx2, melanomas frequently lose ARF expression through mutation or loss of expression of CDKN2A gene.
ARF	floor(avg(var(cMyc),3*var(Erk))-var(TBX2)) * min(1,var(CDKN2AB))	Bennett, D. C. PC&MR (2008)	ARF regulated by Myc, Erk and Tbx2, melanomas frequently lose ARF expression through mutation or loss of expression of CDKN2A gene.
AXIN	2-var(Frizzled)	Galluzzi, L., et al., Trends Cell Biol (2018)	AXIN inhibited by WNT signalling through Frizzled
AXIN_LC	2-var(Frizzled_LC)	Galluzzi, L., et al., Trends Cell Biol (2018)	Binding of Wnt ligand to Fzd receptor leads to B-catenin activation via AXIN
Axl	var(Gas6)*var(TBR_LC)	Collin et al., Curr Opin Hematology (2016)	Axl is induced by TGF signalling, Gas6 is ligand for Axl
Axl	var(Gas6)*var(TBR_LC)	Bauer, T., et al. J Exp Med (2012)	Axl is induced by TGF signalling, Gas6 is ligand for Axl
B-catenin	1+max(1,var(PKA))-(avg(var(APC),var(GSK3b),var(AXIN))+var(ROR2))	Galluzzi, L., et al., Trends Cell Biol (2018)	B-catenin targeted for destuction by B-catenin destruction complex (APC, AXIN and GSk3b), also stimulated by PKA and inhibited by non-canonical Wnt singalling through ROR2
B-catenin	1+max(1,var(PKA))-(avg(var(APC),var(GSK3b),var(AXIN))+var(ROR2))	O'Connell, M. P., et al. Cancer Discovery (2013)	B-catenin targeted for destuction by B-catenin destruction complex (APC, AXIN and GSk3b), also stimulated by PKA and inhibited by non-canonical Wnt singalling through ROR2
B-catenin	1+max(1,var(PKA))-(avg(var(APC),var(GSK3b),var(AXIN))+var(ROR2))	Kaur, A., et al. BJC (2016)	B-catenin targeted for destuction by B-catenin destruction complex (APC, AXIN and GSk3b), also stimulated by PKA and inhibited by non-canonical Wnt singalling through ROR2
B-catenin_LC	3-var(AXIN_LC)-var(E-cadherin)	Zhang, Y. & Wang, X. J Hematol Oncol (2020)	Axin in complex with GSK3b, APC and CK1a inhibits B-catenin activity
B-catenin_LC	3-var(AXIN_LC)-var(E-cadherin)	Van den Bossche et al., Blood (2012)	E-cadherin binds B-catenin inhibiting it and helping to sequester it at the plasma membrane
BAD	3-(var(Akt)+var(p90RSK))	Madhunapantula, S. V., et al., Cancer Biol Ther (2011)	BAD is inhibited by pro-survival signalling through Akt and p90RSK
BAD	3-(var(Akt)+var(p90RSK))	Eisinger-Mathason et al., Steroids, (2010)	BAD is inhibited by pro-survival signalling through Akt and p90RSK
BaxBak	generic		
Bcl-2	1+max(0,(3*var(MITF)/2)-1) - ceil(avg(var(BAD),var(BIM),var(PUMA)))	Hartman, M. L., & Czyz, M. J Inv Dermatology (2015)	MITF varies between 0 and 3 and so adjustment is required, some MITF-independent expression requried, otherwise generic.
Bcl-2	1+max(0,(3*var(MITF)/2)-1) - ceil(avg(var(BAD),var(BIM),var(PUMA)))	Chen et al., Molecular Cell, (2005)	MITF varies between 0 and 3 and so adjustment is required, some MITF-independent expression requried, otherwise generic.
BCL2A1	min(1, 3*var(MITF)/2)	Haq, R. et al. PNAS (2013)	MITF required for expression of BCL2A1
BIM	3-(var(Akt)+3*var(Erk))	Madhunapantula, S. V., et al., Cancer Biol Ther (2011)	BIM is inhibited by pro-survival signalling through Akt and Erk
BIM	3-(var(Akt)+3*var(Erk))	Luciano et al., Oncogene, (2003)	BIM is inhibited by pro-survival signalling through Akt and Erk
BRAF	max(max(2*max(0,(var(PKC)/3)-1),max(2*var(HRAS)/3,2*var(NRAS)/3)),var(BRAFV600E)*(1+var(BRAFamplification)/3)/3 - 2*var(BRAFi)/3)	Burotto, M. et al. Cancer (2014)	BRAF can be activated by HRAS or NRAS or by very high level of PKC activity.
BRAF	max(max(2*max(0,(var(PKC)/3)-1),max(2*var(HRAS)/3,2*var(NRAS)/3)),var(BRAFV600E)*(1+var(BRAFamplification)/3)/3 - 2*var(BRAFi)/3)	Ueda, Y., et al., JBC (1996)	BRAF can be activated by HRAS or NRAS or by very high level of PKC activity.
BRAF	max(max(2*max(0,(var(PKC)/3)-1),max(2*var(HRAS)/3,2*var(NRAS)/3)),var(BRAFV600E)*(1+var(BRAFamplification)/3)/3 - 2*var(BRAFi)/3)	Luebker, S. A. & Koepsell, S. A. Frontiers Oncol (2019)	BRAFV600E mutation activates it and amplification of the mutant gene can enhance activity, inhibitors like vemurafenib specifically inhibit the mutant allele
BRAFamplification	0		
BRAFi	0		
BRAFV600E	0		
cAMP	generic		
Caspase-3	var(MOMP) - floor(avg(var(ML-IAP),var(IAP)))	Hussein, M. R., et al., J Pathology (2003)	Caspase-dependent apoptosis can be initiated via intrinsic pathway and is inhibited by IAPs.
CBF-B	1		
CDK4	2 - avg(var(p16),var(p15))	Kim and Sharpless, Cell, (2006)	CDK4 inhibited by p16 and p15
CDK4	2 - avg(var(p16),var(p15))	Sharpless & Sherr Nat Revs Cancer (2015)	CDK4 inhibited by p16 and p15
CDKN2AB	1		
CellCellContact	1		
cJUN	2+avg(var(CREB),var(JNK),var(p38)) - 2*max(0,3*var(MITF)/2-1)	Kappelmann, M., et al EJCB (2014)	cJUN activity promoted by p38, JNK phosphorylation and CREB, activity is mutually exclusive with MITF. MITF varies between 0 and 3 and so adjustment is required.
cJUN	2+avg(var(CREB),var(JNK),var(p38)) - 2*max(0,3*var(MITF)/2-1)	Riesenberg, S., et al. Nat Comms (2015)	cJUN activity promoted by p38, JNK phosphorylation and CREB, activity is mutually exclusive with MITF. MITF varies between 0 and 3 and so adjustment is required.
cJUN	2+avg(var(CREB),var(JNK),var(p38)) - 2*max(0,3*var(MITF)/2-1)	Zhang, H., et al. Exp Hematology Oncol (2020)	cJUN activity promoted by p38, JNK phosphorylation and CREB, activity is mutually exclusive with MITF. MITF varies between 0 and 3 and so adjustment is required.
cMyc	2*avg(var(TCF),max(0,var(Erk)*3-(var(GSK3b)-1))) - avg(var(p53),max(0,var(Smad2_3)-1))	Shaul and Seger, BBA, (2007)	Myc activated by Erk in Ras-dependent manner and it is inhibited by GSK3B in ERK-dependent way. TCF promotes expression of Myc. Myc is inhibited by p53. Myc is downregulated by high levels of TGF signalling through Smad2/3.
cMyc	2*avg(var(TCF),max(0,var(Erk)*3-(var(GSK3b)-1))) - avg(var(p53),max(0,var(Smad2_3)-1))	He, T.-C., et al., Science (1998)	Myc activated by Erk in Ras-dependent manner and it is inhibited by GSK3B in ERK-dependent way. TCF promotes expression of Myc. Myc is inhibited by p53. Myc is downregulated by high levels of TGF signalling through Smad2/3.
cMyc	2*avg(var(TCF),max(0,var(Erk)*3-(var(GSK3b)-1))) - avg(var(p53),max(0,var(Smad2_3)-1))	Ho et al., Mol Cell Bio, (2005)	Myc activated by Erk in Ras-dependent manner and it is inhibited by GSK3B in ERK-dependent way. TCF promotes expression of Myc. Myc is inhibited by p53. Myc is downregulated by high levels of TGF signalling through Smad2/3.
cMyc	2*avg(var(TCF),max(0,var(Erk)*3-(var(GSK3b)-1))) - avg(var(p53),max(0,var(Smad2_3)-1))	Warner, B.J. et al. Mol and Cell Bio (1999)	Myc activated by Erk in Ras-dependent manner and it is inhibited by GSK3B in ERK-dependent way. TCF promotes expression of Myc. Myc is inhibited by p53. Myc is downregulated by high levels of TGF signalling through Smad2/3.
CRAF	max(2*max(0,(var(PKC)/3)-1),max(2*var(HRAS)/3,2*var(NRAS)/3))	Burotto, M. et al. Cancer (2014)	CRAF can be activated by HRAS or NRAS or by very high level of PKC activity
CRAF	max(2*max(0,(var(PKC)/3)-1),max(2*var(HRAS)/3,2*var(NRAS)/3))	Ueda, Y., et al., JBC (1996)	CRAF can be activated by HRAS or NRAS or by very high level of PKC activity
CREB	max(var(PKA),max(max(0,3*var(Erk)-2), max(0,var(S6K)-1)))	Johannessen, M. & Moens, U. Front Biosci (2007)	CREB integrates signals from PKA, ERK and S6K
CSF1	0		
CSF1R	max(var(IL-34), var(CSF1))	Hamilton, J. A. Nat Rev Immunol (2008)	CSF1R is receptor for both IL-34 and CSF1
CycD	2*avg(var(cMyc),var(p90RSK),2*var(cJUN)) - avg(var(GSK3b),var(4EBP1))	Gartel, A. L. & Shchors, K. Exp Cell Res (2003)	Cyclin D expression is activated by p90RSK, cJUN and cMyc and it is inhibited by 4EBP1 and GSK3B. cMyc and cJun have strong effect
CycD	2*avg(var(cMyc),var(p90RSK),2*var(cJUN)) - avg(var(GSK3b),var(4EBP1))	Eisinger-Mathason et al., Steroids, (2010)	Cyclin D expression is activated by p90RSK, cJUN and cMyc and it is inhibited by 4EBP1 and GSK3B. cMyc and cJun have strong effect
CycD	2*avg(var(cMyc),var(p90RSK),2*var(cJUN)) - avg(var(GSK3b),var(4EBP1))	Lopez-Bergami, P., et al. Cancer Cell (2007)	Cyclin D expression is activated by p90RSK, cJUN and cMyc and it is inhibited by 4EBP1 and GSK3B. cMyc and cJun have strong effect
CycD	2*avg(var(cMyc),var(p90RSK),2*var(cJUN)) - avg(var(GSK3b),var(4EBP1))	Averous et al., Oncogene, (2008)	Cyclin D expression is activated by p90RSK, cJUN and cMyc and it is inhibited by 4EBP1 and GSK3B. cMyc and cJun have strong effect
CycD_CDK4	var(CycD)*var(CDK4)/4 - avg(var(p21),var(p27))	Morgan, Nature, (1995)	CycD and CDK4 form complex, p21 and p27 inhibit formation of this complex.
CycD_CDK4	var(CycD)*var(CDK4)/4 - avg(var(p21),var(p27))	Harper et al., Cell, (1993)	CycD and CDK4 form complex, p21 and p27 inhibit formation of this complex.
CycD_CDK4	var(CycD)*var(CDK4)/4 - avg(var(p21),var(p27))	Wander et al., Clin Cancer Res, (2011)	CycD and CDK4 form complex, p21 and p27 inhibit formation of this complex.
DKK	1		
DKK	1		
E-cadherin	generic		
E2F	4 - (var(pRB)+var(ARF)/2)	Ha, L., et al., PNAS (2007)	E2F can be inhibited by pRB and ARF

E2F	4 - (var(pRB)+var(ARF)/2)	Weinberg, et al., Cell, (1995)	E2F can be inhibited by pRB and ARF
Erk	var(MEK)-2*var(ERKi)/3	Shaul and Seger, BBA, (2007)	Erk activated by MEK and can be inhibited by targeted inhibitors like Ulixertinib
Erk	var(MEK)-2*var(ERKi)/3	Arozarena, I. & Wellbrock, C. Ann Transl Medicine (2017)	Erk activated by MEK and can be inhibited by targeted inhibitors like Ulixertinib
ERK_LC	var(LAMTOR-p14)*var(MEK_LC)	Nada, S. et al. Embo J (2009)	LAMTOR complex facilitates MEK-ERK interaction
ERKi	0		
Ets1	3*var(Erk)/2	Wasylyk, B., et al. Trends Biochem Sci (1998)	Scaled ERK
FAK	generic		
FRA1	ceil(var(Erk)-1)	Hong, A. et al. Cancer Discov (2017)	High levels of ERK induce p21 arrest via Fra1 and JUNB
Frizzled	2*var(WNT)-var(DKK)	Nakamura, T., et al. J Cell Mol Med (2008)	Frizzled is receptor for Wnt, can be inhibited by DKK, but at endogenous levels Wnt signalling still occurs.
Frizzled	2*var(WNT)-var(DKK)	Spranger, S., et ak., Nature (2015)	Frizzled is receptor for Wnt, can be inhibited by DKK, but at endogenous levels Wnt signalling still occurs.
Frizzled_LC	2*var(WNT)-var(DKK)	Nakamura, T., et al. J Cell Mol Med (2008)	Frizzled is receptor for Wnt, can be inhibited by DKK, but at endogenous levels Wnt signalling still occurs.
Frizzled_LC	2*var(WNT)-var(DKK)	Becker, M. R., et al., J Invest Dermatol (2011)	Frizzled is receptor for Wnt, can be inhibited by DKK, but at endogenous levels Wnt signalling still occurs.
Gas6	1		
GF	1		
GF-RTK	var(GF)*var(RTK)	Easty, D. J., et al. PC&MR (2011)	Generic RTK, requires both RTK and GF to be active
GSK3b	2 - max(0, var(PKA)+3*var(Erk)*var(p90RSK) + 2*var(Akt)-3)	Manning and Toker, Cell, (2017)	GSK3b inhibited by Akt and PKA and through sequential phosphorylation by ERK and RSK
GSK3b	2 - max(0, var(PKA)+3*var(Erk)*var(p90RSK) + 2*var(Akt)-3)	Ding et al., Mol Cell, (2005)	GSK3b inhibited by Akt and PKA and through sequential phosphorylation by ERK and RSK
GSK3b	2 - max(0, var(PKA)+3*var(Erk)*var(p90RSK) + 2*var(Akt)-3)	Anjum and Blenis, Nat Rev Mol Cell Biol, (2008)	GSK3b inhibited by Akt and PKA and through sequential phosphorylation by ERK and RSK
GSK3b	2 - max(0, var(PKA)+3*var(Erk)*var(p90RSK) + 2*var(Akt)-3)	Zhang et al., Mol Cell, (2006)	GSK3b inhibited by Akt and PKA and through sequential phosphorylation by ERK and RSK
HRAS	floor(var(GF-RTK)*var(FAK)/4) + max(0,1-var(NF1)/2)	Schaller, M. D. BBA - Mol Cell Res (2001)	HRAS can be activated by GF stimulation through RTKs, in a way that depends on cell-cell contact mediated by FAK. Also constitutive activity inhibited by NF1.
HRAS	floor(var(GF-RTK)*var(FAK)/4) + max(0,1-var(NF1)/2)	Gentile, A., et al. Cancer and Metastasis Reviews (2008)	HRAS can be activated by GF stimulation through RTKs, in a way that depends on cell-cell contact mediated by FAK. Also constitutive activity inhibited by NF1.
HRAS	floor(var(GF-RTK)*var(FAK)/4) + max(0,1-var(NF1)/2)	Vigil, D., et al Nat Rev Cancer (2010)	HRAS can be activated by GF stimulation through RTKs, in a way that depends on cell-cell contact mediated by FAK. Also constitutive activity inhibited by NF1.
IAP	min(1,var(NFkB))	Wang, C.-Y., et al. Science (1998)	NF-kB activates expression of IAPs, but levels of expression capped.
Id2	max(1 - floor(3*var(MITF)/4),2-var(Smad2_3))	Schlegel, N. C. et al. PC&MR (2009)	Invasive phenotype (low MITF) resistant to TGF inhibition of Id2 via Smad2/3. MITF varies between 0 and 3 and so adjustment is required.
Id2_LC	var(CBF-B)*var(Runx3)	Collin et al., Curr Opin Hematology (2016)	Runx3 dimerise with CBF-B
IKK	max(var(Akt),max(var(TNFR),3*var(Erk)))	Bai, D., et al., IJC (2009)	IKK can be activated independently by Akt, ERK or TNF signalling.
IKK	max(var(Akt),max(var(TNFR),3*var(Erk)))	Liu, J., et al. Oncogene (2007)	IKK can be activated independently by Akt, ERK or TNF signalling.
IKK	max(var(Akt),max(var(TNFR),3*var(Erk)))	Aggarwal, B. B., et al. Blood (2012)	IKK can be activated independently by Akt, ERK or TNF signalling.
IL-18	0		
IL-1B	1+var(IL-18)	Griffiths et al. Cytokine (2005)	IL-1B consitutively present in epidermis, increased levels trigger LC migration
IL-1R	generic		
IL-34	1		
Integrins	var(CellCellContact)/2	Paoli, P., et al. BBA - Mol Cell Res (2013)	Cell-Cell contact is a binary input (on or off), when on integrins are at normal activity level 1.
Irf1	var(TNFR_LC)*var(IL-1R)	Griffiths et al. Cytokine (2005)	Combination of 2 cytokine signals are needed to induce LC migration
Irf4	1		
JNK	floor(avg(var(PKC),var(TNFR)))	López-Bergami, P., et al. Molecular Cell (2005)	JNK can be activated by PKC or TNF signalling.
JNK	floor(avg(var(PKC),var(TNFR)))	Aggarwal, B. B., et al. Blood (2012)	JNK can be activated by PKC or TNF signalling.
JUNB	ceil(var(Erk)-1)	Hong, A. et al. Cancer Discov (2017)	High levels of ERK induce p21 arrest via Fra1 and JUNB
LAMTOR-p14	1+var(TBR_LC)	Collin et al., Curr Opin Hematology (2016)	LAMTOR complex responds to TGF signalling
MC1R	generic		
Mcl-1	2*var(NFkB)-ceil(avg(var(BIM),var(PUMA)))	Akgul, C. et al CMLS (2000)	Transcription of Mcl-1 controlled by NF-kB, inhibited by interaction with BIM or PUMA.
Mcl-1	2*var(NFkB)-ceil(avg(var(BIM),var(PUMA)))	Chen et al., Molecular Cell, (2005)	Transcription of Mcl-1 controlled by NF-kB, inhibited by interaction with BIM or PUMA.
Mdm	1-var(ARF)	Zhang et al., Cell, (1998)	Mdm inhibited by ARF
MEK	max(0,max(var(BRAF),var(CRAF)) - 2*var(MEKi)/3) + var(MEKGoF)/3	Burotto, M. et al. Cancer (2014)	MEK can be phosphorylated by either BRAF or CRAF
MEK_LC	1		
MEKGoF	0		
MEKi	0		
MITF	2		
ML-IAP	3*var(MITF)/2 - 1	Goding, C. R., & Arnheiter, H. Genes & Development (2019)	MITF varies between 0 and 3 and so adjustment is required.
MOMP	2*var(BaxBak)-(var(Bcl-2)+2*var(Mcl-1)+min(0,var(BCL2A1)-1))/2	Hussein, M. R., et al., J Pathology (2003)	BaxBak activity leads to mitochondrial membrane permeabilisation, resisted by BCL2A1, Bcl-2 and Mcl-1. Mcl-1 plays a critical role in resistance to apoptosis in melanoma.
MOMP	2*var(BaxBak)-(var(Bcl-2)+2*var(Mcl-1)+min(0,var(BCL2A1)-1))/2	Verhaegen, M. et al. Cancer Res (2006)	BaxBak activity leads to mitochondrial membrane permeabilisation, resisted by BCL2A1, Bcl-2 and Mcl-1. Mcl-1 plays a critical role in resistance to apoptosis in melanoma.
MOMP	2*var(BaxBak)-(var(Bcl-2)+2*var(Mcl-1)+min(0,var(BCL2A1)-1))/2	Vogler, M. Cell Death Differ (2012)	BaxBak activity leads to mitochondrial membrane permeabilisation, resisted by BCL2A1, Bcl-2 and Mcl-1. Mcl-1 plays a critical role in resistance to apoptosis in melanoma.
mTOR_LC	min(var(LAMTOR-p14),var(Akt_LC))	Collin et al., Curr Opin Hematology (2016)	LAMTOR complex acts as adaptor to aid in mTOR activation, which is also mediated by PI3K phosphorylation
mTOR_LC	min(var(LAMTOR-p14),var(Akt_LC))	Liu et al. Nat Rev Mol Cell Bio (2020)	LAMTOR complex acts as adaptor to aid in mTOR activation, which is also mediated by PI3K phosphorylation
mTOR_LC	min(var(LAMTOR-p14),var(Akt_LC))	Laplante, M. & Sabatini, D. Cell (2012)	Functional AND gate between TSC and LAMTOR dependent mechanisms of mTOR activation
mTORC1	2-var(TSC)	Tee et al., PNAS, (2002)	Constitutive expression is inhibited by TSC
mTORC2	generic		
NF1	1		
NFkB	ceil(var(IKK)/2)*max(1,var(cJUN))	Aggarwal, B. B., et al. Blood (2012)	IKK phosphorylation is necessary for NF-kB activation. Activity can be enhanced by interaction with AP-1 (cJUN).
NFkB	ceil(var(IKK)/2)*max(1,var(cJUN))	Sabio, G., & Davis, R. J. Sem Imm (2014)	IKK phosphorylation is necessary for NF-kB activation. Activity can be enhanced by interaction with AP-1 (cJUN).
NRAS	floor(var(GF-RTK)*var(FAK)/4)	Schaller, M. D. BBA - Mol Cell Res (2001)	NRAS can be activated by GF stimulation through RTKs, in a way that depends on cell-cell contact mediated by FAK.
NRAS	floor(var(GF-RTK)*var(FAK)/4)	Gentile, A., et al. Cancer and Metastasis Reviews (2008)	NRAS can be activated by GF stimulation through RTKs, in a way that depends on cell-cell contact mediated by FAK.
p15	min(1,var(CDKN2AB))*(2*var(Smad2_3)-avg(var(Id2),var(cMyc)))	Lasfar et al. Carcinogenesis (2010)	p15 mediates TGF-B induced cell cycle arrest, but this can be prevented by activation of Myc or Id2. CDKN2AB deletions lead to loss of gene.
p15	min(1,var(CDKN2AB))*(2*var(Smad2_3)-avg(var(Id2),var(cMyc)))	Schlegel, N. C. et al. PC&MR (2009)	p15 mediates TGF-B induced cell cycle arrest, but this can be prevented by activation of Myc or Id2. CDKN2AB deletions lead to loss of gene.
p15	min(1,var(CDKN2AB))*(2*var(Smad2_3)-avg(var(Id2),var(cMyc)))	Warner, B.J. et al. Mol and Cell Bio (1999)	p15 mediates TGF-B induced cell cycle arrest, but this can be prevented by activation of Myc or Id2. CDKN2AB deletions lead to loss of gene.
p15	min(1,var(CDKN2AB))*(2*var(Smad2_3)-avg(var(Id2),var(cMyc)))	McNeal, A. S., et al. Cancer Discovery (2015)	
p16	min(1,var(CDKN2AB))* (3*var(Erk)-max(var(TCF)-1,0))	Bennett, D. C. PC&MR (2008)	p16 is produced in response to high levels of Erk activity, can be inhibited by binding of TCF to promoter. Melanomas frequently lose ARF expression through mutation or loss of expression of CDKN2A gene.
p16	min(1,var(CDKN2AB))* (3*var(Erk)-max(var(TCF)-1,0))	Delmas, V., et al. Genes & Development (2007)	p16 is produced in response to high levels of Erk activity, can be inhibited by binding of TCF to promoter. Melanomas frequently lose ARF expression through mutation or loss of expression of CDKN2A gene.
p21	avg(max(0,(3*var(MITF)/2-2))*3,var(p53)) - floor(avg(var(Akt),var(TBX2))) + 2*min(var(FRA1),var(JUNB))	Levy, C. et al. Trends in Mol Med (2006)	High levels of MITF induces p21 mediated arrest, as can p53 activation. P21 activation can be resisted by directe phosphorylation by Akt or by Tbx2 activation.

p21	avg(max(0,(3*var(MITF)/2-2))*3,var(p53)) - floor(avg(var(Akt),var(TBX2))) + 2*min(var(FRA1),var(JUNB))	El Deiry et al., Cell, (1993)	High levels of MITF induces p21 mediated arrest, as can p53 activation. P21 activation can be resisted by directe phosphorylation by Akt or by Tbx2 activation.
p21	avg(max(0,(3*var(MITF)/2-2))*3,var(p53)) - floor(avg(var(Akt),var(TBX2))) + 2*min(var(FRA1),var(JUNB))	Zhou BP et al., Nat Cell Biol, (2001)	High levels of MITF induces p21 mediated arrest, as can p53 activation. P21 activation can be resisted by directe phosphorylation by Akt or by Tbx2 activation.
p21	avg(max(0,(3*var(MITF)/2-2))*3,var(p53)) - floor(avg(var(Akt),var(TBX2))) + 2*min(var(FRA1),var(JUNB))	Bennett, D. C. PC&MR (2008)	High levels of MITF induces p21 mediated arrest, as can p53 activation. P21 activation can be resisted by directe phosphorylation by Akt or by Tbx2 activation.
p27	3-avg(3*var(cMyc),var(Akt),var(p90RSK),min(3*var(MITF)/2,2))+ max(0,var(Smad2_3)-var(Akt))	Maddika et al., Drug Resistance, (2007)	p27 activated unless inhibited by cMyc, Akt, p90RSK or MITF. Of these cMyc is most important as loss of Myc alone can lead to p27 mediated arrest. P27 is also activated by TGF-B signalling, although this is specifically resisted by Akt phosphorylation. MITF inhibition maxes out at level 2.
p27	3-avg(3*var(cMyc),var(Akt),var(p90RSK),min(3*var(MITF)/2,2))+ max(0,var(Smad2_3)-var(Akt))	Manning and Cantley, Cell, (2007)	p27 activated unless inhibited by cMyc, Akt, p90RSK or MITF. Of these cMyc is most important as loss of Myc alone can lead to p27 mediated arrest. P27 is also activated by TGF-B signalling, although this is specifically resisted by Akt phosphorylation. MITF inhibition maxes out at level 2.
p27	3-avg(3*var(cMyc),var(Akt),var(p90RSK),min(3*var(MITF)/2,2))+ max(0,var(Smad2_3)-var(Akt))	Lasfar et al. Carcinogenesis (2010)	p27 activated unless inhibited by cMyc, Akt, p90RSK or MITF. Of these cMyc is most important as loss of Myc alone can lead to p27 mediated arrest. P27 is also activated by TGF-B signalling, although this is specifically resisted by Akt phosphorylation. MITF inhibition maxes out at level 2.
p27	3-avg(3*var(cMyc),var(Akt),var(p90RSK),min(3*var(MITF)/2,2))+ max(0,var(Smad2_3)-var(Akt))	Carreira et al. Genes & Development (2006)	p27 activated unless inhibited by cMyc, Akt, p90RSK or MITF. Of these cMyc is most important as loss of Myc alone can lead to p27 mediated arrest. P27 is also activated by TGF-B signalling, although this is specifically resisted by Akt phosphorylation. MITF inhibition maxes out at level 2.
p38	generic		
p53	generic		
p90RSK	3*var(Erk)	Shaul and Seger, BBA, (2007)	Factor of 3 undoes automated recaling
PAR	var(PARP-1)-1	David, K. K., et al., Front Biosci (2009)	PARP-1 synthesises PAR, which is usually degraded by PARG
PARP-1	1+max(0,3*var(Erk)-4)	Hong, A. et al. Cancer Discov (2017)	High levels of ERK activity induce parthanatos
PI3K	max(2*var(HRAS),max(2*var(NRAS),var(GF-RTK)*var(FAK)))+max(0,1-var(PTEN))	Rodriguez-Viciana, P. et al., Nature, (1994)	PI3K can be activated by growth signalling (dependent on cell cell contact through FAK) directly or through RAS. Constitutive activation resisted by PTEN.
PI3K	max(2*var(HRAS),max(2*var(NRAS),var(GF-RTK)*var(FAK)))+max(0,1-var(PTEN))	Manning and Cantley, Cell, (2007)	PI3K can be activated by growth signalling (dependent on cell cell contact through FAK) directly or through RAS. Constitutive activation resisted by PTEN.
PI3K	max(2*var(HRAS),max(2*var(NRAS),var(GF-RTK)*var(FAK)))+max(0,1-var(PTEN))	Paoli, P., et al. BBA - Mol Cell Res (2013)	PI3K can be activated by growth signalling (dependent on cell cell contact through FAK) directly or through RAS. Constitutive activation resisted by PTEN.
PI3K_LC	var(Axl)+var(CSF1R)+var(Tyro3)-1	Stanley et al., CSH Pers Bio (2014)	PI3K acts downstream of RTKs
PI3K_LC	var(Axl)+var(CSF1R)+var(Tyro3)-1	Lemke et al. Nat Rev Immunology (2008)	PI3K acts downstream of RTKs
PKA	var(cAMP)-max(var(Smad2_3)-1,0)	Kaur, A., et al. BJC (2016)	PKA induced by cAMP signalling, inhibited by high levels of TGF signalling.
PKA	var(cAMP)-max(var(Smad2_3)-1,0)	Pierrat, M.-J., et al. JBC (2012)	PKA induced by cAMP signalling, inhibited by high levels of TGF signalling.
PKC	max(min(1,var(GF-RTK)),var(ROR2))	Easty, D. J., et al. PC&MR (2011)	PKC stimulated by RTK signalling, including Ror2
PKC	max(min(1,var(GF-RTK)),var(ROR2))	Dissanayake, S. K., et al. JBC (2007).	PKC stimulated by RTK signalling, including Ror2
pRB	4 -(var(CycD_CDK4))	Maddika et al., Drug Resistance, (2007)	pRB inhibited by phosphorylation by CycD-Cdk4 complex.
Proliferation	generic		
Proliferation_LC	var(TCF_LC)*var(ERK_LC)*var(mTOR_LC)/2		
Pros1	1		
PTEN	1+var(p53)-floor(var(cJUN)/2)	Hettinger et al., Cell death & Differentiation (2007)	PTEN activated by p53, constitutive expression inhibited by cJun.
PTEN	1+var(p53)-floor(var(cJUN)/2)	Stambolic et al., Mol Cell, (2001)	PTEN activated by p53, constitutive expression inhibited by cJun.
Pu.1	generic		
PUMA	var(p53)-3*var(Erk)/2	Villunger et al., Science, (2003)	Transciptional activation by p53 balanced by regulation by Erk through Foxo3
PUMA	var(p53)-3*var(Erk)/2	Cook et al. FEBS J (2017)	Transciptional activation by p53 balanced by regulation by Erk through Foxo3
ReplicationStress	1		
Residency_LC	floor(avg(var(AdhesionMolecules),var(E-cadherin),var(Id2_LC))) + 1 - ceil(var(Irf4)/2)		
ROR2	generic		
RTK	max(1,var(cJUN)-2)	Sensi, M. et al. JID (2011)	Invasive state (MITF low) associated with increased expression of various RTKs. Otherwise, expressed at normal levels.
RTK	max(1,var(cJUN)-2)	Riesenberg, S., et al. Nat Comms (2015)	Invasive state (MITF low) associated with increased expression of various RTKs. Otherwise, expressed at normal levels.
Runx3	generic		
S6K	generic		
Smad2_3	var(TBR)*ceil(3*var(TGF-B)/4)	Javelaud, D., et al. PC&MR (2008)	Smad2/3 responds to TGF-B signalling through TBR receptor.
Smad2_3_LC	generic		
Sp1	1		
Survival_LC	min(var(ERK_LC),var(mTOR_LC))		
TBR	3-ceil(var(MITF))	Javelaud, D., et al. PC&MR (2011)	TBR expression lower in MITF high state.
TBR	3-ceil(var(MITF))	Sun et al. Nature (2014)	TBR expression lower in MITF high state.
TBR	3-ceil(var(MITF))	Hoek, K. S. et al. Pigm Cell Res (2006)	TBR expression lower in MITF high state.
TBR_LC	3*var(TGF-B)/2	Hieronymous et al., Sem Cell Dev Bio (2015)	Scaled for 3 level TGF-B
TBX2	ceil(3*var(MITF)/8)	Bennett, D. C. PC&MR (2008)	MITF induces expression of TBX2, MITF varies between 0 and 3 and so adjustment is required.
TCF	generic		
TCF_LC	generic		
TGF-B	max(1, 2*var(Erk)-1)	McNeal, A. S., et al. Cancer Discovery (2015)	MAPK activation leads to TGF-B expression (could be mediated by EGR1)
TNF-a	max(var(IL-1R)-1, var(Ets1)*var(cJUN)*var(Sp1)/2)	Griffiths et al. Cytokine (2005)	Production of TNF-a by KCs is induced by high elvels of IL-1B
TNF-a	max(var(IL-1R)-1, var(Ets1)*var(cJUN)*var(Sp1)/2)	Falvo, J. V., et al. Curr dir autoimm (2010)	Expression of TNF contorlled by Sp1, cJUN/ATF-2, Ets1
TNFR	generic		
TNFR_LC	generic		
TSC	2 - avg(var(Akt),3*var(Erk))	Ma et al., Cell, (2005)	Consitutive TSC activity is inhibited by Akt and Erk.
TSC	2 - avg(var(Akt),3*var(Erk))	Inoki et al., Nat Cell Biol, (2002)	Consitutive TSC activity is inhibited by Akt and Erk.
Tyro3	max(var(Pros1),var(Gas6))*max(0,1-var(Axl))	Hieronymous et al., Sem Cell Dev Bio (2015)	Tyro3 downregulated by Axl, Pros1 and Gas6 are ligands
Tyro3	max(var(Pros1),var(Gas6))*max(0,1-var(Axl))	Bauer, T., et al. J Exp Med (2012)	Tyro3 downregulated by Axl, Pros1 and Gas6 are ligands
WNT	1		
Wnt5a	1 - floor(3*var(MITF)/4)	O'Connell, M. P. & Weeraratna, A. T. PC&MR (2009)	Wnt5a singalling associated with low MITF state, MITF varies between 0 and 3 and so adjustment is required.

Supplementary Table S8. Target functions used in the melanoma-LC model. The target function for each node is specified, along with a brief description of the mechanism and a reference where required. A number in the Target Function column represents nodes that are set to a constant value.

Supplementary Table S9. Experiments from the literature used to validate the melanoma-LC model

Line Number	Paper	Cell Line	Experiment	Constraints	Expected Results	Model Result	Notes
3	Wellbrock, C., et al. Cancer Research (2004)	melan-a	Control (TPA + cAMP in media)	cAMP High, PKC High	Proliferation High	Proliferation High	
4			Control (cAMP, no TPA in media)	cAMP High	Proliferation OFF	Proliferation OFF	
5			BRAFV600E (TPA + cAMP in media)	BRAFV600E, cAMP High, PKC High, MITF High	Proliferation Very High	Proliferation Very High	Wellbrock, C., & Marais, R. JCB (2005) state that these cells become less differentiated.
6			BRAFV600E (cAMP, no TPA in media)	BRAFV600E, cAMP High, MITF High	Proliferation Very High	Proliferation Very High	
7	Wellbrock, C., & Marais, R. JCB (2005)	melan-a	Control	BRAFV600E, MITF High	Proliferation Very High	Proliferation Very High	
8			Control + MITF overexpression	BRAFV600E, MITF Very High	Proliferation High	Proliferation High	
9	Whitwam, T., et al. Oncogene (2007)	D6-MEL	Control (soft agar growth)	CellCellContact OFF	Proliferation OFF	Proliferation OFF	
10			NRASG12V (soft agar growth)	CellCellContact OFF, NRAS Mid	Proliferation Very High	Proliferation Very High	As Wellbrock, C., & Marais, R. (2005), assume MITF downregulated when MAPK activated.
11	Yang, J. et al. J Clin Invest (2010)	dox-inducible HRASG12V CDKN2A-/- mouse melanoma model	Control (TPA in media)	HRAS Mid, CDKN2AB OFF, PKC High	Apoptosis OFF	Apoptosis OFF	
12			IKK-/- mice	HRAS Mid, CDKN2AB OFF, PKC High, IKK OFF	Apoptosis Mid	Apoptosis Mid	
13			IKK-/- mice, p53 siRNA	HRAS Mid, CDKN2AB OFF, PKC High, IKK OFF, p53 OFF	Apoptosis OFF	Apoptosis OFF	
14	Swope, V. B., et al. Exp Cell Res (1995)	Normal human melanocytes	Control (normal media with bFGF and FBS)	MITF High, GF Low, ReplicationStress OFF	Proliferation OFF	Proliferation OFF	
15			Normal media + ET-1 growth factor	MITF High, GF Mid, ReplicationStress OFF	Proliferation OFF	Proliferation OFF	
16			Normal media + α-MSH	MITF High, GF Low, ReplicationStress OFF, a-MSH High	Proliferation OFF	Proliferation OFF	
17			Normal media + ET-1 + α-MSH	MITF High, GF Mid, ReplicationStress OFF, a-MSH High	Proliferation Mid	Proliferation Mid	
18	McNeal, A. S. et al. Cancer Discov (2015)	Normal human melanocytes	Naevus cell	BRAFV600E ON, MITF Very High, GF Mid, cAMP High, ReplicationStress ON	Proliferation OFF	Proliferation OFF	Presumably media contains cholera toxin or equivalent, but details of media not provided.
19			Normal melanocyte	MITF Very High, GF Mid, cAMP High	Proliferation Mid	Proliferation Mid	
20			Normal melanocyte + p15 expression	MITF Very High, GF Mid, cAMP High, p15 High	Proliferation OFF	Proliferation OFF	
21			Normal melanocyte + p16 expression	MITF Very High, GF Mid, cAMP High, p16 High	Proliferation Low	**Proliferation OFF**	
22			doxycyclin-induced BRAFV600E Cell + p15 shRNA	BRAFV600E ON, MITF Very High, GF Mid, cAMP High, p15 OFF	Proliferation Low	Proliferation Low	
23			doxycyclin-induced BRAFV600E Cell + p16 shRNA	BRAFV600E ON, MITF Very High, GF Mid, cAMP High, p16 OFF	Proliferation OFF	**Proliferation Low**	
24			Normal melanocyte + TGF-β	MITF Very High, GF Mid, cAMP High, TGF-B High	Proliferation OFF	Proliferation OFF	
25			Naevus cell (growth in tissue) + CDKR24C mutation	BRAFV600E ON, MITF Very High, GF Mid, cAMP High, ReplicationStress ON, CDK4 High	Proliferation Low	Proliferation Low	
26			Naevus cell (growth in tissue) + CDKR24C mutation + TP53R284W (dominant negative)	BRAFV600E ON, MITF Very High, GF Mid, cAMP High, ReplicationStress ON, CDK4 High, p53 OFF	Proliferation Mid	Proliferation Mid	
27	Byron, S. A., et al Mol Cancer (2012)	UACC903	Control		Proliferation Very High, Apoptosis OFF	Proliferation Very High, Apoptosis OFF	
28			MEKi (E6201)	MEKi ON	Proliferation OFF, Apoptosis Low	Proliferation OFF, Apoptosis Low	
29			PI3Ki (LY294002)	PI3K OFF	Proliferation OFF, Apoptosis Low	***Proliferation Low***, Apoptosis Low	
30			MEKi (E6201) + PI3Ki (LY294002)	MEKi ON + PI3K OFF	Proliferation OFF, Apoptosis Very High	Proliferation OFF, Apoptosis Very High	
31		WM35	Control		Proliferation Very High, Apoptosis OFF	Proliferation Very High, Apoptosis OFF	
32			MEKi (E6201)	MEKi ON	Proliferation OFF, Apoptosis High	Proliferation OFF, Apoptosis High	
33			PI3Ki (LY294002)	PI3K OFF	Proliferation OFF, Apoptosis Low	***Proliferation Low***, Apoptosis Low	
34			MEKi (E6201) + PI3Ki (LY294002)	MEKi ON + PI3K OFF	Proliferation OFF, Apoptosis Very High	Proliferation OFF, Apoptosis Very High	
35	Cheung, M., et al. Cancer Research (2008)	WM35	Control (anchorage-independent growth)	CellCellContact OFF	Apoptosis Low	Apoptosis Low	
36			BRAF siRNA (anchorage-independent growth)	CellCellContact OFF, BRAF OFF	Apoptosis Very High	Apoptosis Very High	
37			Myristoylated-Akt3 expression	CellCellContact OFF, Akt High	Apoptosis OFF	Apoptosis OFF	
38		UACC903	Control (anchorage-independent growth, no serum)	CellCellContact OFF, GF OFF	Proliferation Very High, Apoptosis OFF	Proliferation Very High, Apoptosis OFF	
39			BRAF siRNA (anchorage-independent growth, no serum)	CellCellContact OFF, GF OFF, BRAF OFF	Proliferation OFF, Apoptosis Very High	Proliferation OFF, Apoptosis Very High	
40			Akt3 siRNA (anchorage-independent growth, no serum)	CellCellContact OFF, GF OFF, Akt OFF	Proliferation OFF, Apoptosis Low	***Proliferation Low***, Apoptosis Low	
41			BRAF + Akt3 siRNA (anchorage-independent growth, no serum)	CellCellContact OFF, GF OFF, Akt OFF, BRAF OFF	Proliferation OFF, Apoptosis Very High	Proliferation OFF, Apoptosis Very High	
42	Zhuang, D. et al. Oncogene (2008)	SK-MEL-19	Control		Proliferation Very High	Proliferation Very High	Note that cellosaurus describes p53 mutation in both cell lines but Zhuang et al claim pathway is intact, p53 shRNA experiments they perform show this is not relevant to result.
43			c-Myc shRNA	cMyc OFF	Proliferation OFF	**Proliferation Low/Mid**	
44			p53 shRNA	p53 OFF	Proliferation Very High	Proliferation Very High	
45			c-Myc + p53 shRNA	c-Myc + p53 shRNA	Proliferation OFF	**Proliferation Low/Mid**	
46		SK-MEL-2	Control		Proliferation Very High	Proliferation Very High	
47			c-Myc shRNA	cMyc OFF	Proliferation OFF	**Proliferation Low/Mid**	
48			p53 shRNA	p53 OFF	Proliferation Very High	Proliferation Very High	
49			c-Myc + p53 shRNA	c-Myc + p53 shRNA	Proliferation OFF	**Proliferation Low/Mid**	
50	Solit, D. B., et al. Nature (2006)	Malme-3M	MEKi (CI-1040)	MEKi ON	Proliferation OFF, Apoptosis High	Proliferation OFF, Apoptosis High	Same control as Zhuang et al. (2008) SK-MEL-19.
51		SK-MEL-103	MEKi (CI-1040)	MEKi ON	Proliferation Low, Apoptosis Low	**Proliferation OFF**, Apoptosis Low	Same control as Zhuang et al. (2008) SK-MEL-2.
52	Shellman, Y. G., et al. JID (2000)	WM35	TGF-β	TGF-B High	Proliferation High	Proliferation High	Same control as Byron, S. A., et al Mol Cancer (2012) WM35.
53			NRASG12D expression	NRAS Mid	Proliferation Very High	Proliferation Very High	
54			TGF-β + NRASG12D expression	TGF-B High, NRAS Mid	Proliferation Very High	Proliferation Very High	
55	Donovan, J. C. H., et al. JBC (2002)	WM35	TGF-β + p27 anti-sense oligonucleotide	TGF-B High + p27 OFF	Proliferation Very High	Proliferation Very High	Same control as Shellman, Y. G., et al. JID (2000) WM35.
56	Liang, J.,et al. Nature Medicine (2002)	WM239	Control		Proliferation Very High	Proliferation Very High	
57			TGF-β	TGF-B High	Proliferation Very High	Proliferation Very High	
58			TGF-β + PTEN expression	TGF-B High, PTEN WT	Proliferation High	Proliferation High	
59	Verhaegen, M., et al. Cancer Research (2006)	SK-MEL-103	Control		Apoptosis OFF	Apoptosis OFF	
60			MEKi (U0126)	MEKi ON	Apoptosis Low	Apoptosis Low	
61			Bcl-2 shRNA	Bcl-2 OFF	Apoptosis OFF	Apoptosis OFF	
62			Mcl-1 shRNA	Mcl-1 OFF	Apoptosis Low	Apoptosis Low	
63			Bcl-2 shRNA + MEKi (U0126)	MEKi ON, Bcl-2 OFF	Apoptosis Low	Apoptosis Low	
64			Mcl-1 shRNA + MEKi (U0126)	MEKi ON, Mcl-1 OFF	Apoptosis High	Apoptosis High	
65			TW-37 (BH3 mimetic) + MEKi (U0126)	MEKi ON, Mcl-1 OFF, Bcl-2 OFF	Apoptosis High	Apoptosis High	
66			TW-37 (BH3 mimetic) + MEKi (U0126) + p53 shRNA	MEKi ON, Mcl-1 OFF, Bcl-2 OFF, p53 OFF	Apoptosis Low	**Apoptosis OFF**	
67			TW-37 (BH3 mimetic) + MEKi (U0126) + Caspase inhibitor (zVAD)	MEKi ON, Mcl-1 OFF, Bcl-2 OFF, Caspase-3 OFF	Apoptosis Mid	Apoptosis Mid	
68		Normal human melanocytes	TW-37 (BH3 mimetic) + MEKi (U0126)	ReplicationStress OFF, MITF Very High, MEKi ON, Mcl-1 OFF, Bcl-2 OFF	Apoptosis OFF	Apoptosis OFF	

69	Posch, C. et al. PNAS (2013)	d04	Control		Proliferation Very High, Apoptosis OFF	Proliferation Very High, Apoptosis OFF	
70			MEKi (JTP-74057)	MEKi ON	Proliferation OFF, Apoptosis Low	Proliferation OFF, Apoptosis Low	
71			PI3K/mTORC1/2i (GSK2126458)	PI3K OFF, mTORC1 OFF, mTORC2 OFF	Proliferation OFF, Apoptosis Low	**Proliferation OFF/Low**, Apoptosis Low	
72			PI3K/mTORC1/2i (GSK2126458) + MEKi (JTP-74057)	PI3K OFF, mTORC1 OFF, mTORC2 OFF, MEKi ON	Proliferation OFF, Apoptosis Very High	Proliferation OFF, Apoptosis Very High	
73	Straussman, R., et al. Nature (2012)	SK-MEL-5	Control		Proliferation Very High, Apoptosis OFF	Proliferation Very High, Apoptosis OFF	
74			BRAFi PLX-4720	BRAFi ON	Proliferation OFF, Apoptosis High	Proliferation OFF, Apoptosis High	
75			BRAFi PLX-4720 + HGF	BRAFi ON, GF Very High	Proliferation Very High, Apoptosis OFF	Proliferation Very High, Apoptosis OFF	
76	Konieczkowski, D. J., et al. Cancer Discovery (2014)	WM266-4	Control		Proliferation Very High, Apoptosis OFF	Proliferation Very High, Apoptosis OFF	
77			ERKi (VTX11E)	ERKi ON	Proliferation OFF, Apoptosis Low	Proliferation OFF, Apoptosis Low	
78			ERKi (VTX11E) + TNF-α	ERKi ON, TNF-a High, MITF Low	Proliferation Mid, Apoptosis OFF	Proliferation Mid, Apoptosis OFF	TNF-α induces MITF low state.
79		A375	Control		Proliferation Very High, Apoptosis OFF	Proliferation Very High, Apoptosis OFF	Using MITF level determined by Muller et al 2014, not Wouters et al 2020.
80			BRAFi (PLX4720)	BRAFi ON	Proliferation OFF, Apoptosis High	Proliferation OFF, Apoptosis High	
81			MEKi (AZD2644)	MEKi ON	Proliferation OFF, Apoptosis High	Proliferation OFF, Apoptosis High	
82			ERKi (VTX11E)	ERKi ON	Proliferation OFF, Apoptosis High	Proliferation OFF, Apoptosis High	
83			AXL overexpression	RTK Very High	Proliferation Very High, Apoptosis OFF	Proliferation Very High, Apoptosis OFF	
84			BRAFi (PLX4720) + AXL overexpression	BRAFi ON, RTK Very High	Proliferation Very High, Apoptosis OFF	Proliferation Very High, Apoptosis OFF	
85			MEKi (AZD2644) + AXL overexpression	MEKi ON, RTK Very High	Proliferation Very High, Apoptosis OFF	**Proliferation OFF, Apoptosis Low**	
86			ERKi (VTX11E) + AXL overexpression	ERKi ON, RTK Very High	Proliferation OFF, Apoptosis Low	Proliferation OFF, Apoptosis Low	
87	Müller, J. et al. Nat Commun (2014)	WM266-4	BRAFi (PLX4720)	BRAFi ON	Proliferation OFF, Apoptosis Low	Proliferation OFF, Apoptosis Low	Same control as Konieczkowski, D. J., et al. Cancer Discovery (2014) WM266-4
88			BRAFi resistant clone (MITF low) + BRAFi (PLX4720)	BRAFi ON, MITF Low	Proliferation High, Apoptosis OFF	Proliferation High, Apoptosis OFF	
89			MEKi (Trametinib)	MEKi ON	Proliferation OFF, Apoptosis Low	Proliferation OFF, Apoptosis Low	
90			BRAFi resistant clone (MITF low) + MEKi (Trametinib)	MEKi ON, MITF Low	Proliferation Mid, Apoptosis OFF	Proliferation Mid, Apoptosis OFF	
91			ERKi (SCH772984)	ERKi ON	Proliferation OFF, Apoptosis Low	Proliferation OFF, Apoptosis Low	
92			BRAFi resistant clone (MITF low) + ERKi (SCH772984)	ERKi ON, MITF Low	Proliferation Mid, Apoptosis OFF	Proliferation Mid, Apoptosis OFF	
93	Shi, H., et al. Nat Commun (2012)	M395	Control		Apoptosis OFF	Apoptosis OFF	
94			BRAFi (Vemurafenib)	BRAFi ON	Apoptosis High	**Apoptosis OFF**	
95			BRAFV600E overexpression	BRAFamplification Low	Apoptosis OFF	Apoptosis OFF	
96			BRAFV600E overexpression + BRAFi (Vemurafenib)	BRAFamplification Low, BRAFi ON	Apoptosis OFF	Apoptosis OFF	
97			BRAFV600E overexpression + high BRAFi (Vemurafenib)	BRAFamplification Low, BRAFi High	Apoptosis High	**Apoptosis OFF**	
98			BRAFi resistant clone (BRAFV600E overexpression) + MEKi (AZD6244)	BRAFamplification Low, MEKi ON	Apoptosis OFF	Apoptosis OFF	
99		M249	BRAFi resistant clone (NRASQ61K mutation)	NRAS Mid	Apoptosis OFF	Apoptosis OFF	
100			BRAFi resistant clone (NRASQ61K mutation) + BRAFi (Vemurafenib)	NRAS Mid, BRAFi ON	Apoptosis OFF	Apoptosis OFF	
101			BRAFi resistant clone (NRASQ61K mutation) + MEKi (AZD6244)	NRAS Mid, MEKi ON	Apoptosis High	***Apoptosis Low***	
102	Wagle, N. et al., Cancer Discov (2014)	A375	MEK2Q60P expression + BRAFi (dabrafenib)	MEKGoF ON, BRAFi ON	Proliferation Very High, Apoptosis OFF	Proliferation Very High, Apoptosis OFF	Using MITF level determined by Muller et al 2014, not Wouters et al 2020; Same control as Konieczkowski, D. J., et al. Cancer Discovery (2014) A375
103			MEK2Q60P expression + BRAFi (dabrafenib) + MEKi (trametinib)	MEKGoF ON, BRAFi ON, MEKi ON	Proliferation Very High, Apoptosis OFF	Proliferation Very High, Apoptosis OFF	
104			MEK2Q60P expression + ERKi (VTX11E)	MEKGoF ON, ERKi ON	Proliferation OFF, Apoptosis High	**Proliferation Very High, Apoptosis OFF**	
105	Allen, E. M. V., et al., Cancer Discov (2014)	WM266-4	MITF overexpression	MITF Very High	Apoptosis OFF	Apoptosis OFF	Same control as Konieczkowski, D. J., et al. Cancer Discovery (2014) WM266-4.
106			MITF overexpression + BRAFi (dabrafenib)	MITF Very High, BRAFi ON	Apoptosis OFF	Apoptosis OFF	
107			MITF overexpression + MEKi (trametinib)	MITF Very High, MEKi ON	Apoptosis OFF	Apoptosis OFF	
108			MITF overexpression + ERKi (VTX11E)	MITF Very High, ERKi ON	Apoptosis OFF	Apoptosis OFF	
109		UACC-62	Control		Proliferation Very High, Apoptosis OFF	Proliferation Very High, Apoptosis OFF	
110			BRAFi (PLX4720)	BRAFi ON	Proliferation OFF, Apoptosis Low	Proliferation OFF, Apoptosis Low	
111			BRAFi (PLX4720) + MITF overexpression	BRAFi (PLX4720) + MITF overexpression	Proliferation Low, Apoptosis OFF	**Proliferation OFF**, Apoptosis OFF	
112	Thakur, M. D. et al. Nature (2013)	HMEX1906	Control		Proliferation Very High, Apoptosis OFF	Proliferation Very High, Apoptosis OFF	
113			BRAFi-resistant clone (BRAF amplification)	BRAFamplification Low	Proliferation Mid, Apoptosis OFF	***Proliferation Low***, Apoptosis OFF	
114			BRAFi-resistant clone (BRAF amplification) + BRAFi	BRAFamplification Low, BRAFi ON	Proliferation Very High, Apoptosis OFF	Proliferation Very High, Apoptosis OFF	
115			BRAFi-resistant clone (BRAF amplification) + high BRAFi	BRAFamplification Low, BRAFi High	Proliferation OFF, Apoptosis Low	Proliferation OFF, Apoptosis Low	
116	Moriceau, G. et al. Cancer Cell (2015)	M249	BRAFi + MEKi	BRAFi ON, MEKi ON	Proliferation OFF, Apoptosis Low	Proliferation OFF, Apoptosis Low	
117			BRAFamplification + BRAFi + MEKi	BRAFamplification Low, BRAFi ON, MEKi ON	Proliferation OFF, Apoptosis Low	Proliferation OFF, Apoptosis Low	
118			MEK1F129L + BRAFi + MEKi	MEKGoF ON, BRAFi ON, MEKi ON	Proliferation OFF, Apoptosis Low	**Proliferation Very High, Apoptosis OFF**	
119			BRAFamplification + MEK1F129L + BRAFi + MEKi	BRAFamplification Low, MEKGoF ON, BRAFi ON, MEKi ON	Proliferation Very High, Apoptosis OFF	Proliferation Very High, Apoptosis OFF	
120			BRAF ultra-amplification + BRAFi + MEKi	BRAFamplification High, BRAFi ON, MEKi ON	Proliferation Very High, Apoptosis OFF	Proliferation Very High, Apoptosis OFF	
121			BRAFi + MEKi resistant clone (BRAFamplification + MEK1F129L) without drugs	BRAFamplification Low, MEKGoF ON	Proliferation Mid, Apoptosis Mid	Proliferation Mid, Apoptosis Mid	
122			BRAFi + MEKi resistant clone (BRAF ultra-amplification) without drugs	BRAFamplification High	Proliferation Mid, Apoptosis Mid	Proliferation Mid, Apoptosis Mid	
123			BRAFi + MEKi resistant clone (BRAFamplification + MEK1F129L) + ERKi (SCH772984)	BRAFamplification Low, MEKGoF ON, ERKi ON	Proliferation Very High, Apoptosis OFF	**Proliferation Mid**, Apoptosis OFF	
124			BRAFi + MEKi resistant clone (BRAF ultra-amplification) + ERKi (SCH772984)	BRAFamplification High, ERKi ON	Proliferation Very High, Apoptosis OFF	**Proliferation Mid**, Apoptosis OFF	
125			BRAFi resistant clone (NRASQ61K) + BRAFi	NRAS Mid, BRAFi ON	Proliferation Very High, Apoptosis OFF	Proliferation Very High, Apoptosis OFF	
126			BRAFi resistant clone (BRAF NRASQ61K)	NRAS Mid	Proliferation Very High, Apoptosis OFF	Proliferation Very High, Apoptosis OFF	
127		M395	BRAFi resistant clone (BRAF amplification) + BRAFi	BRAFamplifcation Low, BRAFi ON	Proliferation Very High, Apoptosis OFF	**Proliferation Mid**, Apoptosis OFF	
128			BRAFi resistant clone (BRAF amplification)	BRAFamplifcation Low	Proliferation Very High, Apoptosis OFF	**Proliferation OFF**, Apoptosis OFF	
129		M229	BRAFi resistant clone (PDGFRβ upregulation) + BRAFi	RTK High, BRAFi ON	Proliferation Very High, Apoptosis OFF	Proliferation Very High, Apoptosis OFF	
130			BRAFi resistant clone (PDGFRβ upregulation)	RTK High	Proliferation Very High, Apoptosis OFF	Proliferation Very High, Apoptosis OFF	
131	Johannessen, C. M. et al. Nature	WM266-4	cAMP induction (Forskolin)	cAMP High, MITF Very High	Apoptosis OFF	Apoptosis OFF	Forskolin induces MITF expression.
132			cAMP induction (Forskolin) + BRAFi (PLX4720)	cAMP High, MITF Very High, BRAFi ON	Apoptosis OFF	Apoptosis OFF	
133			cAMP induction (Forskolin) + BRAFi (PLX4720) + MITF shRNA	cAMP High, MITF OFF, BRAFi ON	Apoptosis Mid	Apoptosis Mid	
134			MITF shRNA	MITF OFF	Apoptosis OFF	Apoptosis OFF	
135			BRAFi (PLX4720) + MITF shRNA	MITF OFF, BRAFi ON	Apoptosis Mid	Apoptosis Mid	

136	(2013)		BRAFi (PLX4720)	BRAFi ON	Apoptosis Low	Apoptosis Low	
137			cAMP induction (Forskolin) + BRAFi (PLX4720) + CREB dominant negative (CREBR301L)	cAMP High, BRAFi ON, CREB OFF	Apoptosis Low	Apoptosis Low	CREB required for induction of MITF (Goding, C. R. & Arnheiter, H. Gene Dev (2019)).
138	Kel et al. J Immunology (2010)	Normal Mouse Langerhans Cells	TGF-βR1-/-	ReplicationStress OFF, TBR_LC OFF	Survival_LC Mid, Residency_LC OFF, Proliferation_LC Mid	Survival_LC Mid, Residency_LC Low, Proliferation_LC Mid	
139	Sparber, F.,et al. Blood (2014)	Normal Mouse Langerhans Cells	p14-/-	ReplicationStress OFF, LAMTOR-p14 OFF	Survival_LC OFF, Residency_LC Mid, Proliferation_LC OFF	Survival_LC OFF, Residency_LC Mid, Proliferation_LC OFF	
140	Cumberbatch, M., Fielding, I., & Kimber, I. (1994)	Normal Mouse Langerhans Cells	TNF-α injection	ReplicationStress OFF, TNF-a High	Residency_LC OFF	Residency_LC OFF	
141	Kellersch, B., & Brocker, T. Blood (2013)	Normal Mouse Langerhans Cells	Raptor-/- (essential component of mTORC1)	ReplicationStress OFF, mTOR_LC OFF	Survival_LC OFF	Survival_LC OFF	
142	Borkowski, T. A., et al. J Exp Med (1996)	Normal Mouse Langerhans Cells	TGF-β1-/-	ReplicationStress OFF, TGF-B OFF	Residency_LC OFF	Residency_LC OFF	
143	Chopin, M., et al. J Exp Med (2013)	Normal Mouse Langerhans Cells	Pu.1-/-	ReplicationStress OFF, Pu.1 OFF	Residency_LC OFF	Residency_LC OFF	
144			Id2-/-	ReplicationStress OFF, Id2_LC OFF	Residency_LC OFF	Residency_LC OFF	
145	Bauer, T., et al. J Exp Med (2012)	Normal Mouse Langerhans Cells	Tyro3-/-, Axl-/-	ReplicationStress OFF, Tyro3 OFF, Axl OFF	Survival_LC OFF	Survival_LC OFF	Also Mertk-/-
146			Axl-/-	ReplicationStress OFF, Axl OFF	Survival_LC Mid	Survival_LC Mid	
147	Wang, Y., et al. Nat immunology (2012)	Normal Mouse Langerhans Cells	IL-34-/-	ReplicationStress OFF, IL-34 OFF	Survival_LC OFF	Survival_LC OFF	
148	Becker, M. R., et al. JID (2011)	Normal Mouse Langerhans Cells	DKK overexpression	ReplicationStress OFF, DKK High	Proliferation_LC OFF	Proliferation_LC OFF	
149	Cumberbatch, M.,et al. Immunology (2001)	Normal Mouse Langerhans Cells	IL-18 injection	ReplicationStress OFF, IL-18 High	Residency_LC OFF	Residency_LC OFF	
150			IL-18 injection + anti-TNF-α Ab	ReplicationStress OFF, IL-18 High, TNF-a OFF	Residency_LC Mid	Residency_LC Mid	
151			IL-18 injection + anti-IL-1R Ab	ReplicationStress OFF, Il-18 High, IL-1R OFF	Residency_LC Mid	Residency_LC Mid	
152	Cumberbatch, M., et al. Immunology (1997)	Normal Mouse Langerhans Cells	IL-1β injection	ReplicationStress OFF, IL-1B High	Residency_LC OFF	Residency_LC OFF	
153			IL-1β injection +anti-TNF-α Ab	ReplicationStress OFF, IL-1B High, TNF-a OFF	Residency_LC Mid	Residency_LC Mid	
154			TNF-α injection + anti-IL-1β Ab	ReplicationStress OFF, TNF-a High, IL-1B OFF	Residency_LC Mid	Residency_LC Mid	

Supplementary Table S9. Experiments from the literature used to validate the melanoma-LC model. For each experiment, the table shows the publication, the cell line used, the constraints applied to the model and the expected outcome, based on the experimental result. The sixth column shows the result of the model simulations, with deviations from the expected results highlighted in bold; bold results in italics are cases where the trend is correct but exact numerical value is not matched.

Supplementary Table S10. Constraints used for melanoma-LC model screening

Background			node	activity
BRAF	MITF	PTEN		
WT	Very High	WT	ReplicationStress	0
			MITF	3
			a-MSH	1
			GF	2
BRAFV600E	High	-/-	BRAFV600E	1
			PTEN	0
			CDKN2AB	0
			MITF	2
			a-MSH	1
			GF	2
BRAFV600E	High	WT	BRAFV600E	1
			CDKN2AB	0
			MITF	2
			a-MSH	1
			GF	2
BRAFV600E	Low	WT	BRAFV600E	1
			CDKN2AB	0
			MITF	1
			a-MSH	1
			GF	2
BRAFV600E	Low	-/-	BRAFV600E	1
			CDKN2AB	0
			MITF	1
			PTEN	0
			a-MSH	1
			GF	2

Supplementary Table S11. Targeted therapies used for melanoma-LC model screening

Drug	Node	Activity	Reference
Abemaciclib	CDK4	0	Spring, L. M., et al. Curr Oncol Rep (2019)
Ac-ATS010-KE	Caspase-3	0	Solania, A., et al. Acs Chem Biol (2019)
AGX51	Id2	0	Wojnarowicz, P. M. et al. Cell Reports (2019)
AGX51	Id2_LC	0	Wojnarowicz, P. M. et al. Cell Reports (2019)
Atrosimab	TNFR	0	Fischer, R., Kontermann, R. E. & Pfizenmaier, K. Frontiers Cell Dev Biology (2020)
Atrosimab	TNFR_LC	0	Fischer, R., Kontermann, R. E. & Pfizenmaier, K. Frontiers Cell Dev Biology (2020)
AZD5991	Mcl-1	0	Bolomsky, A. et al. J Hematol Oncol (2020)
BC21	TCF	0	Anastas, J. N. & Moon, R. T. Nat Rev Cancer (2013)
BC21	TCF_LC	0	Anastas, J. N. & Moon, R. T. Nat Rev Cancer (2013)
Birinapant	IAP	0	Benetatos, C. A. et al. Mol Cancer Ther (2014)
Birinapant	ML-IAP	0	Benetatos, C. A. et al. Mol Cancer Ther (2014)
BMS-34554	IKK	0	Paul, A., et al. Cells (2018)
BpV(phen)	PTEN	0	Pulido, R. Molecules (2018)
CA5	TBX2	0	Sahm, B. D. B. et al. Front Chem (2020)
Capivasertib	Akt	0	Martorana, F. et al. Front Pharmacol (2021)
Capivasertib	Akt_LC	0	Martorana, F. et al. Front Pharmacol (2021)
Copanlisib	PI3K	0	Vanhaesebroeck, B., et al. Nat Rev Drug Discov (2021)
Copanlisib	PI3K_LC	0	Vanhaesebroeck, B., et al. Nat Rev Drug Discov (2021)
Dabrafenib	BRAFi	1	Luebker, S. A. & Koepsell, S. A. Frontiers Oncol (2019)
Defactinib	FAK	0	Lv, P.-C., et al. Expert Opin Ther Pat (2017)
Eltrombopag	BaxBak	0	Spitz, A. Z., et al. Nat Commun (2021)
Everolimus	mTORC1	0	Feng, Y. et al. Frontiers Oncol (2021)
Everolimus	mTOR_LC	0	Feng, Y. et al. Frontiers Oncol (2021)
FMK	p90RSK	0	Ludwik, K. A. & Lannigan, D. A. Expert Opin Ther Pat (2016)
GW788388	TBR	0	Walton, K. L., Front Pharmacol (2017)
GW788388	TBR_LC	0	Walton, K. L., Front Pharmacol (2017)
HLM0064749	E2F	0	Rouaud, F. et al. Cell Death Dis (2018)
HLY78	AXIN	0	Wang, S. et al. Nat Chem Biol (2013)
HLY78	AXIN_LC	0	Wang, S. et al. Nat Chem Biol (2013)
Idasanutlin	Mdm	0	Tisato, V., et al. J Hematol Oncol (2017)
Infliximab	TNF-a	0	Smolen, J. S. & Emery, P. Arthritis Res Ther (2011)
JR-AB2-011	mTORC2	0	Benavides-Serrato, A. et al. Plos One (2017)
LF3	B-catenin	0	Zhang, Y. & Wang, X. J Hematol Oncol (2020)
LF3	B-catenin_LC	0	Zhang, Y. & Wang, X. J Hematol Oncol (2020)
LY2584702	S6K	0	LoRusso, P. M. J Clin Oncol (2016)
Omomyc	cMyc	0	Beaulieu, M.-E. et al. Sci Transl Med (2019)
PORCN	WNT	0	Anastas, J. N. & Moon, R. T. Nat Rev Cancer (2013)
PPL-008	CRAF	0	Blair, C. M., et al. Bmc Cancer (2019)
SIS3	Smad2_3	0	Walton, K. L., Front Pharmacol (2017)
SIS3	Smad2_3_LC	0	Walton, K. L., Front Pharmacol (2017)
Sotrastaurin	PKC	0	Parker, P. J. et al. Nat Rev Cancer (2021)
SP600125	JNK	0	Wu, Q. et al. J Enzym Inhib Med Ch (2020)
T-5224	cJUN	0	Brennan, A., et al. J Exp Clin Canc Res (2020)
TGF-β1 mAb	TGF-B	0	Walton, K. L., Front Pharmacol (2017)
Tideglusib	GSK3b	0	Saraswati, A. P., et al. Eur J Med Chem (2018)
Trametinib	MEKi	1	Luebker, S. A. & Koepsell, S. A. Frontiers Oncol (2019)
Trametinib	MEK_LC	0	Luebker, S. A. & Koepsell, S. A. Frontiers Oncol (2019)
UC2288	p21	0	Wettersten, H. I. et al. Cancer Biol Ther (2013)
Ulixertinib	ERKi	1	Sullivan, R. J. et al. Cancer Discov (2017)
Ulixertinib	ERK_LC	0	Sullivan, R. J. et al. Cancer Discov (2017)
Vantictumab	Frizzled	0	Zhang, Y. & Wang, X. J Hematol Oncol (2020)
Vantictumab	Frizzled_LC	0	Zhang, Y. & Wang, X. J Hematol Oncol (2020)
Venetoclax	Bcl-2	0	Ryan, C. E. & Davids, M. S. Cancer J (2019)

Supplementary Table S11. Targeted therapies used for melanoma-LC model screening. For each drug, the affected nodes and the level of activity used to model the drug's effect are shown, along with a reference describing the drug's mechanism of action.

Supplementary Table S12. Primer list

Oligo name	Oligo sequence (5' to 3')
TNF_F	CAGGCGGTGCCTATGTCTC
TNF_R	CGATCACCCCGAAGTTCAGTAG
IL1b_F	GAAATGCCACCTTTTGACAGTG
IL-1b_R	TGGATGCTCTCATCAGGACAG
TGFB1_F	CCACCTGCAAGACCATCGAC
TGFB1_R	CTGGCGAGCCTTAGTTTGGAC
DKK1_F	CTCATCAATTCCAACGCGATCA
DKK1_R	GCCCTCATAGAGAACTCCCG
CSF1_F	GTGTCAGAACACTGTAGCCAC
CSF1_R	TCAAAGGCAATCTGGCATGAAG
IL34_F	TTGCTGTAAACAAAGCCCCAT
IL34_R	CCGAGACAAAGGGTACACATTT
PROS1_F	CCAAAGAGCGTGCTTCACAAG
PROS1_R	TCTTCAATGCACTCTCGTTCAAG
GAS6_F	CCGCGCCTACCAAGTCTTC
GAS6_R	CGGGGTCGTTCTCGAACAC
MKI67_F	ATCATTGACCGCTCCTTTAGGT
MKI67_R	GCTCGCCTTGATGGTTCCT
B-actin_F	GGCTGTATTCCCCTCCATCG
B-actin_R	CCAGTTGGTAACAATGCCATGT

Supplementary Table S13. Genetic backgrounds of cell lines simulated

Cell Line	Species	Gene	Activity	Source
A375	human	MITF	1	Wouters, J. et al. Nat Cell Biol (2020)
		**MITF**	**2**	**Müller, J. et al. Nat Commun (2014)**
		BRAFV600E	1	Byron, S. A., et al Mol Cancer (2012)
		CDKN2AB	0	Byron, S. A., et al Mol Cancer (2012)
d04	human	NRAS	2	Cellosaurus
		CDKN2AB	0	Cellosaurus
		MITF	2	assumption
D6-MEL	mouse	CDKN2AB	0	Whitwam, T., et al. Oncogene (2007)
		ReplicationStress	0	Untransformed cell line
		MITF	3	Untransformed cell line
HMEX1906	human	BRAFV600E	1	Thakur, M. D., et al. Nature (2013)
		CDKN2AB	0	Thakur, M. D., et al. Nature (2013)
		MITF	2	Thakur, M. D., et al. Nature (2013)
M229	human	BRAFV600E	1	Su, Y. et al. PNAS (2017)
		PTEN	0	Su, Y. et al. PNAS (2017)
		Akt	2	Su, Y. et al. PNAS (2017)
		MITF	2	Su, Y. et al. PNAS (2017)
		CDKN2AB	0	assumption
M249	human	BRAV600E	1	Su, Y. et al. PNAS (2017)
		PTEN	0	Su, Y. et al. PNAS (2017)
		Akt	2	Su, Y. et al. PNAS (2017)
		MITF	2	Su, Y. et al. PNAS (2017)
		CDKN2AB	0	assumption
M395	human	BRAFV600E	1	Su, Y. et al. PNAS (2017)
		CDKN2AB	0	assumption
		MITF	1	Su, Y. et al. PNAS (2017)
Malme-3M	human	BRAFV600E	1	Cellosaurus
		MITF	2	assumption
		CDKN2AB	0	Cellosaurus
melan-a	mouse	p16	0	Sviderskaya, E. V., et al. (2002)
		ARF	0	Sviderskaya, E. V., et al. (2002)
		ReplicationStress	0	Untransformed cell line
		MITF	3	Untransformed cell line
SK-Mel-103	human	NRAS	2	Cellosaurus
		CDKN2AB	0	assumption
		MITF	2	assumption
SK-Mel-19	human	CDKN2AB	0	Cellosaurus
		BRAFV600E	1	Cellosaurus
		p53	0	Cellosaurus
		**p53**	**wild type**	**Zhuang, D. et al. Oncogene (2008)**
		MITF	2	assumption
SK-Mel-2	human	NRAS	2	Cellosaurus
		p53	0	Cellosaurus
		**p53**	**wild type**	**Zhuang, D. et al. Oncogene (2008)**
		CDKN2AB	0	assumption
		MITF	2	assumption
SK-Mel-5	human	MITF	2	Konieczkowski, D. J., et al. Cancer Discovery (2014)
		BRAFV600E	1	Cellosaurus
		CDKN2AB	0	Cellosaurus
UACC-62	human	BRAFV600E	1	Cellosaurus
		PTEN	0	Cellosaurus
		CDKN2AB	0	assumption
		MITF	2	Konieczkowski, D. J., et al. Cancer Discovery (2014)
UACC903	human	BRAFV600E	1	Byron, S. A., et al Mol Cancer (2012)
		PTEN	0	Byron, S. A., et al Mol Cancer (2012)
		CDKN2AB	0	Byron, S. A., et al Mol Cancer (2012)
		MITF	2	assumption
WM239	human	BRAFV600E	1	Cellosaurus
		PTEN	0	Cellosaurus
		CDKN2AB	0	assumption
		MITF	2	Hoek, K. S. et al. Pigm Cell Res (2006)

WM266-4	human	BRAFV600E	1	Cellosaurus
		PTEN	0	Cellosaurus
		CDKN2AB	0	assumption
		MITF	2	Konieczkowski, D. J., et al. Cancer Discovery (2014)
WM35	human	BRAFV600E	1	Byron, S. A., et al Mol Cancer (2012)
		CDKN2AB	0	Byron, S. A., et al Mol Cancer (2012)
		MITF	2	assumption
YUMM1.7	mouse	BRAFV600E	1	Meeth, K. et al. PC&MR (2016)
		PTEN	0	Meeth, K. et al. PC&MR (2016)
		CDKN2AB	0	Meeth, K. et al. PC&MR (2016)
		MITF	2	assumption

Supplementary Table S13. Genetic backgrounds of cell lines simulated. When conflicting information exists, both sources are provided, with the version used indicated in bold.

Supplementary Table S14. Constraints used for specification testing of Langerhans cell model

Node	Activity
CBF-B	1
CSF1	0
DKK	1
Gas6	1
IL-18	0
IL-34	1
Irf4	1
Pros1	1
TGF-B	1
WNT	1

Supplementary Table S15. Constraints used for specification testing of melanoma model

Node	Activity
a-MSH	0
APC	1
BRAFamplification	0
BRAFi	0
BRAFV600E	0
CDKN2AB	1
CellCellContact	1
DKK	1
ERKi	0
GF	1
MEKGoF	0
MEKi	0
MITF	2
NF1	1
ReplicationStress	1
TNF-a	0
WNT	1

Supplementary Table S16. Constraints used for specification testing of melanoma-LC model

Node	Activity
a-MSH	0
APC	1
BRAFamplification	0
BRAFi	0
BRAFV600E	0
CBF-B	1
CDKN2AB	1
CellCellContact	1
CSF1	0
DKK	1
ERKi	0
Gas6	1
GF	1
IL-18	0
IL-34	1
Irf4	1
MEKGoF	0
MEKi	0
MITF	2
NF1	1
Pros1	1
ReplicationStress	1
Sp1	1
WNT	1

REFERENCES AND NOTES

1. V. W. Rebecca, R. Somasundaram, M. Herlyn, Pre-clinical modeling of cutaneous melanoma. *Nat. Commun.* **11**, 2858 (2020).

2. R. L. Siegel, K. D. Miller, H. E. Fuchs, A. Jemal, Cancer statistics, 2022. *CA Cancer J. Clin.* **72**, 7–33 (2022).

3. M. A. El Sharouni, A. J. Witkamp, V. Sigurdsson, P. J. Van Diest, Comparison of survival between patients with single vs. multiple primary cutaneous melanomas. *JAMA Dermatol.* **155**, 1049–1056 (2019).

4. S. Kalaora, A. Nagler, J. A. Wargo, Y. Samuels, Mechanisms of immune activation and regulation: Lessons from melanoma. *Nat. Rev. Cancer* **22**, 195–207 (2022).

5. B. D. Koster, M. F. C. M. van den Hout, B. J. R. Sluijter, B. G. Molenkamp, R. J. C. L. M. Vuylsteke, A. Baars, P. A. M. van Leeuwen, R. J. Scheper, M. P. van den Tol, A. J. M. van den Eertwegh, T. D. de Gruijl, Local adjuvant treatment with low-dose CpG-B offers durable protection against disease recurrence in clinical stage I–II melanoma: Data from two randomized phase II trials. *Clin. Cancer Res.* **23**, 5679–5686 (2017).

6. W. Pruessmann, J. Rytlewski, J. Wilmott, M. C. Mihm Jr, G. H. Attrill, B. Dyring-Andersen, P. Fields, Q. Zhan, A. J. Colebatch, P. M. Ferguson, J. F. Thompson, K. Kallenbach, E. Yusko, R. A. Clark, H. Robins, R. A. Scolyer, T. S. Kupper, Molecular analysis of primary melanoma T cells identifies patients at risk for metastatic recurrence. *Nat. Cancer* **1**, 197–209 (2020).

7. D. H. Kaplan, Ontogeny and function of murine epidermal Langerhans cells. *Nat. Immunol.* **18**, 1068–1075 (2017).

8. K. Clayton, A. F. Vallejo, J. Davies, S. Sirvent, M. E. Polak, Langerhans cells—Programmed by the epidermis. *Front. Immunol.* **8**, 1676 (2017).

9. S. Sirvent, A. F. Vallejo, J. Davies, K. Clayton, Z. Wu, J. Woo, J. Riddell, V. K. Chaudhri, P. Stumpf, L. A. Nazlamova, G. Wheway, M. Rose-Zerilli, J. West, M. Pujato, X. Chen, C. H. Woelk, B. MacArthur, M. Ardern-Jones, P. S. Friedmann, M. T. Weirauch, H. Singh, M. E. Polak, Genomic programming of IRF4-expressing human Langerhans cells. *Nat. Commun.* **11**, 313 (2020).

10. J. Mohammed, L. K. Beura, A. Bobr, B. Astry, B. Chicoine, S. W. Kashem, N. E. Welty, B. Z. Igyártó, S. Wijeyesinghe, E. A. Thompson, C. Matte, L. Bartholin, A. Kaplan, D. Sheppard, A. G. Bridges, W. D. Shlomchik, D. Masopust, D. H. Kaplan, Stromal cells control the epithelial residence of DCs and memory T cells by regulated activation of TGF-β. *Nat. Immunol.* **17**, 414–421 (2016).

11. J. Davies, A. F. Vallejo, S. Sirvent, G. Porter, K. Clayton, Y. Qumbelo, P. Stumpf, J. West, C. M. Gray, N. T. L. Chigorimbo-Murefu, B. MacArthur, M. E. Polak, An IRF1-IRF4 toggle-switch controls tolerogenic and immunogenic transcriptional programming in human Langerhans cells. *Front. Immunol.* **12**, 665312 (2021).

12. R. van de Ven, M. F. C. M. van den Hout, J. J. Lindenberg, B. J. R. Sluijter, P. A. M. van Leeuwen, S. M. Lougheed, S. Meijer, M. P. van den Tol, R. J. Scheper, T. D. de Gruijl, Characterization of four conventional dendritic cell subsets in human skin-draining lymph nodes in relation to T-cell activation. *Blood* **118**, 2502–2510 (2011).

13. G. Gerlini, P. di Gennaro, G. Mariotti, C. Urso, A. Chiarugi, R. Caporale, N. Pimpinelli, L. Borgognoni, Human Langerhans cells are immature in melanoma sentinel lymph nodes. *Blood* **119**, 4807–4808 (2012).

14. M. F. C. M. van den Hout, B. D. Koster, B. J. R. Sluijter, P. A. M. van Leeuwen, S. Meijer, M. P. van den Tol, A. J. M. van den Eertwegh, R. J. Scheper, R. van de Ven, T. D. de Gruijl, Response: Breslow thickness and excision interval affect the activation state of Langerhans cells in melanoma sentinel lymph nodes. *Blood* **119**, 4809–4810 (2012).

15. G. Gerlini, P. Di Gennaro, N. Pimpinelli, S. Sestini, L. Borgognoni, Tolerogenic IDO1^{+}CD83^{-} Langerhans cells in sentinel lymph nodes of patients with melanoma. *Int. J. Mol. Sci.* **23**, 3441 (2022).

16. M. R. Romoli, P. Di Gennaro, G. Gerlini, S. Sestini, P. Brandani, S. Ferrone, L. Borgognoni, High antigen processing machinery component expression in Langerhans cells from melanoma patients' sentinel lymph nodes. *Cell Immunol.* **320**, 29–37 (2017).

17. F. Maria Bosisio, A. Antoranz, Y. van Herck, M. M. Bolognesi, L. Marcelis, C. Chinello, J. Wouters, F. Magni, L. Alexopoulos, M. Stas, V. Boecxstaens, O. Bechter, G. Cattoretti, J. van den Oord, Functional heterogeneity of lymphocytic patterns in primary melanoma dissected through single-cell multiplexing. *eLife* **9**, e53008 (2020).

18. A. J. Nirmal, Z. Maliga, T. Vallius, B. Quattrochi, A. A. Chen, C. A. Jacobson, R. J. Pelletier, C. Yapp, R. Arias-Camison, Y.A. Chen, C. G. Lian, G. F. Murphy, S. Santagata, P. K. Sorger, The spatial landscape of progression and immunoediting in primary melanoma at single-cell resolution. *Cancer Discov.* **12**, 1518–1541 (2022).

19. M. Albrecht, P. Lucarelli, D. Kulms, T. Sauter, Computational models of melanoma. *Theor. Biol. Med. Model.* **17**, 8 (2020).

20. S. J. Hamis, Y. Kapelyukh, A. McLaren, C. J. Henderson, C. Roland Wolf, M. A. J. Chaplain, Quantifying ERK activity in response to inhibition of the BRAFV600E-MEK-ERK cascade using mathematical modelling. *Br. J. Cancer* **125**, 1552–1560 (2021).

21. J. Béal, L. Pantolini, V. Noël, E. Barillot, L. Calzone, Personalized logical models to investigate cancer response to BRAF treatments in melanomas and colorectal cancers. *PLOS Comput. Biol.* **17**, e1007900 (2021).

22. F. Pappalardo, I. M. Forero, M. Pennisi, A. Palazon, I. Melero, S. Motta, SimB16: Modeling induced immune system response against B16-melanoma. *PLOS ONE* **6**, e26523 (2011).

23. N. Picco, E. Sahai, P. K. Maini, A. R. A. Anderson, Integrating models to quantify environment-mediated drug resistance. *Cancer Res.* **77**, 5409–5418 (2017).

24. R. Ruiz-Vega, C. F. Chen, E. Razzak, P. Vasudeva, T. B. Krasieva, J. Shiu, M. G. Caldwell, H. Yan, J. Lowengrub, A. K. Ganesan, A. D. Lander, Dynamics of nevus development implicate cell cooperation in the growth arrest of transformed melanocytes. *eLife* **9**, e61026 (2020).

25. J. Fisher, T. A. Henzinger, Executable cell biology. *Nat. Biotechnol.* **25**, 1239–1249 (2007).

26. M. A. Clarke, J. Fisher, Executable cancer models: Successes and challenges. *Nat. Rev. Cancer* **20**, 343–354 (2020).

27. R. Chuang, B. A. Hall, D. Benque, B. Cook, S. Ishtiaq, N. Piterman, A. Taylor, M. Vardi, S. Koschmieder, B. Gottgens, J. Fisher, Drug target optimization in chronic myeloid leukemia using innovative computational platform. *Sci. Rep.* **5**, 8190 (2015).

28. D. Silverbush, S. Grosskurth, D. Wang, F. Powell, B. Gottgens, J. Dry, J. Fisher, Cell-specific computational modeling of the PIM pathway in acute myeloid leukemia. *Cancer Res.* **77**, 827–838 (2017).

29. P. Kreuzaler, M. A. Clarke, E. J. Brown, C. H. Wilson, R. M. Kortlever, N. Piterman, T. Littlewood, G. I. Evan, J. Fisher, Heterogeneity of Myc expression in breast cancer exposes pharmacological vulnerabilities revealed through executable mechanistic modeling. *Proc. Natl. Acad. Sci. U.S.A.* **116**, 22399–22408 (2019).

30. R. Howell, M. A. Clarke, A.-K. Reuschl, T. Chen, S. Abbott-Imboden, M. Singer, D. M. Lowe, C. L. Bennett, B. Chain, C. Jolly, J. Fisher, Executable network of SARS-CoV-2-host interaction predicts drug combination treatments. *npj Digit. Med.* **5**, 18 (2022).

31. K. Meeth, J. X. Wang, G. Micevic, W. Damsky, M. W. Bosenberg, The YUMM lines: A series of congenic mouse melanoma cell lines with defined genetic alterations. *Pigment Cell Melanoma Res.* **29**, 590–597 (2016).

32. I. R. Ferrer, H. C. West, S. Henderson, D. S. Ushakov, P. Santos E Sousa, J. Strid, R. Chakraverty, A. J. Yates, C. L. Bennett, A wave of monocytes is recruited to replenish the long-term Langerhans cell network after immune injury. *Sci. Immunol.* **4**, eaax8704 (2019).

33. M. A. Schaub, T. A. Henzinger, J. Fisher, Qualitative networks: A symbolic approach to analyze biological signaling networks. *BMC Syst. Biol.* **1**, 4 (2007).

34. M. Cumberbatch, R. J. Dearman, I. Kimber, Langerhans cells require signals from both tumor necrosis factor α and interleukin 1β for migration. *Adv. Exp. Med. Biol.* **417**, 125–128 (1997).

35. S. Didovic, F. V. Opitz, B. Holzmann, I. Förster, H. Weighardt, Requirement of MyD88 signaling in keratinocytes for Langerhans cell migration and initiation of atopic dermatitis-like symptoms in mice. *Eur. J. Immunol.* **46**, 981–992 (2016).

36. M. P. Smith, B. Sanchez-Laorden, K. O'Brien, H. Brunton, J. Ferguson, H. Young, N. Dhomen, K. T. Flaherty, D. T. Frederick, Z. A. Cooper, J. A. Wargo, R. Marais, C. Wellbrock, The immune microenvironment confers resistance to MAPK pathway inhibitors through macrophage-derived TNF-α. *Cancer Discov.* **4**, 1214–1229 (2014).

37. P. Van Belle, U. Rodeck, I. Nuamah, A. C. Halpern, D. E. Elder, Melanoma-associated expression of transforming growth factor-beta isoforms. *Am. J. Pathol.* **148**, 1887–1894 (1996).

38. Y. Qin, S. Ekmekcioglu, P. Liu, L. M. Duncan, G. Lizée, N. Poindexter, E. A. Grimm, Constitutive aberrant endogenous interleukin-1 facilitates inflammation and growth in human melanoma. *Mol. Cancer Res.* **9**, 1537–1550 (2011).

39. S. Kuphal, S. Lodermeyer, F. Bataille, M. Schuierer, B. H. Hoang, A. K. Bosserhoff, Expression of Dickkopf genes is strongly reduced in malignant melanoma. *Oncogene* **25**, 5027–5036 (2006).

40. N. J. Neubert, M. Schmittnaegel, N. Bordry, S. Nassiri, N. Wald, C. Martignier, L. Tillé, K. Homicsko, W. Damsky, H. Maby-El Hajjami, I. Klaman, E. Danenberg, K. Ioannidou, L. Kandalaft, G. Coukos, S. Hoves, C. H. Ries, S. A. Fuertes Marraco, P. G. Foukas, M. De Palma, D. E. Speiser, T cell–induced CSF1 promotes melanoma resistance to PD1 blockade. *Sci. Transl. Med.* **10**, eaan3311 (2018).

41. N. Han, M. Baghdadi, K. Ishikawa, H. Endo, T. Kobayashi, H. Wada, K. Imafuku, H. Hata, K.I. Seino, Enhanced IL-34 expression in nivolumab-resistant metastatic melanoma. *Inflamm. Regen.* **38**, 3 (2018).

42. M. Sensi, M. Catani, G. Castellano, G. Nicolini, F. Alciato, G. Tragni, G. de Santis, I. Bersani, G. Avanzi, A. Tomassetti, S. Canevari, A. Anichini, Human cutaneous melanomas lacking MITF and melanocyte differentiation antigens express a functional Axl receptor kinase. *J. Invest. Dermatol.* **131**, 2448–2457 (2011).

43. The Cancer Genome Atlas Network, Genomic classification of cutaneous melanoma. *Cell* **161**, 1681–1696 (2015).

44. F. Rambow, J.-C. Marine, C. R. Goding, Melanoma plasticity and phenotypic diversity: Therapeutic barriers and opportunities. *Genes Dev.* **33**, 1295–1318 (2019).

45. A. Montfort, C. Colacios, T. Levade, N. Andrieu-Abadie, N. Meyer, B. Ségui, The TNF paradox in cancer progression and immunotherapy. *Front. Immunol.* **10**, 1818 (2019).

46. J. V. Falvo, A. V. Tsytsykova, A. E. Goldfeld, Transcriptional control of the TNF gene. *Curr. Dir. Autoimmun.* **11**, 27–60 (2010).

47. B. Wasylyk, J. Hagman, A. Gutierrez-Hartmann, Ets transcription factors: Nuclear effectors of the Ras-MAP-kinase signaling pathway. *Trends Biochem. Sci.* **23**, 213–216 (1998).

48. S. Riesenberg, A. Groetchen, R. Siddaway, T. Bald, J. Reinhardt, D. Smorra, J. Kohlmeyer, M. Renn, B. Phung, P. Aymans, T. Schmidt, V. Hornung, I. Davidson, C. R. Goding, G. Jönsson, J. Landsberg, T. Tüting, M. Hölzel, MITF and c-Jun antagonism interconnects melanoma dedifferentiation with pro-inflammatory cytokine responsiveness and myeloid cell recruitment. *Nat. Commun.* **6**, 8755 (2015).

49. M. Kappelmann, A. Bosserhoff, S. Kuphal, AP-1/c-Jun transcription factors: Regulation and function in malignant melanoma. *Eur. J. Cell Biol.* **93**, 76–81 (2014).

50. E. M. Novak, M. Metzger, R. Chammas, M. da Costa, K. Dantas, C. Manabe, J. Pires, A. C. de Oliveira, S. P. Bydlowski, Downregulation of TNF-α and VEGF expression by Sp1 decoy oligodeoxynucleotides in mouse melanoma tumor. *Gene Ther.* **10**, 1992–1997 (2003).

51. B. M. N. Brinkman, J. B. Telliez, A. R. Schievella, L. L. Lin, A. E. Goldfeld, Engagement of tumor necrosis factor (TNF) receptor 1 leads to ATF-2- and p38 mitogen-activated protein kinase-dependent TNF-α gene expression. *J. Biol. Chem.* **274**, 30882–30886 (1999).

52. J. J. Tyson, B. Novák, Functional motifs in biochemical reaction networks. *Annu. Rev. Phys. Chem.* **61**, 219–240 (2010).

53. M. Das Thakur, F. Salangsang, A. S. Landman, W. R. Sellers, N. K. Pryer, M. P. Levesque, R. Dummer, M. McMahon, D. D. Stuart, Modelling vemurafenib resistance in melanoma reveals a strategy to forestall drug resistance. *Nature* **494**, 251–255 (2013).

54. G. Moriceau, W. Hugo, A. Hong, H. Shi, X. Kong, C. C. Yu, R. C. Koya, A. A. Samatar, N. Khanlou, J. Braun, K. Ruchalski, H. Seifert, J. Larkin, K. B. Dahlman, D. B. Johnson, A. Algazi, J. A. Sosman, A. Ribas, R. S. Lo, Tunable-combinatorial mechanisms of acquired resistance limit the efficacy of BRAF/MEK cotargeting but result in melanoma drug addiction. *Cancer Cell* **27**, 240–256 (2015).

55. Y. Su, W. Wei, L. Robert, M. Xue, J. Tsoi, A. Garcia-Diaz, B. Homet Moreno, J. Kim, R. H. Ng, J. W. Lee, R. C. Koya, B. Comin-Anduix, T. G. Graeber, A. Ribas, J. R. Heath, Single-cell analysis resolves the cell state transition and signaling dynamics associated with melanoma drug-induced resistance. *Proc. Natl. Acad. Sci. U.S.A.* **114**, 13679–13684 (2017).

56. F. Rambow, A. Rogiers, O. Marin-Bejar, S. Aibar, J. Femel, M. Dewaele, P. Karras, D. Brown, Y. H. Chang, M. Debiec-Rychter, C. Adriaens, E. Radaelli, P. Wolter, O. Bechter, R. Dummer, M. Levesque, A. Piris, D. T. Frederick, G. Boland, K. T. Flaherty, J. van den Oord, T. Voet, S. Aerts, A. W. Lund, J.-C. Marine, Toward minimal residual disease-directed therapy in melanoma. *Cell* **174**, 843–855.e19 (2018).

57. C. E. M. Griffiths, R. J. Dearman, M. Cumberbatch, I. Kimber, Cytokines and Langerhans cell mobilisation in mouse and man. *Cytokine* **32**, 67–70 (2005).

58. I. Arozarena, C. Wellbrock, Phenotype plasticity as enabler of melanoma progression and therapy resistance. *Nat. Rev. Cancer* **19**, 377–391 (2019).

59. K. L. Walton, K. E. Johnson, C. A. Harrison, Targeting TGF-β–mediated SMAD signaling for the prevention of fibrosis. *Front. Pharmacol.* **8**, 461 (2017).

60. P. M. Wojnarowicz, R. Lima E Silva, M. Ohnaka, S. B. Lee, Y. Chin, A. Kulukian, S.-H. Chang, B. Desai, M. G. Escolano, R. Shah, M. Garcia-Cao, S. Xu, R. Kadam, Y. Goldgur, M. A. Miller, O. Ouerfelli, G. Yang, T. Arakawa, S. K. Albanese, W. A. Garland, G. Stoller, J. Chaudhary, L. Norton, R. K. Soni, J. Philip, R. C. Hendrickson, A. Iavarone, A. J. Dannenberg, J. D. Chodera, N. Pavletich, A. Lasorella, P. A. Campochiaro, R. Benezra, A small-molecule Pan-Id antagonist inhibits pathologic ocular neovascularization. *Cell Rep.* **29**, 62–75.e7 (2019).

61. M. Chopin, C. Seillet, S. Chevrier, L. Wu, H. Wang, H. C. Morse III, G. T. Belz, S. L. Nutt, Langerhans cells are generated by two distinct PU.1-dependent transcriptional networks. *J. Exp. Med.* **210**, 2967–2980 (2013).

62. J. M. Kel, M. J. H. Girard-Madoux, B. Reizis, B. E. Clausen, TGF-β Is required to maintain the pool of immature Langerhans cells in the epidermis. *J. Immunol.* **185**, 3248–3255 (2010).

63. I. Boned del Río, L. C. Young, S. Sari, G. G. Jones, B. Ringham-Terry, N. Hartig, E. Rejnowicz, W. Lei, A. Bhamra, S. Surinova, P. Rodriguez-Viciana, SHOC2 complex-driven RAF dimerization selectively contributes to ERK pathway dynamics. *Proc. Natl. Acad. Sci. U.S.A.* **116**, 13330–13339 (2019).

64. A. Paul, J. Edwards, C. Pepper, S. Mackay, Inhibitory-κB kinase (IKK) α and nuclear factor-κB (NFκB)-inducing kinase (NIK) as anti-cancer drug targets. *Cells* **7**, 176 (2018).

65. B. Vanhaesebroeck, M. W. D. Perry, J. R. Brown, F. André, K. Okkenhaug, PI3K inhibitors are finally coming of age. *Nat. Rev. Drug Discov.* **20**, 741–769 (2021).

66. F. Martorana, G. Motta, G. Pavone, L. Motta, S. Stella, S. R. Vitale, L. Manzella, P. Vigneri, AKT inhibitors: New weapons in the fight against breast cancer? *Front. Pharmacol.* **12**, 662232 (2021).

67. V. Tisato, R. Voltan, A. Gonelli, P. Secchiero, G. Zauli, MDM2/X inhibitors under clinical evaluation: Perspectives for the management of hematological malignancies and pediatric cancer. *J. Hematol. Oncol.* **10**, 133 (2017).

68. L. M. Spring, S. A. Wander, M. Zangardi, A. Bardia, CDK 4/6 inhibitors in breast cancer: Current controversies and future directions. *Curr. Oncol. Rep.* **21**, 25 (2019).

69. F. Rouaud, N. Hamouda-Tekaya, M. Cerezo, P. Abbe, J. Zangari, V. Hofman, M. Ohanna, B. Mograbi, N. el-Hachem, Z. Benfodda, A. Lebeau, M. K. Tulic, P. Hofman, C. Bertolotto, T. Passeron, J.S. Annicotte, R. Ballotti, S. Rocchi, E2F1 inhibition mediates cell death of metastatic melanoma. *Cell Death Dis.* **9**, 527 (2018).

70. O. Elsaeßer, U. Leiter, P. G. Buettner, T. K. Eigentler, F. Meier, B. Weide, G. Metzler, H. Breuninger, C. Garbe, Prognosis of sentinel node staged patients with primary cutaneous melanoma. *PLOS ONE* **7**, e29791 (2012).

71. B. A. Nguyen, J. Ho, J. S. De La Cruz Diaz, S. Nishimura, D. H. Kaplan, TGFβ activating integrins β6 and β8 are dysregulated in inflammatory skin disease and cutaneous melanoma. *J. Dermatol. Sci.* **106**, 2–11 (2022).

72. G. M. Halliday, S. Le, Transforming growth factor-beta produced by progessor tumors inhibits, while IL-10 produced by regressor tumors enhances, Langerhans cell migration from skin. *Int. Immunol.* **13**, 1147–1154 (2001).

73. E. Ubil, L. Caskey, A. Holtzhausen, D. Hunter, C. Story, H. S. Earp, Tumor-secreted Pros1 inhibits macrophage M1 polarization to reduce antitumor immune response. *J. Clin. Invest.* **128**, 2356–2369 (2018).

74. M. Tanaka, D. W. Siemann, Gas6/Axl signaling pathway in the tumor immune microenvironment. *Cancers* **12**, 1850 (2020).

75. Y. Tabib, N. S. Jaber, M. Nassar, T. Capucha, G. Mizraji, T. Nir, N. Koren, I. Aizenbud, A. Maimon, L. Eli-Berchoer, A. Wilensky, T. Burstyn-Cohen, A.-H. Hovav, Cell-intrinsic regulation of murine epidermal Langerhans cells by protein S. *Proc. Natl. Acad. Sci. U.S.A.* **115**, E5736–E5745 (2018).

76. N. K. Hayward, J. S. Wilmott, N. Waddell, P. A. Johansson, M. A. Field, K. Nones, A.-M. Patch, H. Kakavand, L. B. Alexandrov, H. Burke, V. Jakrot, S. Kazakoff, O. Holmes, C. Leonard, R. Sabarinathan, L. Mularoni, S. Wood, Q. Xu, N. Waddell, V. Tembe, G. M. Pupo, R. De Paoli-Iseppi, R. E. Vilain, P. Shang, L. M. S. Lau, R. A. Dagg, S.-J. Schramm, A. Pritchard, K. Dutton-Regester, F. Newell, A. Fitzgerald, C. A. Shang, S. M. Grimmond, H. A. Pickett, J. Y. Yang, J. R. Stretch, A. Behren, R. F. Kefford, P. Hersey, G. V. Long, J. Cebon, M. Shackleton, A. J. Spillane, R. P. M. Saw, N. López-Bigas, J. V. Pearson, J. F. Thompson, R. A. Scolyer, G. J. Mann, Whole-genome landscapes of major melanoma subtypes. *Nature* **545**, 175–180 (2017).

77. A. H. Shain, N. M. Joseph, R. Yu, J. Benhamida, S. Liu, T. Prow, B. Ruben, J. North, L. Pincus, I. Yeh, R. Judson, B. C. Bastian, Genomic and transcriptomic analysis reveals incremental disruption of key signaling pathways during melanoma evolution. *Cancer Cell* **34**, 45–55.e4 (2018).

78. A. H. Shain, B. C. Bastian, From melanocytes to melanomas. *Nat. Rev. Cancer* **16**, 345–358 (2016).

79. D. Schadendorf, D. E. Fisher, C. Garbe, J. E. Gershenwald, J.-J. Grob, A. Halpern, M. Herlyn, M. A. Marchetti, G. M. Arthur, A. Ribas, A. Roesch, A. Hauschild, Melanoma. *Nat. Rev. Dis. Prim.* **1**, 15003 (2015).

80. J. J. Lee, L. M. Sholl, N. I. Lindeman, S. R. Granter, A. C. Laga, P. Shivdasani, G. Chin, J. J. Luke, P. A. Ott, F. S. Hodi, M. C. Mihm Jr, J. Y. Lin, A. E. Werchniak, H. A. Haynes, N. Bailey, R. Liu, G. F. Murphy, C. G. Lian, Targeted next-generation sequencing reveals high frequency of mutations in epigenetic regulators across treatment-naïve patient melanomas. *Clin. Epigenetics* **7**, 59 (2015).

81. K. Preuer, R. P. I. Lewis, S. Hochreiter, A. Bender, K. C. Bulusu, G. Klambauer, DeepSynergy: Predicting anti-cancer drug synergy with deep learning. *Bioinformatics* **34**, 1538–1546 (2018).

82. F. Xia, M. Shukla, T. Brettin, C. Garcia-Cardona, J. Cohn, J. E. Allen, S. Maslov, S. L. Holbeck, J. H. Doroshow, Y. A. Evrard, E. A. Stahlberg, R. L. Stevens, Predicting tumor cell line response to drug pairs with deep learning. *BMC Bioinformatics* **19**, 486 (2018).

83. M. Garrido-Rodriguez, K. Zirngibl, O. Ivanova, S. Lobentanzer, J. Saez-Rodriguez, Integrating knowledge and omics to decipher mechanisms via large-scale models of signaling networks. *Mol. Syst. Biol.* **18**, e11036 (2022).

84. A. Garg, A. Di Cara, I. Xenarios, L. Mendoza, G. De Micheli, Synchronous versus asynchronous modeling of gene regulatory networks. *Bioinformatics* **24**, 1917–1925 (2008).

85. J. Fisher, N. Piterman, A. Hajnal, T. A. Henzinger, Predictive modeling of signaling crosstalk during C. elegans vulval development. *PLOS Comput. Biol.* **3**, e92 (2007).

86. V. Moignard, S. Woodhouse, L. Haghverdi, A. J. Lilly, Y. Tanaka, A. C. Wilkinson, F. Buettner, I. C. Macaulay, W. Jawaid, E. Diamanti, S.I. Nishikawa, N. Piterman, V. Kouskoff, F. J. Theis, J. Fisher, B. Göttgens, Decoding the regulatory network of early blood development from single-cell gene expression measurements. *Nat. Biotechnol.* **33**, 269–276 (2015).

87. M. A. Clarke, S. Woodhouse, N. Piterman, B. A. Hall, J. Fisher, Using state space exploration to determine how gene regulatory networks constrain mutation order in cancer evolution, in *Automated Reasoning for Systems Biology and Medicine*, P. Liò, P. Zuliani, Eds. (Springer International Publishing, 2019), pp. 133–153.

88. S. Bornholdt, Boolean network models of cellular regulation: Prospects and limitations. *J. R. Soc. Interface* **5** (suppl. 1), S85–S94 (2008).

89. D. Benque, S. Bourton, C. Cockerton, B. Cook, J. Fisher, S. Ishtiaq, N. Piterman, A. Taylor, M. Y. Vardi, BMA: Visual tool for modeling and analyzing biological networks, in *Computer*

Aided Verification. CAV 2012 (Springer, 2012), Lecture Notes in Computer Science (including Subseries Lecture Notes in Artificial Intelligence and Lecture Notes in Bioinformatics), vol. 7358 LNCS, pp. 686–692.

90. A. Ribas, K. T. Flaherty, BRAF targeted therapy changes the treatment paradigm in melanoma. *Nat. Rev. Clin. Oncol.* **8**, 426–433 (2011).

91. B. Cook, J. Fisher, E. Krepska, N. Piterman, Proving stabilization of biological systems, in *Verification, Model Checking, and Abstract Interpretation. VMCAI 2011* (Springer, 2011), Lecture Notes in Computer Science (including Subseries Lecture Notes in Artificial Intelligence and Lecture Notes in Bioinformatics), vol. 6538 LNCS, pp. 134–149.

92. R Core Team, R: A Language and Environment for Statistical Computing (2020).

93. R. Kolde, pheatmap: Pretty Heatmaps (2019).

94. E. Neuwirth, RColorBrewer: ColorBrewer Palettes (2014).

95. G. Yu, ggplotify: Convert Plot to “grob” or “ggplot” Object (2021).

96. H. Wickham, M. Averick, J. Bryan, W. Chang, L. McGowan, R. François, G. Grolemund, A. Hayes, L. Henry, J. Hester, M. Kuhn, T. Pedersen, E. Miller, S. Bache, K. Müller, J. Ooms, D. Robinson, D. Seidel, V. Spinu, K. Takahashi, D. Vaughan, C. Wilke, K. Woo, H. Yutani, Welcome to the Tidyverse. *J. Open Source Softw.* **4**, 1686 (2019).

97. J. Deckers, H. Hammad, E. Hoste, Langerhans cells: Sensing the environment in health and disease. *Front. Immunol.* **9**, 93 (2018).

98. K. Seré, J. H. Baek, J. Ober-Blöbaum, G. Müller-Newen, F. Tacke, Y. Yokota, M. Zenke, T. Hieronymus, Two distinct types of Langerhans cells populate the skin during steady state and inflammation. *Immunity* **37**, 905–916 (2012).

99. F. Sparber, C. H. Tripp, K. Komenda, J. M. Scheffler, B. E. Clausen, L. A. Huber, N. Romani, P. Stoitzner, The late endosomal adaptor molecule p14 (LAMTOR2) regulates TGFβ1-mediated homeostasis of Langerhans cells. *J. Invest. Dermatol.* **135**, 119–129 (2015).

100. T. Hieronymus, M. Zenke, J.-H. Baek, K. Seré, The clash of Langerhans cell homeostasis in skin: Should I stay or should I go? *Semin. Cell Dev. Biol.* **41**, 30–38 (2015).

101. N. Yasmin, S. Konradi, G. Eisenwort, Y. M. Schichl, M. Seyerl, T. Bauer, J. Stöckl, H. Strobl, β-Catenin promotes the differentiation of epidermal Langerhans dendritic cells. *J. Invest. Dermatol.* **133**, 1250–1259 (2013).

102. Y. Wang, K. J. Szretter, W. Vermi, S. Gilfillan, C. Rossini, M. Cella, A. D. Barrow, M. S. Diamond, M. Colonna, IL-34 is a tissue-restricted ligand of CSF1R required for the development of Langerhans cells and microglia. *Nat. Immunol.* **13**, 753–760 (2012).

103. T. Bauer, A. Zagórska, J. Jurkin, N. Yasmin, R. Köffel, S. Richter, B. Gesslbauer, G. Lemke, H. Strobl, Identification of Axl as a downstream effector of TGF-β1 during Langerhans cell differentiation and epidermal homeostasis. *J. Exp. Med.* **209**, 2033–2047 (2012).

104. M. R. Becker, Y. S. Choi, S. E. Millar, M. C. Udey, Wnt signaling influences the development of murine epidermal Langerhans cells. *J. Invest. Dermatol.* **131**, 1861–1868 (2011).

105. L. Galluzzi, S. Spranger, E. Fuchs, A. López-Soto, WNT signaling in cancer immunosurveillance. *Trends Cell Biol.* **29**, 44–65 (2019).

106. M. Chopin, S. L. Nutt, Establishing and maintaining the Langerhans cell network. *Semin. Cell Dev. Biol.* **41**, 23–29 (2015).

107. J. S. De La Cruz Diaz, T. Hirai, B. A.-T. Nguyen, Y. Zenke, Y. Yang, H. Li, S. Nishimura, D. H. Kaplan, TNF-α and IL-1β do not induce Langerhans cell migration by inhibiting TGFβ activation. *JID Innov.* **1**, 100028 (2021).

108. M. H. Orzalli, A. Smith, K. A. Jurado, A. Iwasaki, J. A. Garlick, J. C. Kagan, An antiviral branch of the IL-1 signaling pathway restricts immune-evasive virus replication. *Mol. Cell* **71**, 825–840.e6 (2018).

109. A. Tang, M. Amagai, L. G. Granger, J. R. Stanley, M. C. Uddy, Adhesion of epidermal Langerhans cells to keratinocytes mediated by E-cadherin. *Nature* **361**, 82–85 (1993).

110. C. Scatena, D. Murtas, S. Tomei, Cutaneous melanoma classification: The importance of high-throughput genomic technologies. *Front. Oncol.* **11**, 635488 (2021).

111. V. B. Swope, E. E. Medrano, D. Smalara, Z. A. Abdel-Malek, Long-term proliferation of human melanocytes is supported by the physiologic mitogens α-melanotropin, endothelin-1, and basic fibroblast growth factor. *Exp. Cell Res.* **217**, 453–459 (1995).

112. C. Sun, L. Wang, S. Huang, G. J. J. E. Heynen, A. Prahallad, C. Robert, J. Haanen, C. Blank, J. Wesseling, S. M. Willems, D. Zecchin, S. Hobor, P. K. Bajpe, C. Lieftink, C. Mateus, S. Vagner, W. Grernrum, I. Hofland, A. Schlicker, L. F. A. Wessels, R. L. Beijersbergen, A. Bardelli, F. di Nicolantonio, A. M. M. Eggermont, R. Bernards, Reversible and adaptive resistance to BRAF(V600E) inhibition in melanoma. *Nature* **508**, 118–122 (2014).

113. D. J. Easty, S. G. Gray, K. J. O'Byrne, D. O'Donnell, D. C. Bennett, Receptor tyrosine kinases and their activation in melanoma. *Pigment Cell Melanoma Res.* **24**, 446–461 (2011).

114. P. Lito, C. A. Pratilas, E. W. Joseph, M. Tadi, E. Halilovic, M. Zubrowski, A. Huang, W. L. Wong, M. K. Callahan, T. Merghoub, J. D. Wolchok, E. de Stanchina, S. Chandarlapaty, P. I. Poulikakos, J. A. Fagin, N. Rosen, Relief of profound feedback inhibition of mitogenic signaling by RAF inhibitors attenuates their activity in BRAFV600E melanomas. *Cancer Cell* **22**, 668–682 (2012).

115. D. J. Konieczkowski, C. M. Johannessen, O. Abudayyeh, J. W. Kim, Z. A. Cooper, A. Piris, D. T. Frederick, M. Barzily-Rokni, R. Straussman, R. Haq, D. E. Fisher, J. P. Mesirov, W. C. Hahn, K. T. Flaherty, J. A. Wargo, P. Tamayo, L. A. Garraway, A melanoma cell state

distinction influences sensitivity to MAPK pathway inhibitors. *Cancer Discov.* **4**, 816–827 (2014).

116. C. A. Worby, J. E. Dixon, PTEN. *Annu. Rev. Biochem.* **83**, 641–669 (2014).

117. D. C. Bennett, Human melanocyte senescence and melanoma susceptibility genes. *Oncogene* **22**, 3063–3069 (2003).

118. A. S. McNeal, K. Liu, V. Nakhate, C. A. Natale, E. K. Duperret, B. C. Capell, T. Dentchev, S. L. Berger, M. Herlyn, J. T. Seykora, T. W. Ridky, CDKN2B loss promotes progression from benign melanocytic nevus to melanoma. *Cancer Discov.* **5**, 1072–1085 (2015).

119. M. P. O'Connell, A. T. Weeraratna, Hear the Wnt Ror: How melanoma cells adjust to changes in Wnt. *Pigment Cell Melanoma Res.* **22**, 724–739 (2009).

120. C. Herraiz, J. C. Garcia-Borron, C. Jiménez-Cervantes, C. Olivares, MC1R signaling. Intracellular partners and pathophysiological implications. *Biochim. Biophys. Acta Mol. Basis Dis.* **1863**, 2448–2461 (2017).

121. A. Mehta, Y. J. Kim, L. Robert, J. Tsoi, B. Comin-Anduix, B. Berent-Maoz, A. J. Cochran, J. S. Economou, P. C. Tumeh, C. Puig-Saus, A. Ribas, Immunotherapy resistance by inflammation-induced dedifferentiation. *Cancer Discov.* **8**, 935–943 (2018).

122. V. C. Gray-Schopfer, M. Karasarides, R. Hayward, R. Marais, Tumor necrosis factor-α blocks apoptosis in melanoma cells when BRAF signaling is inhibited. *Cancer Res.* **67**, 122–129 (2007).

123. G. Sabio, R. J. Davis, TNF and MAP kinase signalling pathways. *Semin. Immunol.* **26**, 237–245 (2014).

124. K. S. Hoek, N. C. Schlegel, P. Brafford, A. Sucker, S. Ugurel, R. Kumar, B. L. Weber, K. L. Nathanson, D. J. Phillips, M. Herlyn, D. Schadendorf, R. Dummer, Metastatic potential of melanomas defined by specific gene expression profiles with no BRAF signature. *Pigment Cell Res.* **19**, 290–302 (2006).

125. D. Javelaud, V. I. Alexaki, M. J. Pierrat, K. S. Hoek, S. Dennler, L. van Kempen, C. Bertolotto, R. Ballotti, S. Saule, V. Delmas, A. Mauviel, GLI2 and M-MITF transcription factors control exclusive gene expression programs and inversely regulate invasion in human melanoma cells. *Pigment Cell Melanoma Res.* **24**, 932–943 (2011).

126. C. Levy, M. Khaled, D. E. Fisher, MITF: Master regulator of melanocyte development and melanoma oncogene. *Trends Mol. Med.* **12**, 406–414 (2006).

127. M. Johannessen, U. Moens, Multisite phosphorylation of the cAMP response element-binding protein CREB by a diversity of protein kinases. *Front. Biosci.* **12**, 1814–1832 (2007).

128. H. Zhang, Q. Kong, J. Wang, Y. Jiang, H. Hua, Complex roles of cAMP–PKA–CREB signaling in cancer. *Exp. Hematol. Oncol.* **9**, 32 (2020).

129. M. J. Pierrat, V. Marsaud, A. Mauviel, D. Javelaud, Expression of microphthalmia-associated transcription factor (MITF), which is critical for melanoma progression, is inhibited by both transcription factor GLI2 and transforming growth factor-β. *J. Biol. Chem.* **287**, 17996–18004 (2012).

130. A. Kaur, M. R. Webster, A. T. Weeraratna, In the Wnt-er of life: Wnt signalling in melanoma and ageing. *Br. J. Cancer* **115**, 1273–1279 (2016).

131. M. P. O’Connell, K. Marchbank, M. R. Webster, A. A. Valiga, A. Kaur, A. Vultur, L. Li, M. Herlyn, J. Villanueva, Q. Liu, X. Yin, S. Widura, J. Nelson, N. Ruiz, T. C. Camilli, F. E. Indig, K. T. Flaherty, J. A. Wargo, D. T. Frederick, Z. A. Cooper, S. Nair, R. K. Amaravadi, L. M. Schuchter, G. C. Karakousis, W. Xu, X. Xu, A. T. Weeraratna, Hypoxia induces phenotypic plasticity and therapy resistance in melanoma via the tyrosine kinase receptors ROR1 and ROR2. *Cancer Discov.* **3**, 1378–1393 (2013).

132. S. K. Dissanayake, P. B. Olkhanud, M. P. O'Connell, A. Carter, A. D. French, T. C. Camilli, C. D. Emeche, K. J. Hewitt, D. T. Rosenthal, P. D. Leotlela, M. S. Wade, S. W. Yang, L. Brant, B. J. Nickoloff, J. L. Messina, A. Biragyn, K. S. Hoek, D. D. Taub, D. L. Longo, V. K. Sondak, S. M. Hewitt, A. T. Weeraratna, Wnt5A regulates expression of tumor-associated

antigens in melanoma via changes in signal transducers and activators of transcription 3 phosphorylation. *Cancer Res.* **68**, 10205–10214 (2008).

133. V. Delmas, F. Beermann, S. Martinozzi, S. Carreira, J. Ackermann, M. Kumasaka, L. Denat, J. Goodall, F. Luciani, A. Viros, N. Demirkan, B. C. Bastian, C. R. Goding, L. Larue, β-Catenin induces immortalization of melanocytes by suppressing p16INK4a expression and cooperates with N-Ras in melanoma development. *Genes Dev.* **21**, 2923–2935 (2007).

134. W. E. Damsky, D. P. Curley, M. Santhanakrishnan, L. E. Rosenbaum, J. T. Platt, B. E. G. Rothberg, M. M. Taketo, D. Dankort, D. L. Rimm, M. M. Mahon, M. Bosenberg, β-Catenin signaling controls metastasis in Braf-activated Pten-deficient melanomas. *Cancer Cell* **20**, 741–754 (2011).

135. A. J. Chien, E. C. Moore, A. S. Lonsdorf, R. M. Kulikauskas, B. G. Rothberg, A. J. Berger, M. B. Major, S. T. Hwang, D. L. Rimm, R. T. Moon, Activated Wnt/ß-catenin signaling in melanoma is associated with decreased proliferation in patient tumors and a murine melanoma model. *Proc. Natl. Acad. Sci. U.S.A.* **106**, 1193–1198 (2009).

136. H. R. Widlund, M. A. Horstmann, E R. Price, J. Cui, S. L. Lessnick, M. Wu, X. He, D. E. Fisher, β-Catenin–induced melanoma growth requires the downstream target Microphthalmia-associated transcription factor. *J. Cell Biol.* **158**, 1079–1087 (2002).

137. L. A. Garraway, H. R. Widlund, M. A. Rubin, G. Getz, A. J. Berger, S. Ramaswamy, R. Beroukhim, D. A. Milner, S. R. Granter, J. Du, C. Lee, S. N. Wagner, C. Li, T. R. Golub, D. L. Rimm, M. L. Meyerson, D. E. Fisher, W. R. Sellers, Integrative genomic analyses identify MITF as a lineage survival oncogene amplified in malignant melanoma. *Nature* **436**, 117–122 (2005).

138. S. Carreira, J. Goodall, I. Aksan, S. A. la Rocca, M.D. Galibert, L. Denat, L. Larue, C. R. Goding, Mitf cooperates with Rb1 and activates p21Cip1 expression to regulate cell cycle progression. *Nature* **433**, 764–769 (2005).

139. L. Ha, T. Ichikawa, M. Anver, R. Dickins, S. Lowe, N. E. Sharpless, P. Krimpenfort, R. A. DePinho, D. C. Bennett, E. V. Sviderskaya, G. Merlino, ARF functions as a melanoma tumor suppressor by inducing p53-independent senescence. *Proc. Natl. Acad. Sci. U.S.A.* **104**, 10968–10973 (2007).

140. C. Michaloglou, L. C. W. Vredeveld, M. S. Soengas, C. Denoyelle, T. Kuilman, C. M. A. M. van der Horst, D. M. Majoor, J. W. Shay, W. J. Mooi, D. S. Peeper, BRAFE600-associated senescence-like cell cycle arrest of human naevi. *Nature* **436**, 720–724 (2005).

141. N. Dhomen, J. S. Reis-Filho, S. da Rocha Dias, R. Hayward, K. Savage, V. Delmas, L. Larue, C. Pritchard, R. Marais, Oncogenic Braf induces melanocyte senescence and melanoma in mice. *Cancer Cell* **15**, 294–303 (2009).

142. L. L. Scurr, G. M. Pupo, T. M. Becker, K. Lai, D. Schrama, S. Haferkamp, M. Irvine, R. A. Scolyer, G. J. Mann, J. C. Becker, R. F. Kefford, H. Rizos, IGFBP7 is not required for B-RAF-induced melanocyte senescence. *Cell* **141**, 717–727 (2010).

143. M. Verhaegen, J. A. Bauer, C. Martín de la Vega, G. Wang, K. G. Wolter, J. C. Brenner, Z. Nikolovska-Coleska, A. Bengtson, R. Nair, J. T. Elder, M. van Brocklin, T. E. Carey, C. R. Bradford, S. Wang, Ḿ. S. Soengas, A novel BH3 mimetic reveals a mitogen-activated protein kinase–dependent mechanism of melanoma cell death controlled by p53 and reactive oxygen species. *Cancer Res.* **66**, 11348–11359 (2006).

144. C. R. Goding, H. Arnheiter, MITF—The first 25 years. *Genes Dev.* **33**, 983–1007 (2019).

145. K. S. Hoek, O. M. Eichhoff, N. C. Schlegel, U. Döbbeling, N. Kobert, L. Schaerer, S. Hemmi, R. Dummer, In vivo switching of human melanoma cells between proliferative and invasive states. *Cancer Res.* **68**, 650–656 (2008).

146. I. Tirosh, B. Izar, S. M. Prakadan, M. H. Wadsworth II, D. Treacy, J. J. Trombetta, A. Rotem, C. Rodman, C. Lian, G. Murphy, M. Fallahi-Sichani, K. Dutton-Regester, J.R. Lin, O. Cohen, P. Shah, D. Lu, A. S. Genshaft, T. K. Hughes, C. G. K. Ziegler, S. W. Kazer, A. Gaillard, K. E. Kolb, A.C. Villani, C. M. Johannessen, A. Y. Andreev, E. M. van Allen, M.

Bertagnolli, P. K. Sorger, R. J. Sullivan, K. T. Flaherty, D. T. Frederick, J. Jané-Valbuena, C. H. Yoon, O. Rozenblatt-Rosen, A. K. Shalek, A. Regev, L. A. Garraway, Dissecting the multicellular ecosystem of metastatic melanoma by single-cell RNA-seq. *Science* **352**, 189–196 (2016).

147. S. M. Shaffer, M. C. Dunagin, S. R. Torborg, E. A. Torre, B. Emert, C. Krepler, M. Beqiri, K. Sproesser, P. A. Brafford, M. Xiao, E. Eggan, I. N. Anastopoulos, C. A. Vargas-Garcia, A. Singh, K. L. Nathanson, M. Herlyn, A. Raj, Rare cell variability and drug-induced reprogramming as a mode of cancer drug resistance. *Nature* **546**, 431–435 (2017).

148. J. Tsoi, L. Robert, K. Paraiso, C. Galvan, K. M. Sheu, J. Lay, D. J. L. Wong, M. Atefi, R. Shirazi, X. Wang, D. Braas, C. S. Grasso, N. Palaskas, A. Ribas, T. G. Graeber, Multi-stage differentiation defines melanoma subtypes with differential vulnerability to drug-induced iron-dependent oxidative stress. *Cancer Cell* **33**, 890–904.e5 (2018).

149. J. Wouters, Z. Kalender-Atak, L. Minnoye, K. I. Spanier, M. De Waegeneer, C. B. González-Blas, D. Mauduit, K. Davie, G. Hulselmans, A. Najem, M. Dewaele, D. Pedri, F. Rambow, S. Makhzami, V. Christiaens, F. Ceyssens, G. Ghanem, J.-C. Marine, S. Poovathingal, S. Aerts, Robust gene expression programs underlie recurrent cell states and phenotype switching in melanoma. *Nat. Cell Biol.* **22**, 986–998 (2020).

150. M. Fallahi-Sichani, N. J. Moerke, M. Niepel, T. Zhang, N. S. Gray, P. K. Sorger, Systematic analysis of BRAF^{V600E} melanomas reveals a role for JNK/c-Jun pathway in adaptive resistance to drug-induced apoptosis. *Mol. Syst. Biol.* **11**, 797 (2015).

151. W. E. Damsky, M. Bosenberg, Melanocytic nevi and melanoma: Unraveling a complex relationship. *Oncogene* **36**, 5771–5792 (2017).

152. L. C. W. Vredeveld, P. A. Possik, M. A. Smit, K. Meissl, C. Michaloglou, H. M. Horlings, A. Ajouaou, P. C. Kortman, D. Dankort, M. McMahon, W. J. Mooi, D. S. Peeper, Abrogation of BRAFV600E-induced senescence by PI3K pathway activation contributes to melanomagenesis. *Genes Dev.* **26**, 1055–1069 (2012).

153. W. Damsky, G. Micevic, K. Meeth, V. Muthusamy, D. P. Curley, M. Santhanakrishnan, I. Erdelyi, J. T. Platt, L. Huang, N. Theodosakis, M. R. Zaidi, S. Tighe, M. A. Davies, D. Dankort, M. McMahon, G. Merlino, N. Bardeesy, M. Bosenberg, mTORC1 activation blocks BrafV600E-induced growth arrest but is insufficient for melanoma formation. *Cancer Cell* **27**, 41–56 (2015).

154. Y. F. Wang, C. C. Jiang, K. A. Kiejda, S. Gillespie, X. D. Zhang, P. Hersey, Apoptosis induction in human melanoma cells by inhibition of MEK is caspase-independent and mediated by the Bcl-2 family members PUMA, Bim, and Mcl-1. *Clin. Cancer Res.* **13**, 4934–4942 (2007).

155. S. V. Saladi, P. G. Wong, A. R. Trivedi, H. G. Marathe, B. Keenen, S. Aras, Z.-Q. Liew, V. Setaluri, I. L. de la Serna, BRG1 promotes survival of UV-irradiated melanoma cells by cooperating with MITF to activate the melanoma inhibitor of apoptosis gene. *Pigment Cell Melanoma Res.* **26**, 377–391 (2013).

156. C.-Y. Wang, M. W. Mayo, R. G. Korneluk, D. V. Goeddel, A. S. Baldwin Jr., NF-κB antiapoptosis: Induction of TRAF1 and TRAF2 and c-IAP1 and c-IAP2 to suppress caspase-8 activation. *Science* **281**, 1680–1683 (1998).

157. A. Hong, G. Moriceau, L. Sun, S. Lomeli, M. Piva, R. Damoiseaux, S. L. Holmen, N. E. Sharpless, W. Hugo, R. S. Lo, Exploiting drug addiction mechanisms to select against MAPKi-resistant melanoma. *Cancer Discov.* **8**, 74–93 (2018).

158. D. Zhuang, S. Mannava, V. Grachtchouk, W. H. Tang, S. Patil, J. A. Wawrzyniak, A. E. Berman, T. J. Giordano, E. V. Prochownik, M. S. Soengas, M. A. Nikiforov, C-MYC overexpression is required for continuous suppression of oncogene-induced senescence in melanoma cells. *Oncogene* **27**, 6623–6634 (2008).

159. D. B. Solit, L. A. Garraway, C. A. Pratilas, A. Sawai, G. Getz, A. Basso, Q. Ye, J. M. Lobo, Y. She, I. Osman, T. R. Golub, J. Sebolt-Leopold, W. R. Sellers, N. Rosen, BRAF mutation predicts sensitivity to MEK inhibition. *Nature* **439**, 358–362 (2006).

160. L. N. Kwong, J. C. Costello, H. Liu, S. Jiang, T. L. Helms, A. E. Langsdorf, D. Jakubosky, G. Genovese, F. L. Muller, J. H. Jeong, R. P. Bender, G. C. Chu, K. T. Flaherty, J. A. Wargo, J. J. Collins, L. Chin, Oncogenic NRAS signaling differentially regulates survival and proliferation in melanoma. *Nat. Med.* **18**, 1503–1510 (2012).

161. C. Posch, H. Moslehi, L. Feeney, G. A. Green, A. Ebaee, V. Feichtenschlager, K. Chong, L. Peng, M. T. Dimon, T. Phillips, A. I. Daud, T. H. McCalmont, P. E. LeBoit, S. Ortiz-Urda, Combined targeting of MEK and PI3K/mTOR effector pathways is necessary to effectively inhibit NRAS mutant melanoma in vitro and in vivo. *Proc. Natl. Acad. Sci. U.S.A.* **110**, 4015–4020 (2013).

162. H. Shi, X. Kong, A. Ribas, R. S. Lo, Combinatorial treatments that overcome PDGFRβ-driven resistance of melanoma cells to V600EB-RAF inhibition. *Cancer Res.* **71**, 5067–5074 (2011).

163. J. Müller, O. Krijgsman, J. Tsoi, L. Robert, W. Hugo, C. Song, X. Kong, P. A. Possik, P. D. M. Cornelissen-Steijger, M. H. G. Foppen, K. Kemper, C. R. Goding, U. McDermott, C. Blank, J. Haanen, T. G. Graeber, A. Ribas, R. S. Lo, D. S. Peeper, Low MITF/AXL ratio predicts early resistance to multiple targeted drugs in melanoma. *Nat. Commun.* **5**, 5712 (2014).

164. N. Wagle, E. M. van Allen, D. J. Treacy, D. T. Frederick, Z. A. Cooper, A. Taylor-Weiner, M. Rosenberg, E. M. Goetz, R. J. Sullivan, D. N. Farlow, D. C. Friedrich, K. Anderka, D. Perrin, C. M. Johannessen, A. McKenna, K. Cibulskis, G. Kryukov, E. Hodis, D. P. Lawrence, S. Fisher, G. Getz, S. B. Gabriel, S. L. Carter, K. T. Flaherty, J. A. Wargo, L. A. Garraway, MAP kinase pathway alterations in BRAF-mutant melanoma patients with acquired resistance to combined RAF/MEK inhibition. *Cancer Discov.* **4**, 61–68 (2014).

165. E. M. Van Allen, N. Wagle, A. Sucker, D. J. Treacy, C. M. Johannessen, E. M. Goetz, C. S. Place, A. Taylor-Weiner, S. Whittaker, G. V. Kryukov, E. Hodis, M. Rosenberg, A. M. Kenna, K. Cibulskis, D. Farlow, L. Zimmer, U. Hillen, R. Gutzmer, S. M. Goldinger, S. Ugurel, H. J. Gogas, F. Egberts, C. Berking, U. Trefzer, C. Loquai, B. Weide, J. C. Hassel, S. B. Gabriel, S. L. Carter, G. Getz, L. A. Garraway, D. Schadendorf; Dermatologic Cooperative

Oncology Group of Germany (DeCOG), The genetic landscape of clinical resistance to RAF inhibition in metastatic melanoma. *Cancer Discov.* **4**, 94–109 (2014).
